# An Affordable Topography-Based Protocol for Assigning a Residue’s Character on a Hydropathy (PARCH) Scale

**DOI:** 10.1021/acs.jctc.3c00106

**Published:** 2023-04-05

**Authors:** Jingjing Ji, Britnie Carpentier, Arindam Chakraborty, Shikha Nangia

**Affiliations:** † Department of Biomedical and Chemical Engineering, Syracuse University, Syracuse, New York 13244, United States; ‡ Department of Chemistry, Syracuse University, Syracuse, New York 13244, United States

## Abstract

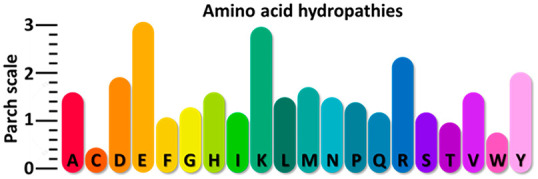

The hydropathy of proteins or quantitative assessment of protein–water interactions has been a topic of interest for decades. Most hydropathy scales use a residue-based or atom-based approach to assign fixed numerical values to the 20 amino acids and categorize them as hydrophilic, hydroneutral, or hydrophobic. These scales overlook the protein’s nanoscale topography, such as bumps, crevices, cavities, clefts, pockets, and channels, in calculating the hydropathy of the residues. Some recent studies have included protein topography in determining hydrophobic patches on protein surfaces, but these methods do not provide a hydropathy scale. To overcome the limitations in the existing methods, we have developed a Protocol for Assigning a Residue’s Character on the Hydropathy (PARCH) scale that adopts a holistic approach to assigning the hydropathy of a residue. The parch scale evaluates the collective response of the water molecules in the protein’s first hydration shell to increasing temperatures. We performed the parch analysis of a set of well-studied proteins that include the following—enzymes, immune proteins, and integral membrane proteins, as well as fungal and virus capsid proteins. Since the parch scale evaluates every residue based on its location, a residue may have very different parch values inside a crevice versus a surface bump. Thus, a residue can have a range of parch values (or hydropathies) dictated by the local geometry. The parch scale calculations are computationally inexpensive and can compare hydropathies of different proteins. The parch analysis can affordably and reliably aid in designing nanostructured surfaces, identifying hydrophilic and hydrophobic patches, and drug discovery.

## Introduction

Proteins, the workhorses of biological systems, exhibit chemical and topographical heterogeneity to perform life functions. The chemical variations in the primary sequence of amino acids coupled with secondary and tertiary folding patterns create proteins with infinite topographies, such as bumps, curvatures, crevices, cavities, clefts, pockets, pores, and channels.^1^ The surface chemistries and topography interplay generate unique nanoscale interactions between the proteins and the surrounding water.^2−7^ Subtle differences in the side chain chemistry of the amino acids or local geometry can lead to substantial differences in protein’s affinity to water. However, accurately identifying hydrophilic and hydrophobic patches on a topologically rough protein surface has remained a challenge.

In the past six decades, numerous protein hydropathy scales have been reported.^8−54^ Most scales assign a fixed value to an amino acid residue on the hydrophobic scale based on some physical or chemical property of the residue. These properties vary from the surface area of the residue,^12,14,27,42^ octanol–water distribution coefficient,^22,25,39^ residue–residue contact statistics,^20,29^ hydration free energy of a residue,^13,28,32−34,37,45,46,50^ entropic cost of solvation,^8,40,49,51−53^ water orientation around a residue,^47,54^ the surface tension of the water surrounding a residue,^15^ and several others.^9−11,16−19,21,23,24,26,30,31,35,36,38,41,43,44,48^ Despite multiple strategies, these scales fail to capture the hydropathy of protein surfaces accurately.

Advances have been made to use the water density fluctuation-based approach in specialized molecular simulations to identify hydrophobic patches on the surface of proteins.^4,55−62^ This computational framework successfully captures the nanoscopic chemical and topographical context of the protein surfaces. These studies have demonstrated that the hydrophobic patches contain residues with polar and nonpolar side chains, highlighting the rich chemical heterogeneity in hydrophobic patches.^60,61^ Since this work evaluates the protein in its entirety, it does not assign a hydropathy value to the residues that constitute the hydrophobic patch or compare them to other residues on the protein surface.

The complex interplay of chemical and topological heterogeneity has been observed in synthetically designed, subnanometer-sized, bowl-shaped molecules called cavitands.^63−68^ Depending on the orientation of the functional (methyl) groups on the rim of the molecule, the cavitand bowl can be water-filled or water-devoid.^67,68^ The cavitand hydropathy has been a focus of several collaborative computational and experimental studies. Other experimental work has also emphasized the dependence of the hydrophobic effect on both surface compositions and subtle surface-shape variations.^69−73^ However, such synergistic collaborations focusing on the hydrophobicity of complex protein surfaces have been unable to make a breakthrough, and accurate experimental characterization of the hydropathy of proteins has remained a challenge.

To overcome the limitations of the current approaches, we have developed a new Protocol for Assigning a Residue’s Character on a Hydropathy (PARCH) scale. The parch scale evaluates the collective response of the water molecules in the protein’s first hydration shell to increasing temperatures. Since the water molecules envelop the protein’s surface, their retention or evaporation from a residue reflects the chemical and topographical heterogeneity of the protein’s surface. In the parch scale, residues that readily lose water (hydrophobic) are assigned low parch values, while residues that tend to retain water (hydrophilic) are assigned high parch values.

In this work, we report the parch analysis of a representative set of 14 proteins, including enzymes,^74−78^ immune proteins,^79^ integral membrane proteins,^80−84^ and fungal and virus capsid proteins.^85,86^ The diversity in the chemistry, structure, and function of the proteins in the set allowed us to investigate the accuracy of the parch scale method. We were able to determine the hydrophobic thickness of the membrane proteins accurately. The parch scale accurately predicted the patch’s residues in proteins with known hydrophobic patches. Unlike previous hydropathy scales, our results showed that the 20 amino acids have no fixed hydropathy value. For example, a charged residue like glutamate can exhibit a low affinity to water if partially embedded in a crevice or surrounded by residues with low water affinity. Thus, each amino acid can show a range of parch values depending on its local environment in a protein.

The parch scale calculations employ all-atom molecular dynamics simulations of the protein surrounded by explicit water molecules and counterions. The simulation workflow is fully automated, and it requires only the protein’s structure as an input. The method can determine the hydropathy of a single amino acid residue or a macromolecular protein. Recently, we used this approach to determine the hydropathy of lipid-modified intrinsically disordered protein.^87^ The calculations are computationally affordable and have been tested for several proteins available in the protein data bank.

We envision the parch scale approach to have multiple applications in protein folding,^88,89^ protein–protein association,^8,90−92^ protein–ligand binding,^93^ signal transduction,^94^ and enzyme catalysis.^95^ In our recent work, we used the fundamental principles of the parch scale to explain the nanoassembly of lipid-modified intrinsically disordered proteins (IDPs). We showed that hydration heterogeneities influence the hydrophilic/hydrophobic patches and cause these disordered proteins to form nanocarriers that show temperature-dependent structural changes.

## Approach

The parch scale quantifies the tendency of a residue to retain or lose hydration shell water molecules as a function of temperature. Each residue is assigned a parch value without making *a priori* assumptions about the chemical nature or the local topography of the residue. To compute the parch values, we first solvate the protein in a shell of water of uniform density. The water molecules contour the protein’s surface and establish a nanoscale network around the surface residues. We then count the number of water molecules contacting each residue within a predetermined cutoff. The water count provides a baseline estimate of the geometrical space available to a residue. Depending on the residue’s location in a crevice or on the surface, its water count can be significantly different. Thus, the initial water count of a residue is purely dictated by the protein’s topography and does not involve any dynamics.

To invoke the effect of chemical heterogeneity of the protein, we perform nonequilibrium annealing simulations by increasing the temperature of the system to “evaporate” the water from the protein’s surface. The protein is position-restrained during annealing to prevent conformational changes in the protein or orientational changes in side chain residues. Therefore, the protein structure should be equilibrated to mimic the *in vitro* or *in vivo* conditions prior to the annealing simulations.

During parch scale calculations, the protein dehydrates with an increase in the annealing temperature, but the loss of water is not uniform; it is residue- and topography-specific. Water is lost more readily from the regions with weak protein–water interactions than the regions with strong interactions with water. For example, a residue with a small aliphatic side chain may lose its water molecules at a lower temperature than a residue with an aromatic side chain. Similarly, an uncharged residue may lose water at earlier than a charged residue. The water count of each residue is recorded at regular intervals during the annealing process. Since each residue is unique, we use the water count data to compute the time average of the autocorrelation function.^96,97^ Finally, we compute the parch value, which is a ratio of the average autocorrelation value of the residue to a reference residue.

The stepwise details of the parch scale calculations are as follows:

1. The equilibrated protein structure is solvated with a shell of water. The water molecules undergo annealing (low to high temperature) at constant volume, while the protein is position restrained. The annealing rate (temperature per unit simulation time) was kept constant.
2. At regular time intervals during the annealing process, we determine the number of water molecules within the cutoff, *d*_water_, from a residue. The quantity, *w*_*i*_(*t*), represents the number of water molecules surrounding the *i*^th^ residue of the protein at time, *t*.
3. We impose a monotonicity condition on the time series {*w*_*i*_(*t*)} and define a new variable *η*_*i*_(*t*):
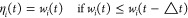

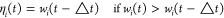
This ensures that *η*_*i*_(*t*) does not have noise in the water count as the annealing temperature increases.
4. Using *η*_*i*_(*t*), we define the autocorrelation function *C*_*i*_(τ) as
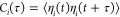
5. The average of the autocorrelation function is obtained from the time integral
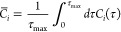
6. The parch value, *PV*_*i*_, for the *i*^th^ residue is obtained from the ratio of *C̅*_*i*_ with respect to a reference residue *C̅*_ref_
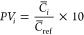

To develop the parch scale, we divide the average autocorrelation *C̅*_*i*_, with respect to a reference residue. Using a reference residue standardizes the parch analysis against an external residue, standardizes the parch values, and allows for a seamless comparison of residues in different proteins. The quantity, *C̅*_*i*_/*C̅*_ref_, has a value between 0 and 1, but to expand our parch values on a broader scale, we multiply the quantity by 10. Thus, a solvent-exposed residue with high chemical affinity to water, equal to the reference residue, has a parch value of 10; however, if the same residue was buried in the protein with no access to water, it has a parch value of zero. Also, a surface residue with no chemical affinity to water has zero parch value. To identify a reference, we individually computed the *C̅*_*i*_ values of the 20 single amino acid residues. The details of the single amino acid calculations and the selected reference is provided in the Methods section.

As noted above, the parch method makes no *a priori* assumptions about the chemical nature or the structure of the target molecule in computing the hydropathy. The parch value represents a residue’s affinity for water based on its chemical and topographical location. Thus, unlike other residue-based hydropathy scales, the parch method does not assign a value to the residue solely based on its chemical identity. Moreover, the parch analysis is performed for a protein in a fixed conformation. If a protein has multiple biologically relevant conformations, each conformation will require a separate parch scale calculation. The method is computationally affordable because parch calculation requires only a shell of water around an equilibrated protein. The algorithm used for the calculations is independent of the force field used to model the system. Although the hydropathy of proteins has been the focus of the present work, the approach used for parch scale calculations can be extended to other classes of molecules.

## Methods

### Protein Equilibration

In this study, 14 proteins were modeled (Table 1). The initial protein structures were obtained from the Protein Data Bank when available. Each protein was solvated in a 0.15 M NaCl solution using the solution builder utility on the CHARMM-GUI web server.^98−100^ The CHARMM36m force field^101^ parameters were used to model the proteins and the ions, and TIP3P water parameters were used to model the water. All-atom molecular dynamics (MD) simulations were performed using GROMACS 2019.^102^ The systems were energy minimized using the steepest decent algorithm and equilibrated in the isothermal-isochoric (*NVT*) and isothermal–isobaric (*NPT*) ensemble conditions for 2 ns each. The heavy atoms of the proteins were restrained during the *NVT* and *NPT* runs. The temperature was maintained at 300 K using the v-rescale thermostat^103^ with a temperature coupling constant, τ_t_ = 1.0 ps. In the *NPT* run, isotropic pressure of 1 bar was maintained using the Berendsen barostat^104^ with the pressure coupling constant, τ_p_ = 5.0 ps, and the compressibility constant of 4.5 × 10^–5^ bar^–1^. A 2 fs time step was used, and the nonbonded interaction neighbor list was updated every 20 steps. A 1.2 nm cutoff was used for the electrostatic and van der Waals interactions. The long-range electrostatic interactions were calculated using the Particle-Mesh Ewald (PME) method after a 1.2 nm cutoff. The bonds involving hydrogen atoms were constrained using the linear constraint solver (LINCS) algorithm. An additional 10 ns production MD run was performed under NPT conditions using the Parrinello–Rahman barostat^105^ with τ_p_ = 5.0 ps and compressibility of 4.5 × 10^–5^ bar^–1^. All restraints were removed during the production MD.

**Table 1:** Details of the Proteins Investigated

protein	PDB ID	charge	monomer/dimer	function
bacteriophage T4 lysozyme (LYM)	253L	9	M	enzyme
thymidylate synthase (TS)	2TSC	–10	M	enzyme
malate dehydrogenase (MDH)	3HHP	–3	M	enzyme
barnase^a^ (BNS)	1BRS	2	M	enzyme
mouse double minute 2 (MDM2)^b^	1YCR	1	M	enzyme
mannose-binding protein (MBP)	1MSB	–4	M	plasma, immune
		–8	D	
hydrophobin II (HP2)	2B97	0	M	fungi, structural
hepatitis B viral capsid (HBV)	1QGT	–7	M	viral capsid, structural
		–14	D	
melittin (MLT)	2MLT	10	D	membrane-active
claudin-5 (CLD5)^c^		0	M	membrane
aquaporin (AQP1)	1J4N	3	M	membrane
ghrelin O-acyltransferase (hGOAT)^c^		9	M	membrane, enzyme

a In the absence of barstar.

b In the absence of p53.

c The structure was obtained from previous homology or modeled systems.^80−83^

### Simulation Setup and Optimization

The equilibrated protein was extracted and placed into an empty cubic simulation box. The protein was then rehydrated with a shell of water (average density 0.977 g cm^–3^) of thickness *d*_shell_, larger than the first hydration shell of water from the protein’s surface. To determine the thickness of the water shell, *d*_shell_, we performed radial distribution calculations of water for all 14 proteins (Figure S1). In all cases, water was well-structured around the proteins in first, second, and third hydration shells at an average distance of 0.315, 0.415, and 0.515 nm, respectively. We selected *d*_shell_ = 0.415 nm, which is the thickness of the second hydration shell in the radial distribution functions of the proteins.

**Figure 1:**
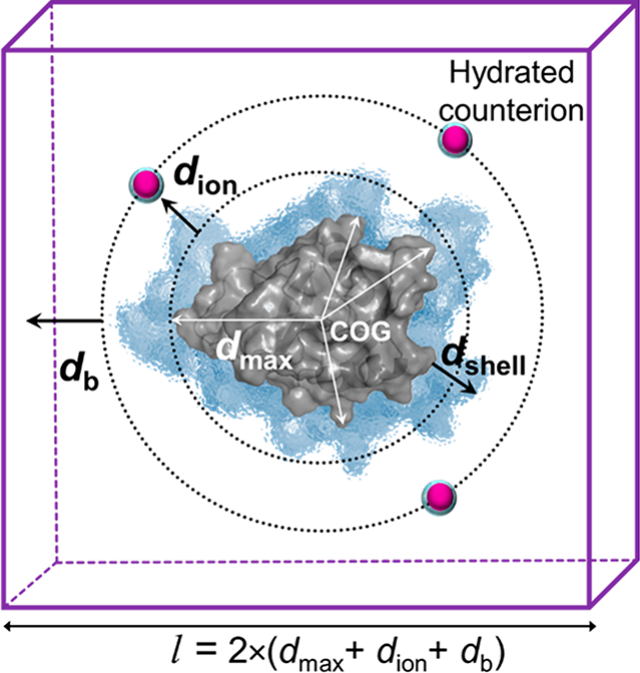
Schematic of the cubic simulation box before the parch annealing step. The protein (gray) of maximum radius, *d*_max_, placed in a box of length *l* is surrounded by a shell of explicit water, *d*_shell_, with hydrated counterions (pink spheres) at a fixed distance, *d*_ion_, from the protein’s surface. The cell boundary is at a distance, *d*_b_, from the hydrated counterion.

The edge length, *l*, of the simulation box was computed based on three input parameters: size of the protein (*d*_max_), the distance of the counterions from the protein’s surface (*d*_ion_), and the distance between the ions and the box boundary (*d*_b_). A definition of these parameters and their selected values for the calculations are provided in Table S1. To compute *d*_max_, we determined the maximum distance between the center of geometry (COG) of the protein and the COG of each amino acid residue in the protein. A schematic of a hydrated protein and the counterions in the simulation box size is shown in Figure 1. Hydrated counterions (Na^+^ or Cl^–^) were placed at a distance, *d*_ion_, from the surface of the charged protein, defined by *d*_max_. We determined the optimal placement of the hydrated ions from the protein surface by varying the *d*_ion_ values from 3.0 to 5.0 nm with 0.5 nm increments while maintaining the interion distance of at least 4.0 nm. Finally, the size of the simulation box was determined, such that the distance between the ion and the box boundary, *d*_b_, is 3.0 nm, in order for the minimum distance between the periodic images to be at least 6.0 nm. Taken together, the simulation box size is twice the additive distances of *d*_max_, *d*_ion_, and *d*_b_.

The optimal values of parameters *d*_ion_ = 3.0 nm and *d*_b_ = 3.0 nm were determined after a series of performance tests using BNS and TS proteins; the results and discussion of the performance tests are provided in the Supporting Information (Figures S2 and S3). Next, we determined the cutoff for the number of water molecules, *d*_water_, contacting a residue at any given time. The optimal value of *d*_water_ = 3.15 nm was selected after testing different cutoff values (Figure S4).

The new protein systems were energy-minimized, followed by water evaporation using the GROMACS annealing protocol. Each protein system was annealed from 300 to 800 K at a constant volume with an annealing rate of 1 K/10 ps. Two additional annealing rates, 1 K/1 ps and 1 K/100 ps, were tested, and the discussion on the choice is presented in the Supporting Information (Figures S5 and S6).

During the annealing process, water molecules were allowed to move within the fixed volume of the box without any positional constraints. However, the protein was position constrained with a force constant of 1000 kJ mol^–1^ nm^–2^ to maintain the protein’s conformation and topography. Similarly, the counterions were restrained with a force constant of 1000 kJ mol^–1^ nm^–2^ to maintain a constant charge balance around the protein. The results and discussion of two additional force constants tested, 500 and 1500 kJ mol^–1^ nm^–2^, are provided in the Supporting Information (Figure S7).

The rehydration and annealing simulations were performed in triplicates. The wall-clock times for the parch scale calculations for the proteins studied in this work are provided in the Supporting Information (Figure S8).

Besides the test proteins, the equilibration, extraction, rehydration, and annealing processes were performed for the zwitterionic forms of all 20 single amino acids in triplicates. The rationale for performing hydropathy calculations for the single amino acid was to determine the reference residue *C̅*_ref_. We found lysine had the highest value, *C̅*_ref_ = *C̅*_lysine_, and so, it was chosen as the reference; therefore, the single lysine residue has a parch value of 10. Table S2 lists the parch values for the 20 single amino acids in an increasing order.

### Parch Output and Visualization

The computed parch values for each residue are written in a text file. To facilitate visualization in software suites like PyMOL,^106^ VMD,^107^ and YASARA,^108^ the parch values are also written in the occupancy column of an output PDB file.

## Results

We report the parch analysis of structurally and functionally distinct proteins (Table 1) examined in the literature for their hydropathy, structure, or function. We have subdivided the proteins into three categories: soluble proteins, proteins with hydrophobic patches, and membrane or membrane-penetrating proteins.

### Hydropathy of Soluble Proteins

**Bacteriophage lysozyme T4 (LYM)** is an antimicrobial protein that hydrolyzes the bacterial cell wall.^109,110^ It belongs to a large family of lysozymes in eukaryotes that destroy bacteria to provide immunity to its host. Of the 164 residues, 43 (26%) are charged, which help lysozyme bind to the highly charged bacterial peptidoglycan layer and break down the glycosidic bonds.

**Figure 2:**
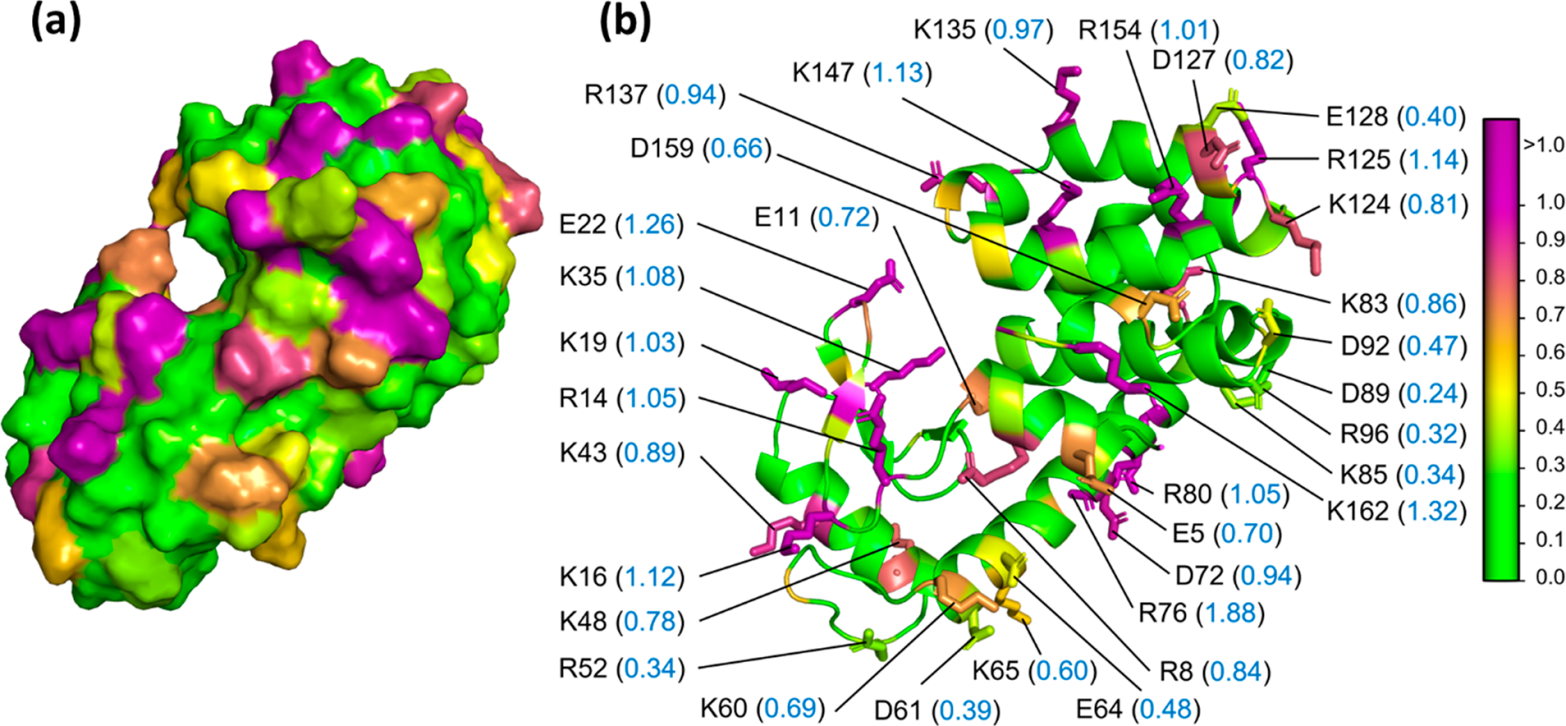
Hydropathy of bacteriophage lysozyme T4 (LYM). PDB: 253L. (a) Surface representation showing the distribution of residues with different parch values. (b) Cartoon structure with parch values (blue font) of charged surface accessible residues (side chains shown as sticks). The residues are colored based on the parch values on the color scale (right).

The lysozyme hydropathy analysis shows residues with high parch values distributed all over the protein’s surface (Figure 2a). The average parch value of the 26 positively charged (R and K) residues and 17 negatively charged (D and E) residues is 0.84 and 0.4 (Figure 2b). Overall, lysozyme is a hydrophilic, soluble protein with high surface charge (Table S3).

**Thymidylate synthase (TS)** is a dimeric enzyme essential for DNA synthesis, repair, and cell survival. The enzyme catalyzes reductive methylation of deoxyuridine monophosphate to thymidylate using 5,10-methylenetetrahydrofolate as the methyl group donor. Inhibition of TS has been of interest for cancer chemotherapy as it causes DNA damage and induces cell death.

**Figure 3:**
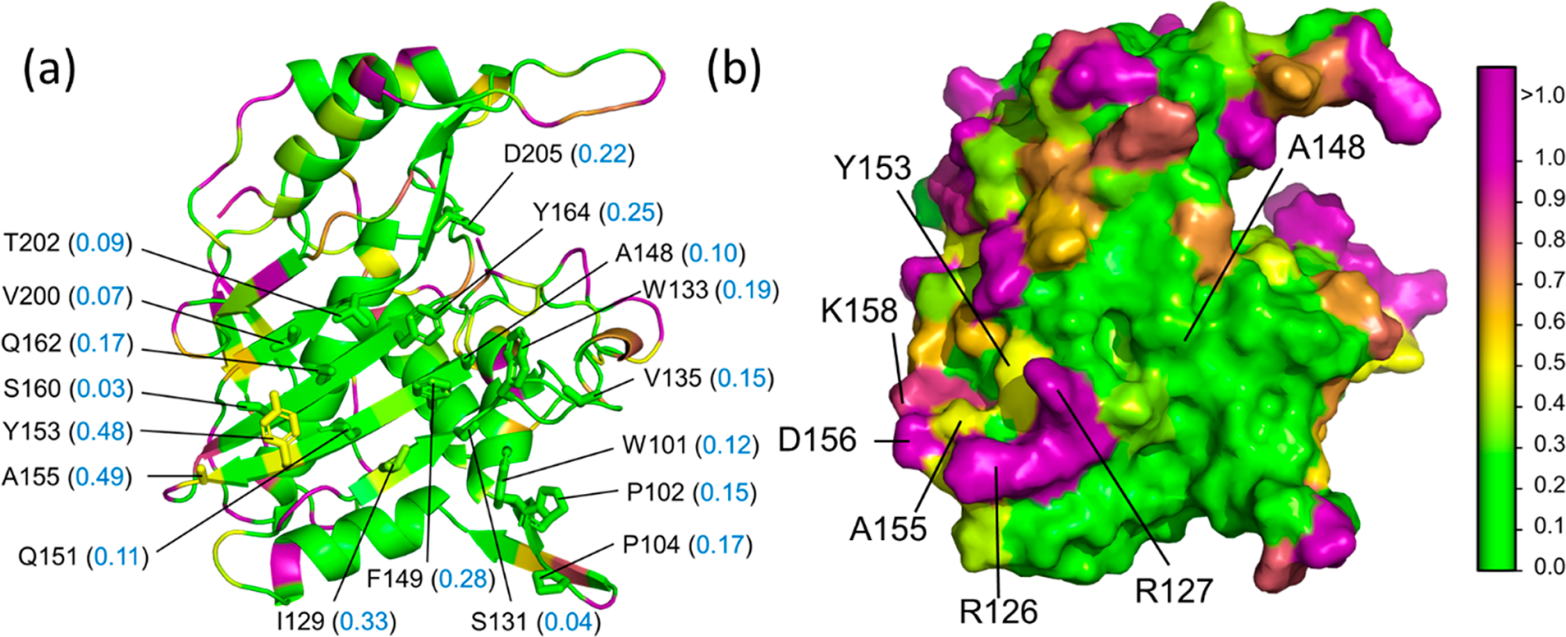
Hydropathy of the thymidylate synthase (TS) monomer. PDB: 2TSC. (a) Cartoon view of the TS monomer that interacts with another monomer to form the TS homodimer. Key interfacial residues (stick representation) and the parch values (blue font, in parentheses) are shown. (b) Surface view of the A155 residue surrounded by charged hydrophilic residues—R126, R127, D156, and K158. The residues are colored based on parch values on the color scale (right).

The TS dimer comprises symmetric monomers that interact via specific interactions.^76^ In a recent paper, Rego et al. studied the TS monomer to identify the hydrophobic patch responsible for dimer stability.^61^ We performed the parch analysis of the TS monomer to test our method. Figure 3a shows the parch values of the key residues on the TS dimer interface. These interfacial residues include small (A, V, I), aromatic (W, Y, F), charged (D), and neutral (Q, P, T, S) amino acids. Despite the chemical nature of the residues, their parch values are less than 0.3, which matches the reported hydrophobic character of the interfacial patch. The residues bordering this patch have higher parch values; for example, Y153 and A155 have parch values of 0.48 and 0.49. Parch values of all TS residues are provided in Table S4.

It is noteworthy that an alanine residue can have a low parch value (for example, A148) when it is part of the hydrophobic patch or have a higher parch value (for example, A155) when it is bordering the patch. Figure 3b shows that A148 (0.1) lies on a flat beta-sheet structure and is surrounded by other hydrophobic residues. In contrast, A155 (0.49) lies at the beta-turn and is proximal to R126, R127, D156, and K158 residues with parch values in the 0.8–2.2 range (Figure 3b). This analysis also shows that chemical identity should not be the sole criterion when assigning hydrophilic, hydrophobic, or hydroneutral character to a residue.

**Malate dehydrogenases (MDH),** a ubiquitous enzyme in prokaryotes and eukaryotes, has critical metabolic activity in the citric acid cycle. It catalyzes the conversion of malate and oxaloacetate using nicotinamide adenine dinucleotide (NAD+)/nicotinamide adenine dinucleotide phosphate (NADP+) as a cofactor.

MDH forms a homodimer in *Escherichia coli*. In the crystal structure (PDB: 3HHP), the dimer interface is formed via multiple charged (R153, K217, D45, and E212) and uncharged (P50, T156, T224, and S226) residues with contributions from both monomers. The two monomers interface such that S226 of one monomer lies close to D45 of the second monomer.

The interaction is highly specific, and a mutation of either D45 or S226 causes reduced enzyme activity.^111,112^ Compared to the wild type, a D45Y mutation leads to dimer dissociation and a 14000-fold reduction in enzyme activity, while the S226Y mutation retains the dimer but causes a 1.4-fold reduction in the enzyme activity. However, a D45Y/S226Y double mutation leads to 17,500-fold reduced enzyme activity.

**Figure 4:**
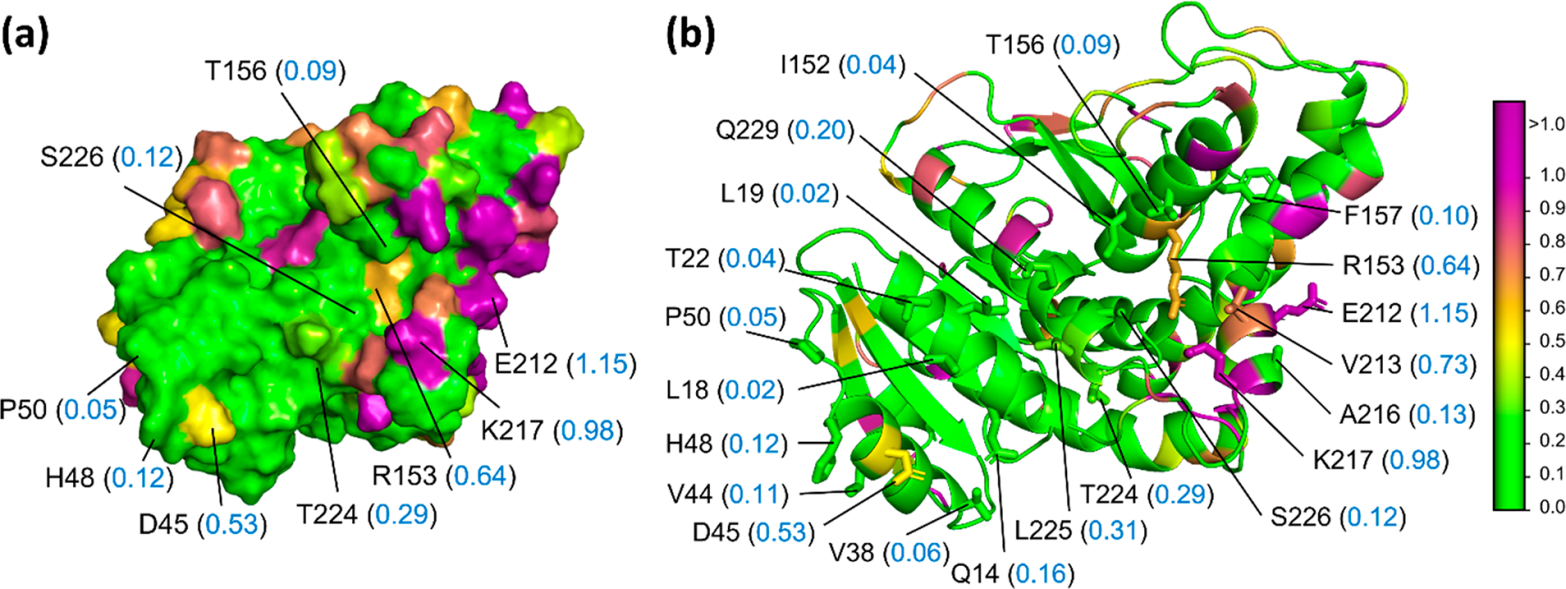
Hydropathy of malate dehydrogenase (MDH). PDB: 3HHP. The (a) surface and (b) cartoon representation and the parch values (blue font; parentheses) of key interfacial residues. The residues are colored based on their parch values on the color scale (right).

The parch analysis of the MDH monomer reveals the hydrophilic character of the protein face that forms the dimer (Figure 4a). The parch values of the charged residues are in the 0.53 to 1.15 range, indicating a hydrophilic character (Table S5). The parch values of the rest of the interfacial residues are shown in Figure 4b. Overall, the parch analysis confirms the hydrophilic nature of the MDH surface—electrostatic interactions stabilize the dimer interface.

**Barnase (BNS)**, a small bacterial enzyme (110 residues), causes ribonucleic acid (RNA) degradation. To inhibit the destructive activity of barnase, another small protein (89 residue), barstar, is produced. The barnase-barstar complex is very stable with dissociation rates lower by 2 orders of magnitude than other protein–protein complexes.^113^ In fact, the toxicity of barnase is observed only in the absence of barstar.

The high stability of the barnase-barstar complex is attributed to their multiple charged interactions.^114^ The crystal structure of the complex shows proximity of the positively charged barnase residues (K27, R59, R83, and R87) and negatively charged barstar residues. Biochemical studies involving the point mutations of these residues show drastic reduction in the stability of the barnase-barstar complex. For example, a barnase R59A mutation led to a 4 orders of magnitude decrease in the barnase-barstar association constant.^115,116^

**Figure 5:**
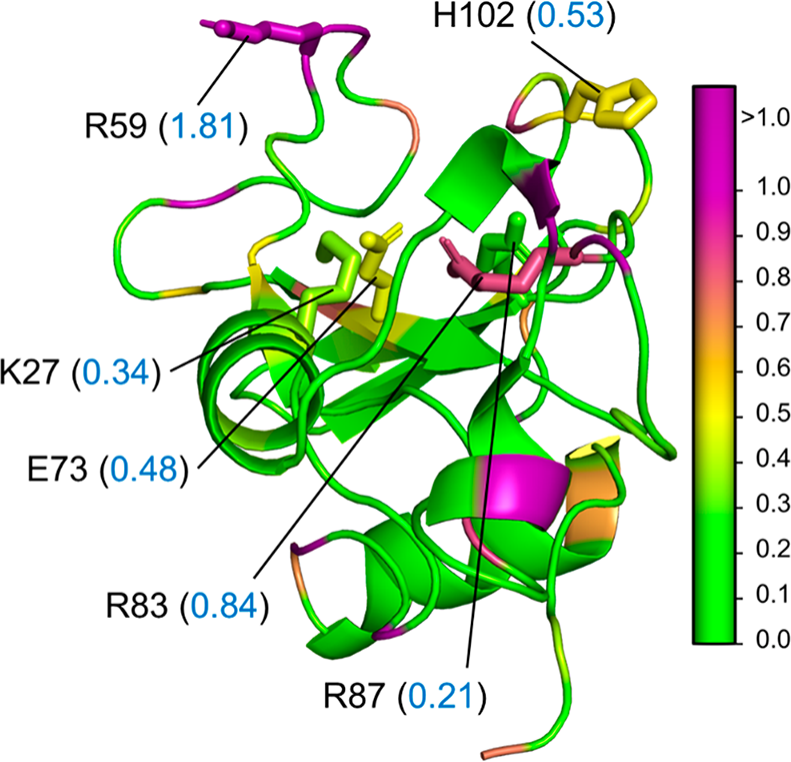
Hydropathy of barnase (BNS). PDB: 1BRS. Side view of barnase showing the charged residues—K27, R59, E73, R83, and R87 (stick representation), and the parch values (blue font, in parentheses). The residues are colored based on their parch values on the color scale provided (right). Barstar was not included in the hydropathy calculations.

Parch analysis of the barnase protein (without barstar) demonstrates the role of surface topography and its charged interface through which it interacts with barstar. In the absence of barstar, the R59 residue is fully exposed to water and has a high parch value of 1.81 that indicates its affinity to water (Figure 5). Other positively charged residues in the interfacial region are topologically less solvent accessible than R59 and, therefore, have lower parch values—K27 (0.34), R83 (0.84), and R87(0.21). There is also a negatively charged E73 residue with a parch value of 0.48 that contributes to the electrostatic interactions of the interface. The majority of the remaining residues has parch values below 0.3 (Table S6). The topography and colocalization of charged residues on one face of barnase results in its stable interaction with barstar.

**Mouse double minute 2 (MDM2)** is a negative regulator protein of the p53 tumor suppressor protein. The p53 protein repairs the damaged DNA and restores cancer cells to healthy states. The MDM2 binds to the N-terminal domain of p53 to inhibit its repair function, thus, causing cancer cell survival and proliferation.

**Figure 6:**
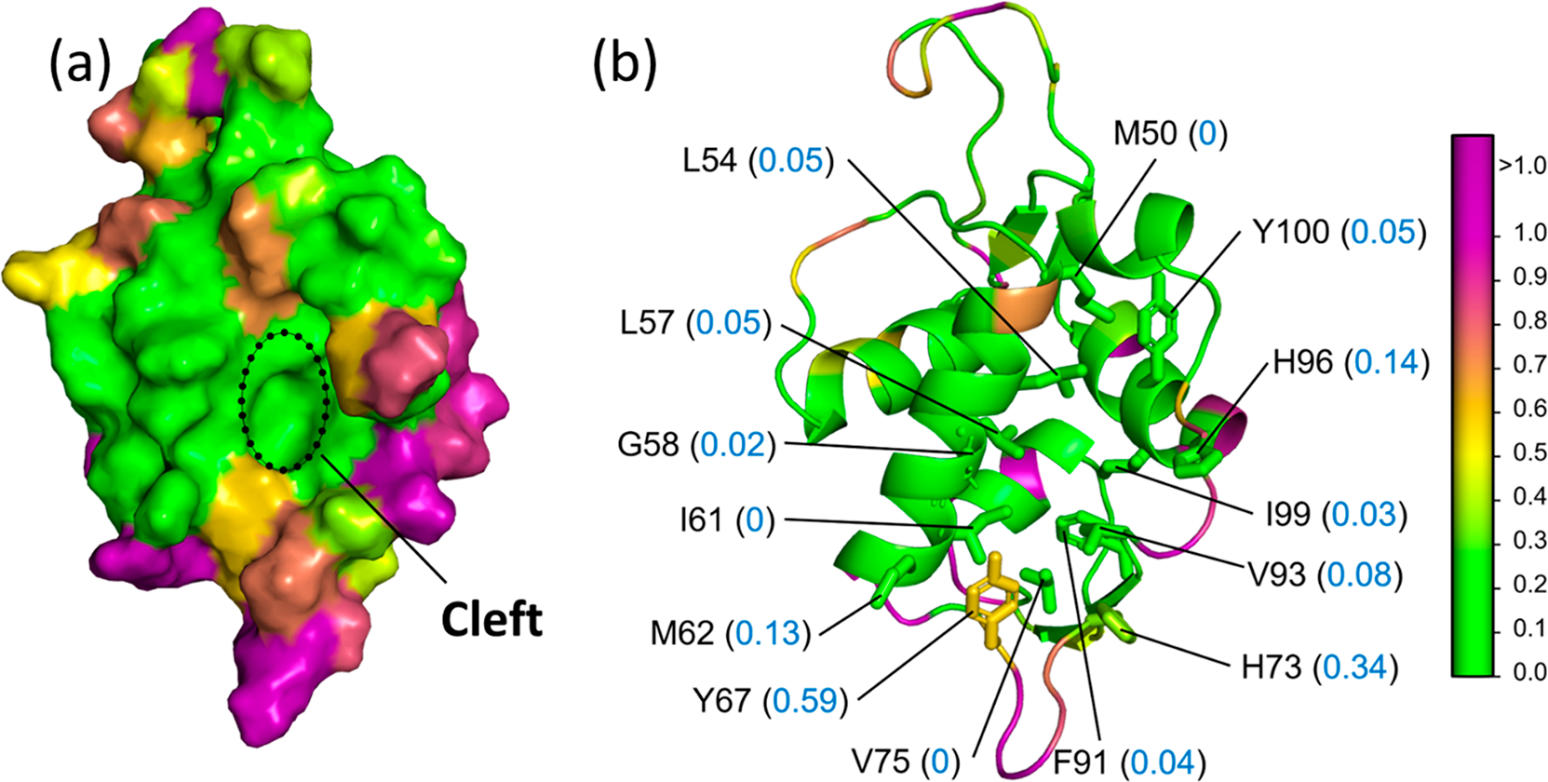
Hydropathy of mouse double minute 2 (MDM2). PDB: 1YCR. (a) Surface representation MDM2 showing the hydrophobic cleft where the p53 triad (F19, W23, and L26) binds in the crystal structure. (b) Cartoon representation showing the parch values (blue font) of the 14 conserved residues (M50, L54, L57, G58, I61, M62, Y67, H73, V75, F91, V93, H96, I99, and Y100) that create the cleft topography. The residues are colored based on parch values on the color scale (right). The p53 peptide was not included in the hydropathy calculations.

MDM2 is a 483-residue protein; however, here, we focus only on the 109 residues of the N-terminal domain of the MDM2 protein that binds to p53 to form the MDM2-p53 complex (PDB: 1YCR). The binding interface involves the insertion of a triad of p53 residues (F19, W23, and L26) into the MDM2’s hydrophobic cleft,^75,117^ which conceals the p53’s transactivation domain. The p53 triad conformation packs perfectly into the MDM2 cleft (Figure 6a).

The MDM2 hydrophobic cleft consists of 14 conserved residues—M50, L54, L57, G58, I61, M62, Y67, H73, V75, F91, V93, H96, I99, and Y100.^75,118,119^ The parch analysis of MDM2 confirms the amphipathicity of the cleft (Table S7). The parch values lie between 0 and 0.14 for all cleft residues except Y67 (0.59) and H73 (0.34). It is well-established that p53’s F19 interacts with MDM2’s I61(0) and G58 (0.02); p53’s W23 with MDM2’s L54 (0.05); and p53’s L26 with MDM2’s I99 (0.03) and Y100 (0.05). Thus, the parch analysis confirms the hydrophobicity of the residues that interact with p53 in the cleft (Figure 6b).

### Hydropathy of Proteins That Self-Assemble via Hydrophobic Patches

**Mannose-binding proteins (MBP)** are calcium ion dependent animal lectins found in the serum. Their function is to recognize the cell-surface of pathogenic bacteria, virus, protozoa, and fungi and trigger the death of these organisms. Structurally, MBPs organize in multimeric structures, where a monomer consists of three intertwined polypeptide chains. Each polypeptide chain consists of a cysteine-rich N-terminal domain, a collagenous domain, an α-helical neck region, and a C-terminal carbohydrate-recognition domain (CRD).^120^

In 1991, Weis et al. expressed and crystallized only the CRD domain (115 residues) in bacteria and found that it formed a dimer (PDB ID: 1MSB).^79^ We used the 1MSB as the starting structure to investigate the hydropathy of the CRD dimer and the CRD monomer by removing one copy of protein. The parch values of all residues in the MBP monomer (M) and dimer (D) are provided in Tables S8 and S9, respectively.

**Figure 7:**
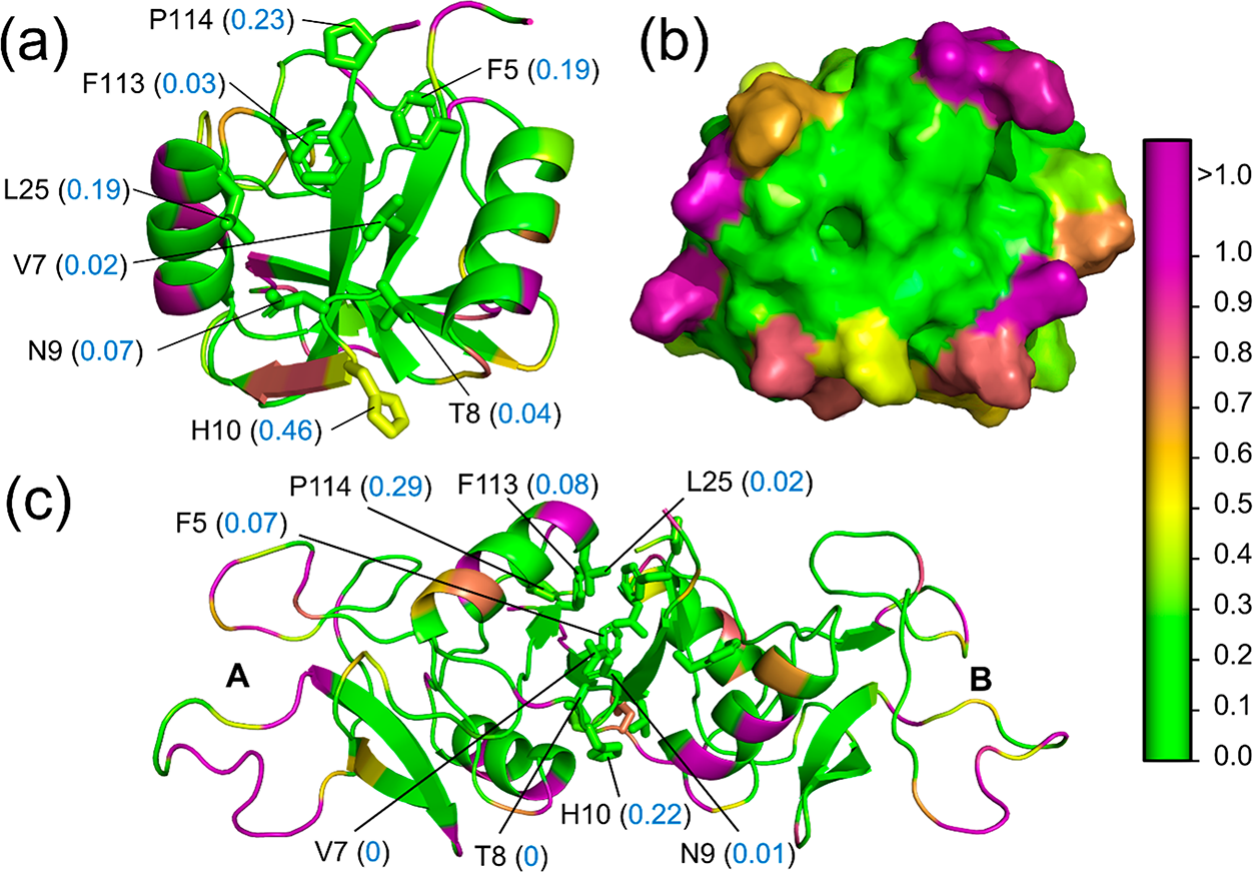
Hydropathy of the mannose-binding protein (MBP) monomer (M) and dimer (D). PDB: 1MSB. The (a) cartoon and (b) surface representations of the MBP monomer. Residues F5, V7, T8, N9, L25, and F113 have low parch values (blue font; in parentheses) and form a topographically flat hydrophobic patch. (c) Side view of the MBP dimer with parch values of the interfacial residues. The parch values of all interfacial residues in the dimer are lower except F113, which is marginally higher. The residues are colored based on parch values on the color scale (right).

The parch data of the CRD monomer revealed a hydrophobic patch of residues (F5, V7, T8, N9, L25, and F113) with parch values < 0.20 (Figure 7a). Additionally, the residues are contiguous and create a flat topology (Figure 7b), suitable for dimer formation. The prediction of the dimer forming interface agrees with the crystal structure of the CRD dimer; two monomers interact head-on via the flat hydrophobic surfaces to create the dimer. Next, we performed the parch analysis of the CRD dimer (Figure 7c). In the dimer, V7 and T8 have a parch value of 0, which shows that they are not solvent accessible. Other interfacial residues have lower parch values than the monomer due to limited solvent access or higher surface hydrophilicity. The rest of the CRD dimer has multiple charged residues (D and E) that have parch values greater than 1.2.

The parch analysis shows that CRD monomers dimerize to hide their hydrophobic patches and thereby increase the solvent accessible area which makes them more water-soluble. Overall, the hydrophilicity of the surface likely makes the CRD Ca^2+^-dependent and soluble in the serum.

**Hydrophobin II (HP2)**, a small protein (71 residues), secreted by fungi, self-assembles to form protective hydrophobic coatings at interfaces. Structurally, hydrophobin II contains four antiparallel β-stands and a short α-helix that are held together by four disulfide bonds. As a result, hydrophobin II is conformationally locked into its tertiary structure and presents a topographically flat surface which is hydrophobic in character. This patch is on one side of the protein, which enables the proteins to adhere to hydrophobic interfaces and form ordered monolayers.^56,85^

**Figure 8:**
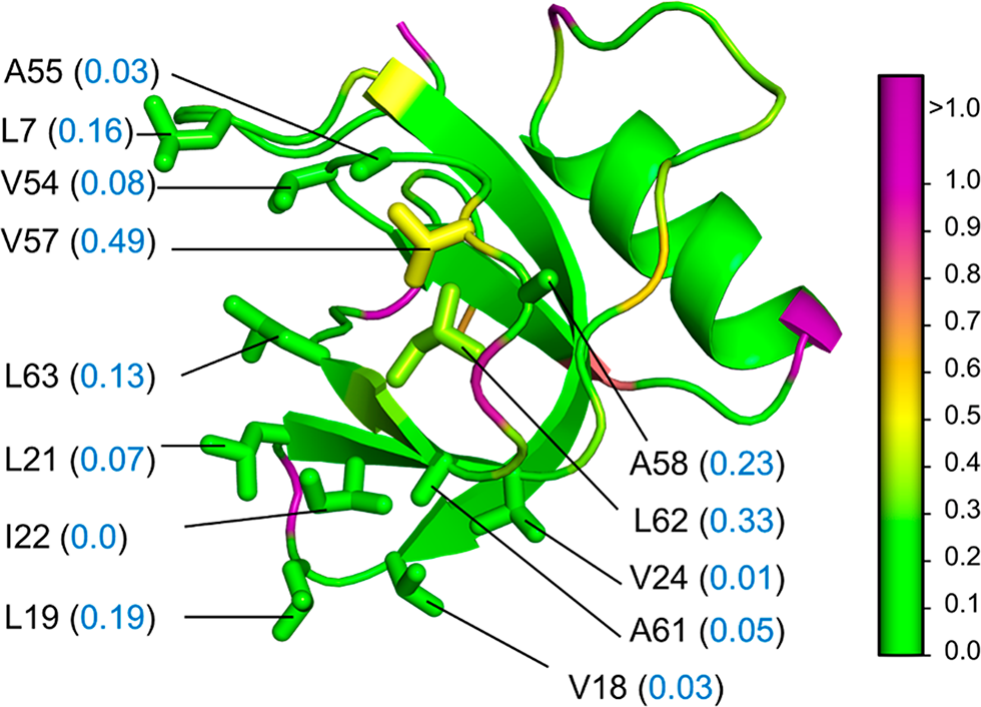
Hydropathy of hydrophobin II (HP2). PDB: 2B97. Cartoon representation of the protein showing the side chains (stick representation) of L7, V18, L19, L21, I22, V24, V54, A55, A58, A61, L62, and L63 residues along with the parch values (blue font, in parentheses). The residues are colored based on their parch values on the color scale provided (right).

The main constituents of the patch are A, L, and V residues that have nonpolar aliphatic hydrocarbon side chains. The parch values of these patch residues L7, V18, L19, L21, I22, V24, V54, A55, A58, A61, L62, and L6 are in the 0–0.33 range (Figure 8). The rest of the protein has some residues with low parch values, but they do not form a contiguous patch (Table S10). Overall, the parch analysis corroborates the known hydropathy of HP2.

**Human hepatitis B virus (HBV)** is an enveloped DNA virus that causes chronic liver disease and cirrhosis. It is a T=4 icosahedral capsid that is formed by 120 copies of a homodimer core protein. The dimer assembles via nonbonded hydrophobic interactions between the monomers. Each HBV monomer is a 183-residue polypeptide containing a 149-residue assembly region that participates in forming the capsid and the remaining 34-residue C-terminal region that interacts with the nucleic acids in the capsid’s core.

**Figure 9:**
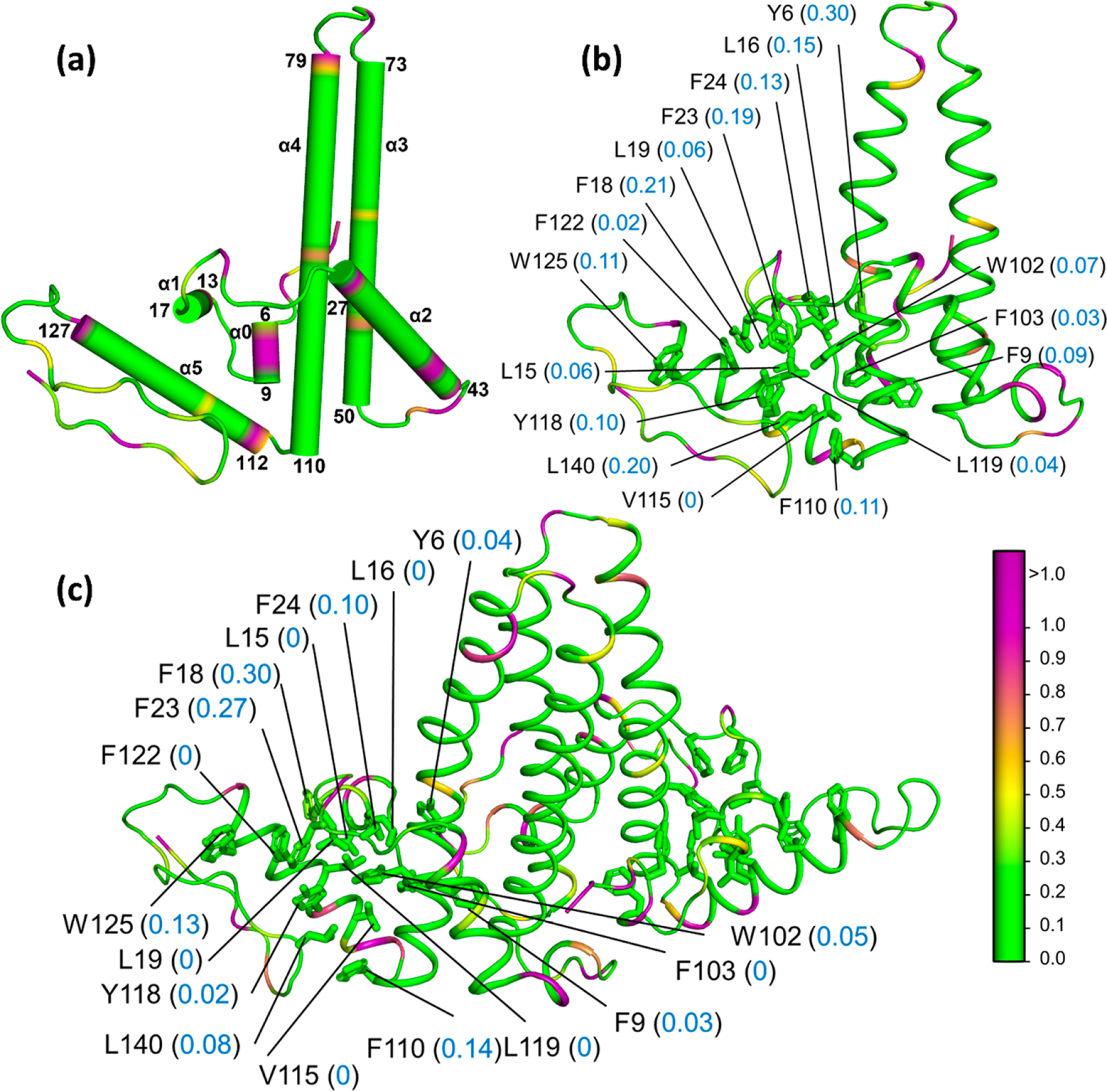
Hydropathy of the human hepatitis B virus (HBV) monomer (M) and dimer (D). PDB: 1QGT. A truncated 149-residue polypeptide (out of 183 residues) was studied. (a) Secondary structure of the HPV monomer showing α1, α2, α3, α4, and α5 helical domains (cylinders) and disordered (coil) regions of the polypeptide. Parch values (blue font) of key C, L, F, and W residues that form the hydrophobic core in the (b) monomer and (c) dimer structures (ribbons). The residues are colored based on parch values on the color scale (right).

The assembly region folds into five alpha helical domains α1 (13–17), α2 (27–43), α3 (50–73), α4 (79–110), and α5 (112–127). The intermittent residues (1–12; 18–26; 44–49; 74–78; and 128–149) are disordered (Figure 9a). The protein is rich in L, F, and W residues, many of which participate in forming the dimer.

Figure 9b shows the parch values of the key residues that participate in dimer formation: N-term (Y6 and F9), short helix α1 (L15, L16), loop connecting α1 and α2 (F18, L19, F23, F24), α4 (W102, F103, F110), α5 (V115, Y118, L119, F122, W125), and extended strand (L140).^86^ These residues have parch values between 0.02 and 0.30 that show the hydrophobicity of the residues. In the HBV dimer, these residues lie in the interfacial region, and in most cases, the parch values of the residues are reduced (Figure 9c). The parch values of the HBV monomer (M) and dimer (D) residues are provided in Tables S11 and S12, respectively.

### Hydropathy of Membrane and Membrane-Embedding Proteins

**Melittin (MLT),** a short helical polypeptide (26 residues), found in bee venom, is a potent cytotoxin. It is a membrane active protein that oligomerizes as dimers, tetramers, and other higher-order complexes to penetrate a cell’s lipid membranes. As a dimer, melittin peptides adopt an antiparallel arrangement where the N-term of one polypeptide interacts with the C-term of the other. The termini are reportedly hydrophilic compared to the center of the dimer which is hydrophobic.^61^

**Figure 10:**
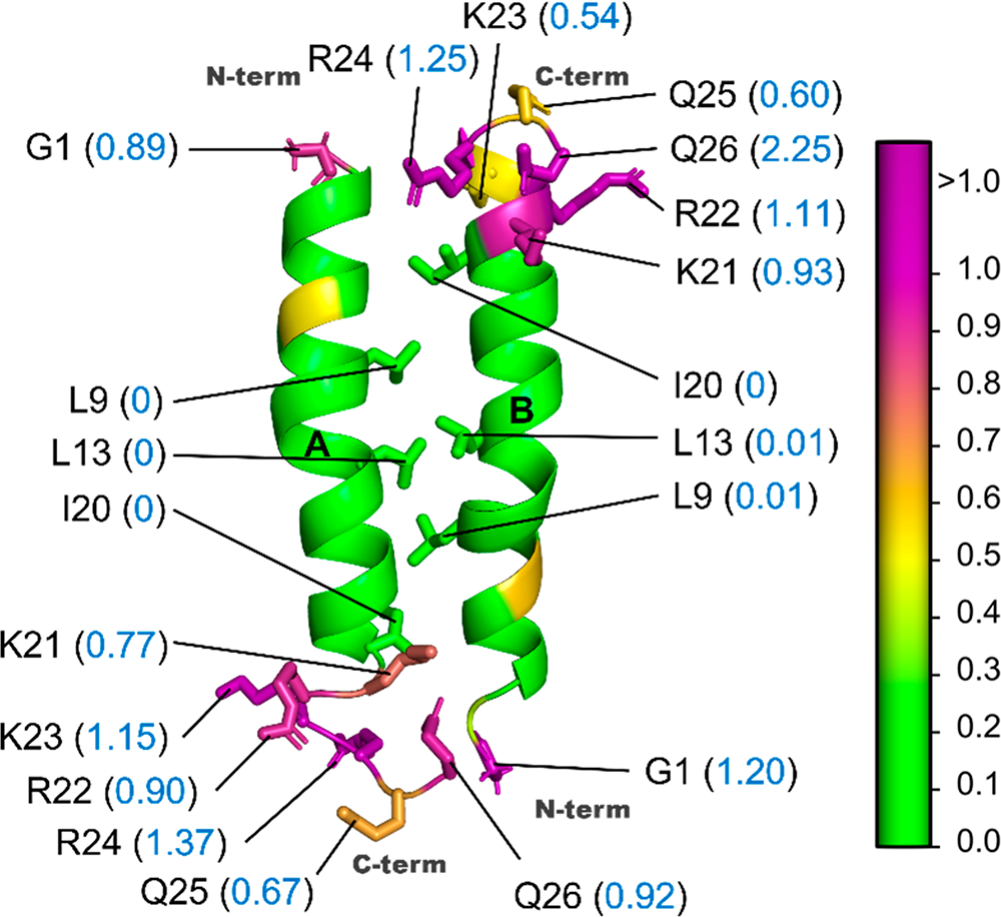
Hydropathy of the melittin dimer (MLT). PDB: 2MLT. Cartoon structure of the antiparallel arrangement of two melittin monomers (A and B). The parch values (blue font) of residues at the N-termini and C-termini are shown. The termini have higher parch values than the helices. The residues are colored based on parch values on the color scale (right).

The parch analysis corroborates with the reported hydropathy of the melittin dimer. We find that melittin’s C-terminal has multiple residues with higher (>0.6) parch values than the N-terminal residues (Figure 10). However, in the antiparallel arrangement, the N-term and C-term pairing makes the termini less hydrophilic than the peptide separately. Figure 10 shows the parch values of the key residues in terminal and center regions of the melittin dimer. The center of dimer has residues like L9, L13, and I20 that have low parch values (0.0 or 0.1) indicating a hydrophobic character. The parch values of the MLT dimer residues are provided in Table S13.

**Claudin-5 (CLD5)**, a transmembrane protein, assembles to form tight-junction strands between adjacent endothelial cells at the blood-brain barrier (BBB) interface. Claudin-5 folds into a four-transmembrane (TM) helix bundle, two extracellular loops (ECL), and an intracellular loop (ICL). The ECL and ICL loops are water accessible and contain charged residues. Unlike other membrane proteins that may oligomerize into dimers or tetramers, claudins interact via the TM and ECL domains to assemble into linear or branched macromolecular structures. The assembly occurs via lateral (or cis) interactions between claudin in a cell membrane and head-to-head (or trans) interactions involving the ECL domains across two adjacent cells.^81−83^

**Figure 11:**
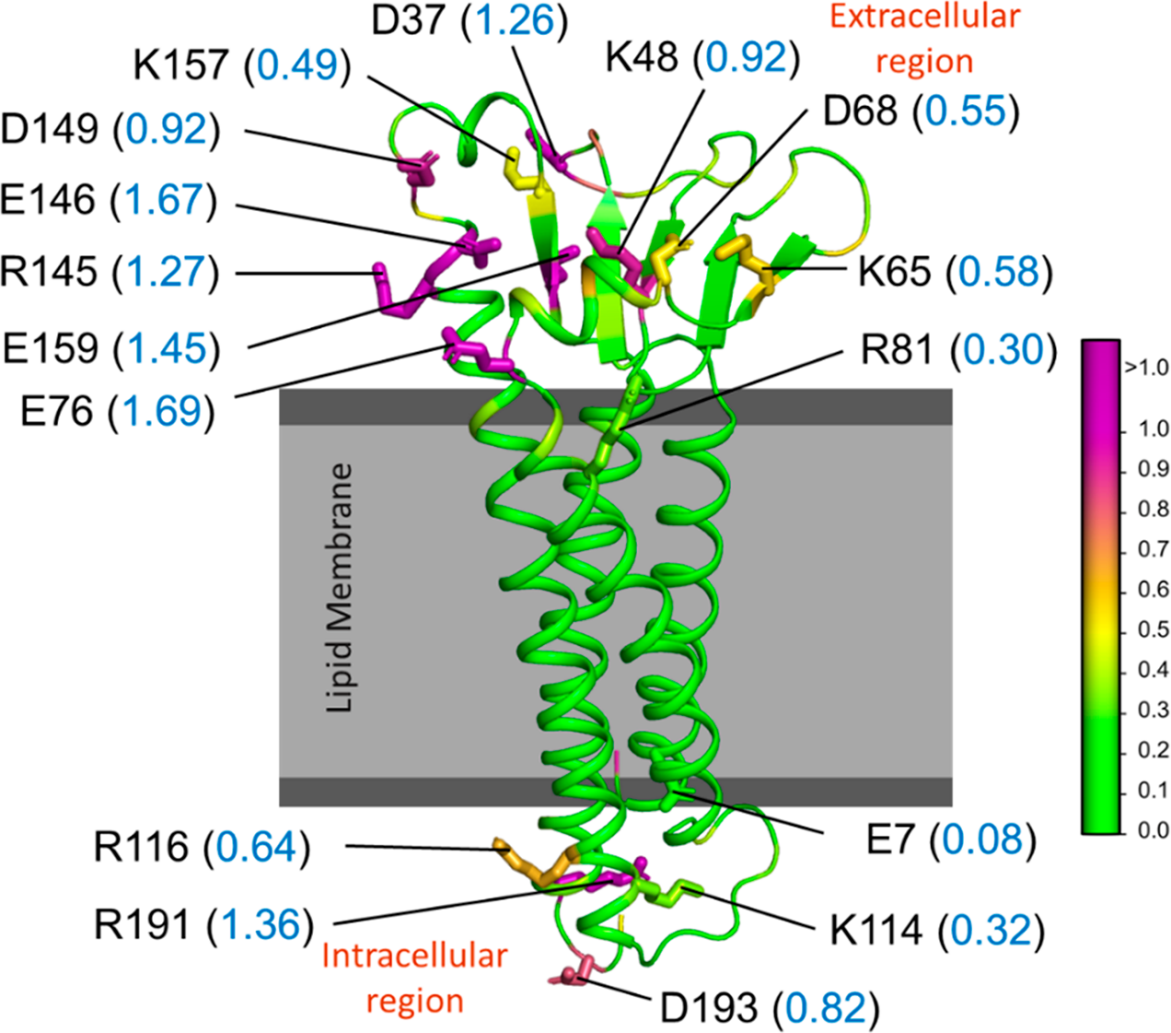
Hydropathy of the claudin-5 protein (CLD5). Cartoon representation of the protein with the extracellular loop (ECL) and intracellular loop (ICL) regions above and below the lipid membrane (gray), respectively. The parch values (blue font) of key ECL and ICL residues (stick representation) are labeled. The residues are colored based on the parch values on the color scale (right).

The parch values of the membrane-embedded and water-exposed residues provide valuable insights into the role of the surface topography of the protein (Figure 11). The TM residues have low parch values (<0.2), which is expected because these residues interact with the hydrophobic lipid tails of the membrane (Table S14). On the other hand, the ECL and ICL domains have residues with high parch values. Residues D37, K48, K65, D68, E76, R145, E146, D149, K157, and E159 are critical for cis and trans assembly of claudin-5 proteins, and they play key roles in the ion permeability across the BBB. Among the ECL residues, we observed that R81 has a 0.30 parch value, lower than other charged proteins in the ECL domain, which can be attributed to its partial exposure to water and the depth at which R81 sits in line with the lipid headgroups. As expected, the ICL domain residues R116, R191, and D193 lie in the cell’s cytosol and have high parch values (0.64–1.36).

**Figure 12:**
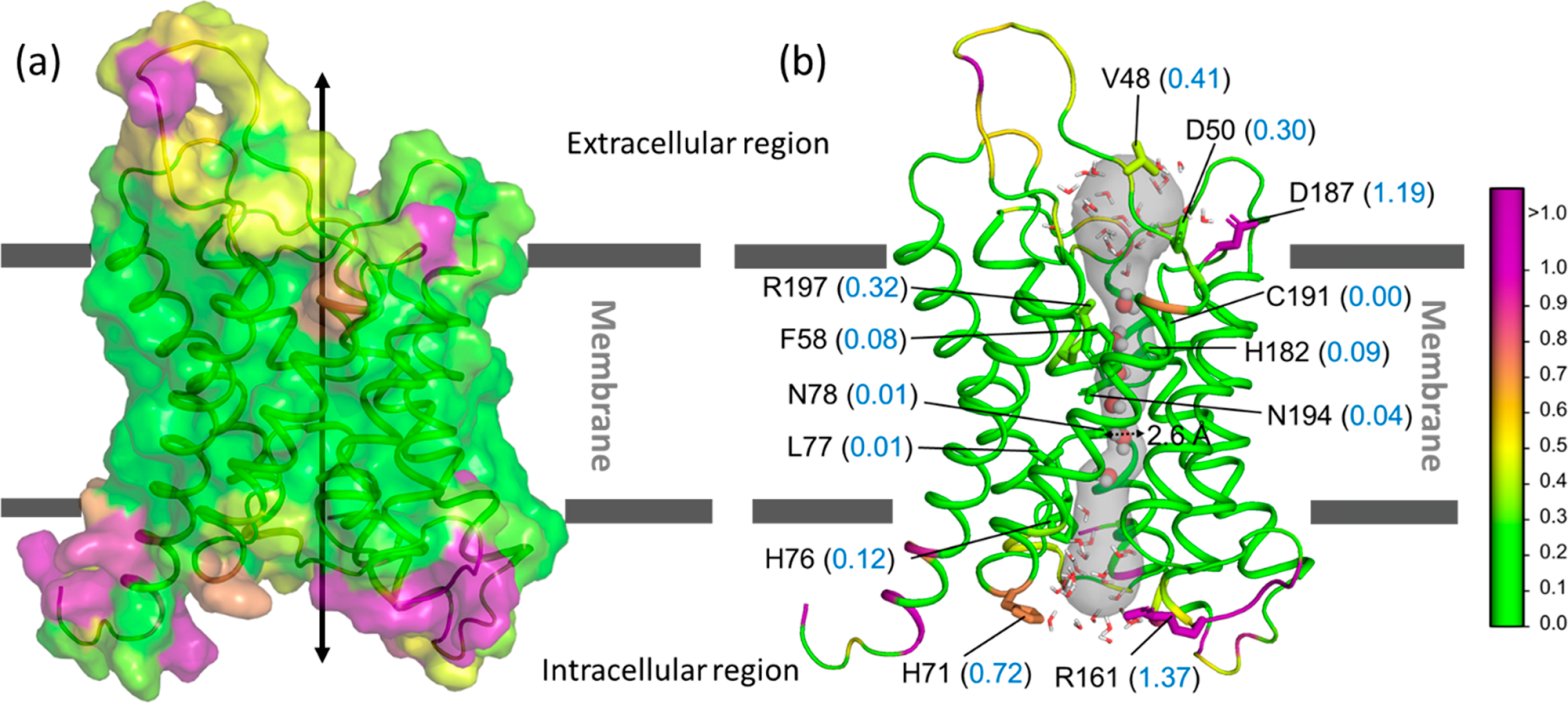
Hydropathy aquaporin1 (AQP1). PDB: 1J4N. (a) Surface representation of the aquaporin protein embedded in the membrane. (b) Ribbon representation showing the water channel along with the water molecules (spheres and sticks; O (red) and H (white)). The channel lining residues are shown along with the parch values (blue font). The residues are colored based on the parch values on the color scale (right).

**Aquaporin1 (AQP1)**, a membrane embedded protein, serves as a water channel in mammals. The protein has extracellular and intracellular domains and a narrow channel that enables water transport across the membrane (Figure 12a). The AQP1 channel reportedly has a hydrophobic character that provides highly selective transport of water.^84,121,122^

The parch analysis shows that the transmembrane helical residues have low parch values (Table S15). The extracellular and intracellular domains have multiple K, R, and D residues with parch values in the 1.2 to 1.9 range. The critical portion of AQP1 is an ∼3 Å wide channel that allows a single thread of water molecules to pass through. Most of the pore lining residues (F, A, L, N, C, G) are uncharged residues and have parch values in the 0–0.09 range corroborating with the well-established observation that the AQP1 channel is hydrophobic (Figure 12b). Within the channel are two positively charged residues, H76 and R197, that have slightly higher parch values of 0.12 and 0.32, respectively. These two residues are critical for the channel’s water selectivity as they act as gatekeepers for proton transfer while having enough charge to allow water to pass in an otherwise hydrophobic pore.^84,121^

**Human ghrelin O-acyltransferase (hGOAT)** is a member of the membrane-bound O-acyl transferase family, which modifies the hormone ghrelin in humans. The structural model of hGOAT was predicted using a combination of coevolutionary constraints and computational modeling with experimental validation via biochemical site-directed mutagenesis and enzyme activity. Although hGOAT has not been crystallized, our model is consistent with its homologue d-alanyl transferase found in bacteria.^80^

**Figure 13:**
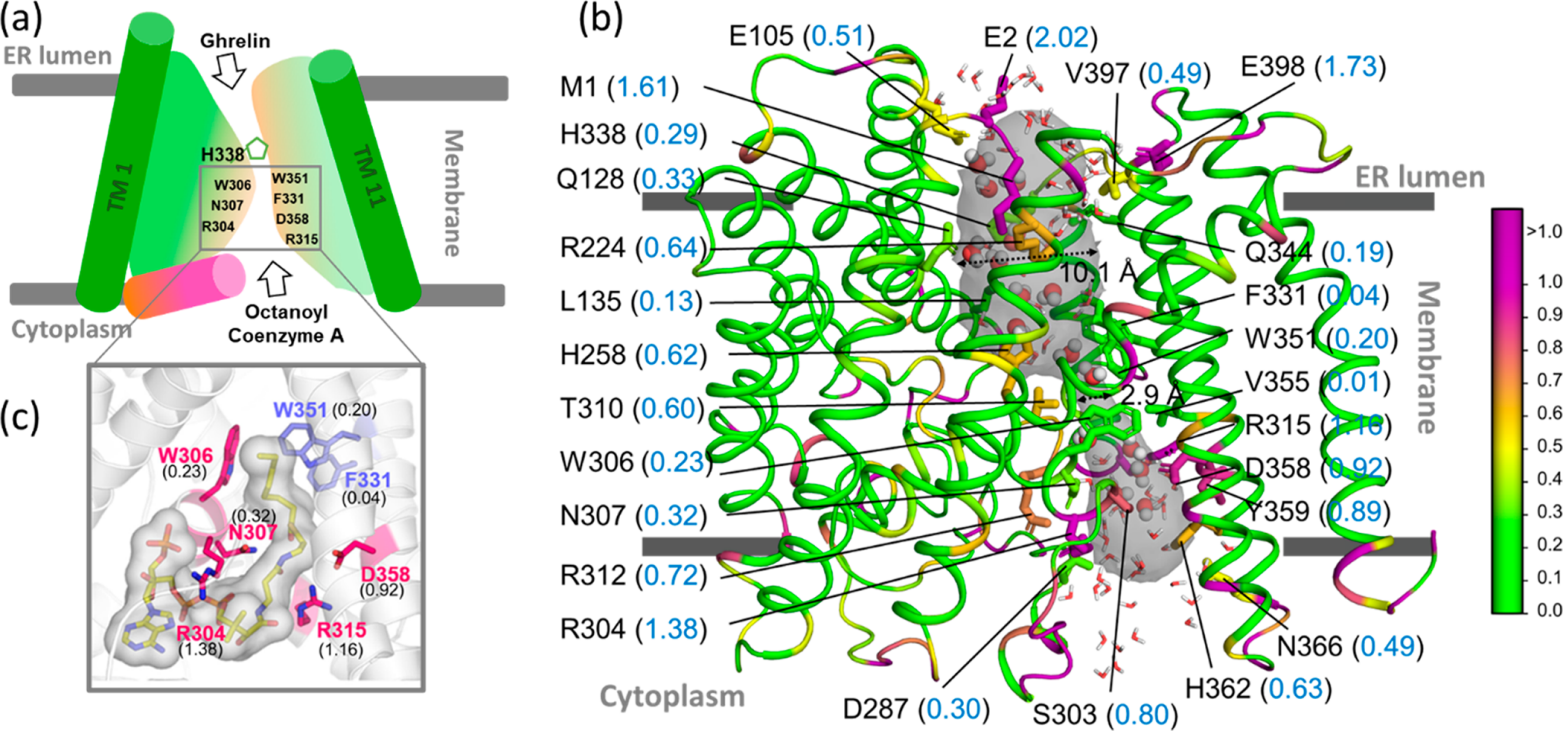
Hydropathy of human ghrelin O-acyltransferase (hGOAT). (a) Schematic of the cone-shaped hGOAT that resides in the ER membrane and contains an internal channel for catalytic transfer of an octanoyl group to ghrelin. For clarity, only cartoon forms of TM1 and TM11 helices (out of 11 transmembrane helices) are shown. Octanoyl coenzyme A binds to hGOAT through the cytoplasmic side, while ghrelin binds through the ER lumen side. The location of the H338 catalytic residue and a few key residues lining the channel are shown. (b) The predicted structure of hGOAT is shown in the ribbon representation. The catalytic channel is shown along with the water molecules (spheres and sticks; O (red) and H (white)). The channel lining residues are shown along with their parch values (blue font, in parentheses). (c) The zoomed-in view of the hGOAT-octanoyl coenzyme A binding pocket. Octanoyl coenzyme A (sticks; C (yellow), O (red), N (blue), and P (orange)) interacts with hGOAT residues F331, W351, W306, R304, N307, R315, and D358. The residues are colored based on the parch values on the color scale (right).

The hGOAT structure has 11 transmembrane helices that bundle together in the shape of a cone (Figure 13a). The enzyme resides in the endoplasmic reticulum (ER) membrane and contains an internal channel that connects the ER lumen to the cytoplasm. The protein’s function is to catalyze the transfer of a fatty acid (n-octanoic acid) to ghrelin (28-amino acid peptide). To facilitate the catalytic reaction, the internal channel is ∼10.1 Å in diameter at its widest (ER lumen side) and ∼2.9 Å in diameter at its narrowest (cytoplasmic side). The narrower cytoplasmic end serves as the binding pocket for octanoyl-coenzyme A, while the wider ER lumen end facilitates the binding of ghrelin. The presumed hGOAT catalytic center, marked by the conserved H338 residue, sits in the center of the channel where the octanoyl group gets added to ghrelin’s S3 residue (Figure 13a).

It was interesting to perform parch analysis on hGOAT because it included the wide water-filled channel in addition to the transmembrane helical bundle. Figure 13b shows the water molecules in the channel and the parch values of the residues lining the channel. The key observations of the parch analysis include the following: (a) the residues lining the channel have higher parch values than the residues in the helices that directly interact with the membrane; (b) the residues at ER lumen vestibule (M1, E2, E398) have high parch values; (c) the parch values are smaller for the residues toward the center of the channel versus the ones at the vestibules; (d) residues proximal to the carbon tail of octanoyl-coenzyme A, such as W351 (0.20), F331 (0.04), W306 (0.23), have low parch values, indicating hydrophobic binding; (e) residues R304 (1.38), N307 (0.32), R315 (1.16), and D358 (0.98) shown to be critical for hGOAT activity and bind to polar and charged groups of octanoyl-coenzyme A have higher parch values, indicating hydrophilic character (Figure 13c). This shows that parch analysis can be performed for interior channels using the same approach as soluble or membrane-embedded proteins. The parch values of all the residues are provided in Table S16.

## Discussion

The parch analysis allows a straightforward way to compare the hydropathy of proteins. We divided the residues of each protein into one of three categories: low (0–0.1), medium (>0.1–0.8), and high (>0.8) parch values. The low to high parch values indicate an increasing affinity for water; however, the three categories do not strictly map to a residue’s hydrophobic, hydroneutral, and hydrophilic character.

**Figure 14:**
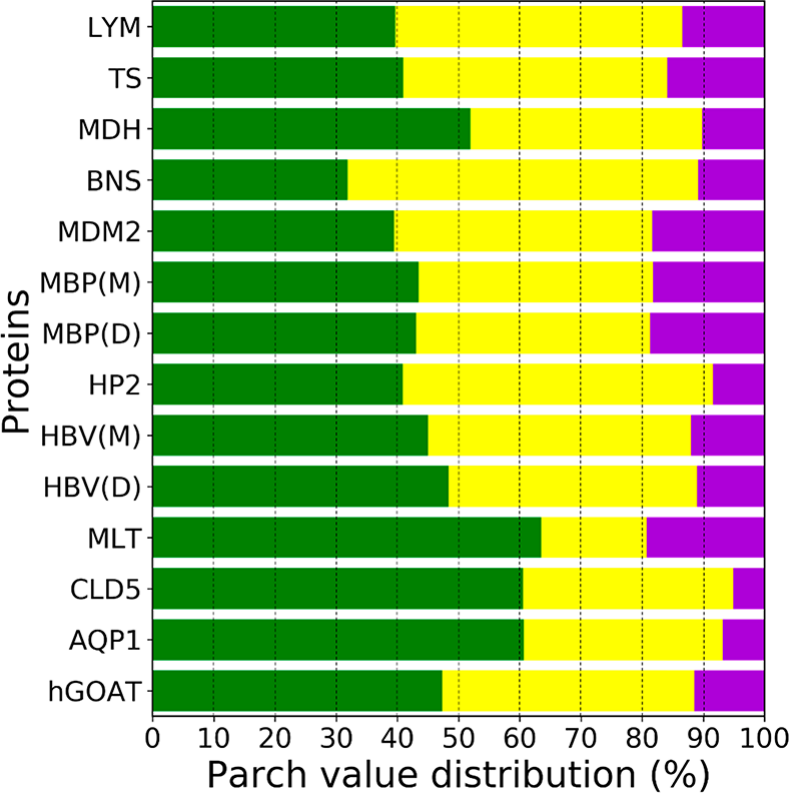
Comparison of the proteins based on the parch value distribution percentages. Residues in each protein were divided into three categories of parch values: low 0–0.1 (green), medium >0.1–0.8 (yellow), and high >0.8 (purple). The plot shows the percent distribution of residues in the three categories (*x*-axis) for the proteins (*y*-axis). The protein monomers and dimers are represented as (M) and (D), respectively. The dashed vertical lines are guides to the eye.

Figure 14 shows the parch value distribution of the representative set of proteins. They key observations from the data are as follows:

- The percentage of low parch value residues is at least 30% in the proteins studied. For a small protein, like barnase, which has all solvent-accessible residues, almost a third of the residues have a low affinity to water. In larger soluble proteins, like MDH, the core residues do not interact with water and have zero parch values.
- The percentage of residues with high parch values is less than 20% in the proteins studied.
- Dimerization of proteins does not alter the percentage distribution of the low, medium, and high parch values for residues.
- Self-associating membrane proteins have a high percentage of residues with low parch values. In membrane proteins Cld5 and AQP1 and the membrane penetrating MLT protein, the majority of low parch value residues lies in the transmembrane domains; therefore, the solvent-exposed residues are fewer. Additionally, these proteins undergo macromolecular assembly in the membrane: AQP1 forms homotetramers or heterotetramers that can further form supramolecular assemblies (junctions and arrays); Cld5 assembles into linear and branched macromolecular strands at the blood-brain interface; and MLT forms high-order assemblies to penetrate the membranes. In contrast, hGOAT has less than 50% of low parch value residues due to the water-filled transmembrane channel, and it is not known to self-assemble. The correlation between the percentage of low parch value residues in the transmembrane domain and self-assembly is an observation from the parch analysis that will need further examination for other membrane proteins.

Until now, we discussed the proteins individually and the significance of the parch values of their residues. We have already established that an amino acid does not have a fixed parch value or a fixed hydropathy. Here, instead of the protein-wise approach, we adopt the residue-wise approach to focus on the 20 amino acid residues and the range of parch values they can exhibit.

**Figure 15:**
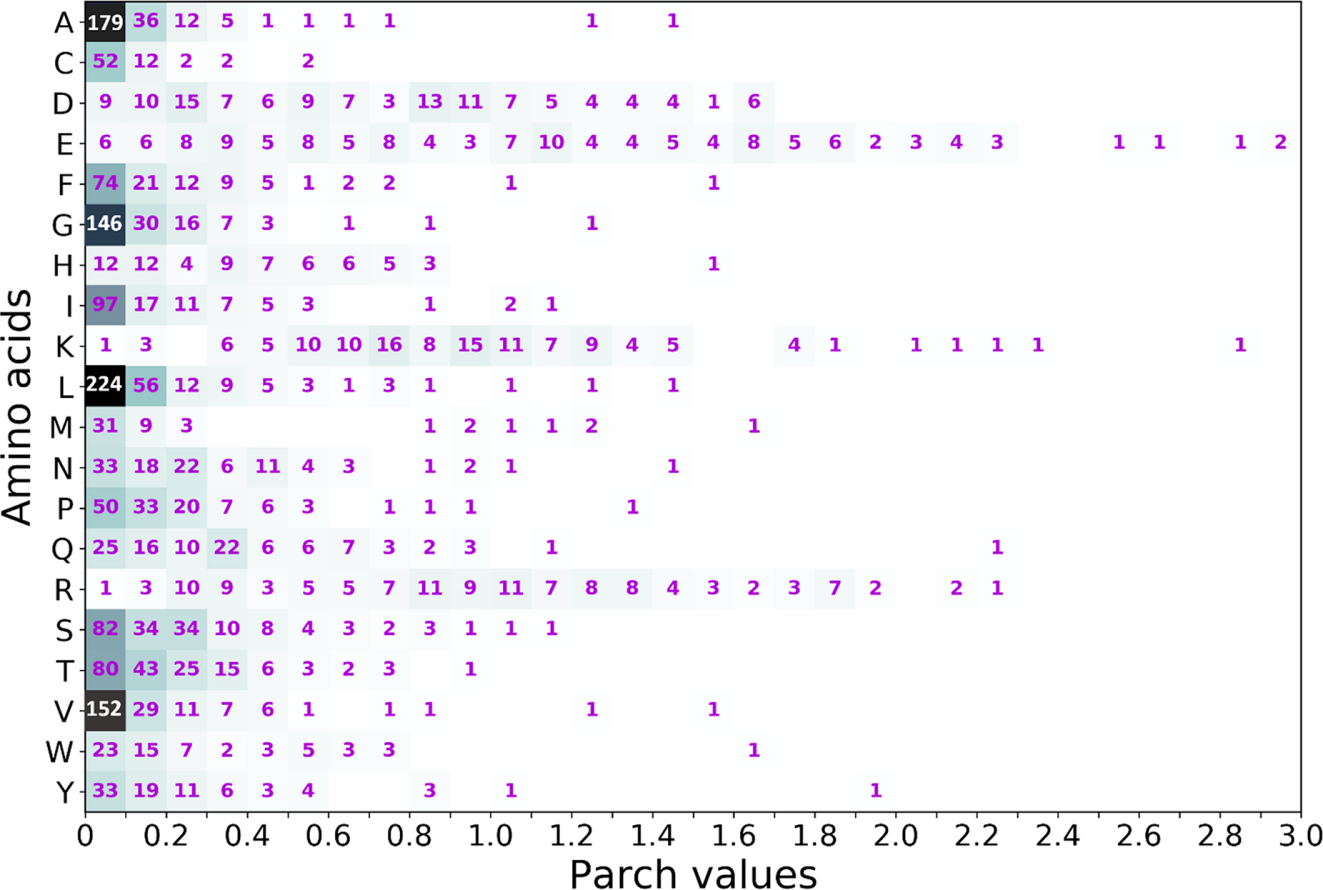
Parch value heat map for the amino acid residues. A total of 2753 total residues from 14 proteins were considered. The residues were binned into a matrix of the 20 amino acids (rows, *y*-axis) and the parch values (columns, *x*-axis) in 0.1 increments from 0 to 3.0. The number of residues in each block is shown.

Taken together, the 14 proteins comprise 2753 residues. We binned the residues in a matrix of the 20 amino acids (rows) and the parch values (columns) in 0.1 increments from 0 to 3.0. The elements of this matrix represent a residue-wise heat map of the parch values (Figure 15). The heat map provides more insights into parch value distribution for the residues. The key observations from the binned data are as follows:

- None of the 20 amino acid residues have a single parch value or a fixed hydropathy.
- A majority of alanine (75%), valine (72%), glycine (71%), leucine (70%), and isoleucine (67%) residues have parch values between 0 and 0.1. Some of these residues can have medium-range parch values and are rarely above 1.0.
- Aspartate, glutamate, lysine, and arginine span a range of parch values. Typically, these charged residues are considered hydrophilic, but we show that they can be hydroneutral or hydrophobic if they are topographically partially or fully buried in the protein’s core.
- Glutamate is the most versatile residue that spans the 0–3.0 parch range.
- Asparagine and glutamine have lower parch values than aspartate and glutamate, respectively.
- Histidine residues can span a 0–0.8 range of parch values.
- Serine (87%), threonine (91%), and proline (89%) have parch values in the 0 to 0.4 range, and they can rarely be above 0.9.
- Aromatic residues tyrosine (78%) and tryptophan (72%) have parch values in the 0 to 0.6 range.
- Most phenylalanine (74%) residues have parch values in the 0 to 0.4 range.
- Cystine (74%) residues have parch values in the 0 to 0.1 range.
- Methionine (61%) residues lie in the 0 to 0.1 parch value range.

The residue-wise binning of the parch values demonstrates that the hydropathy of the 20 amino acids is affected differently by their local environment. The parch scale is a significant advance over the other hydropathy scales that provide a preassigned value for the 20 amino acids. In our experience, the main flaw in the preexisting scales is the assumption that each amino acid residue has a fixed hydropathy.

**Figure 16:**
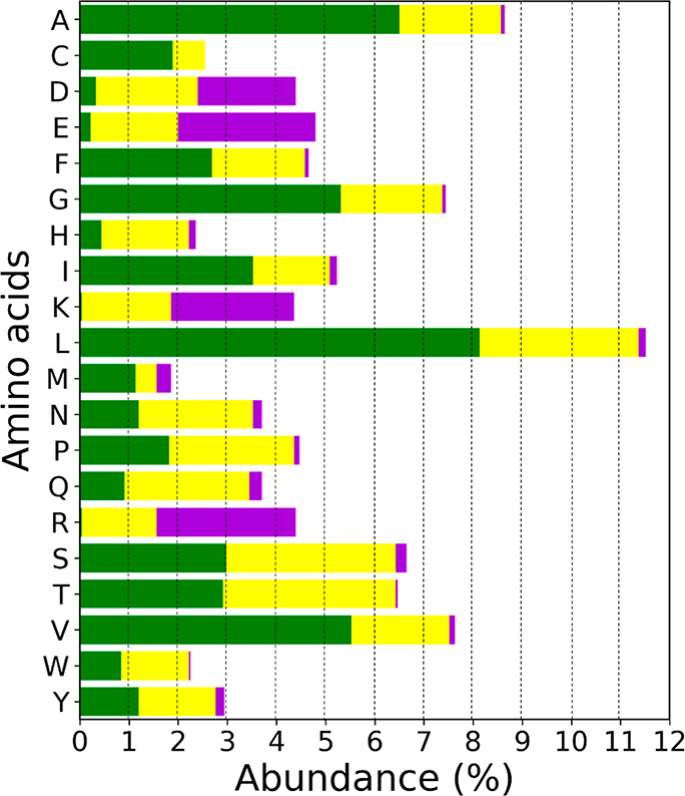
Comparison of the 20 amino acid residues based on the abundance in our data set of 14 proteins (2753 residues). Overlaid on the bar graph of each residue is the proportion of parch values that are categorized as low 0–0.1 (green), medium >0.1–0.8 (yellow), and high >0.8 (purple). The dashed vertical lines are guides to the eye.

Another key aspect to consider in our data set of 14 proteins (or 2753 residues) is the relative abundance of the 20 amino acids. Figure 16 shows the percent abundance of each residue in the data set. Overlaid in the bar graph for each residue is the distribution of low 0–0.1 (green), medium >0.1–0.8 (yellow), and high >0.8 (purple) parch values. In this data set, leucine is the most abundant residue and has the highest percentage (11.5%) of low parch value residues. The successive most abundant residues are alanine (8.6%), valine (7.6%), and glycine (7.4%), which also are major contributors to low parch values. Together these four residues represent 35.1% of residues in the data set. In contrast, methionine (1.9%) is the least abundant residue in our work. Charged residues—aspartic acid, glutamic acid, arginine, and lysine are almost equally abundant, approximately 4.5–4.9%. These residues have a significant proportion of medium and high parch values. Among the uncharged polar residues, serine and threonine are equally abundant at 6.5%, asparagine and glutamine are both 3.7% abundant, and tyrosine is at 3%. These uncharged polar residues have the highest proportion of medium parch values.

In our present work, we realize that the data set is minimal; however, the relative abundance trends and the proportion of parch values within these residues provide fundamental insights into protein biophysics. We are in the process of performing a similar analysis on a large set of proteins and will revisit these initial findings in our future work.

## Conclusions

This work demonstrates that amino acid residues have no fixed hydropathies and should not be simply classified as hydrophilic, hydrophobic, or hydroneutral. We show that residues have variable hydropathies dictated by the nanoscale interactions in their local environment. To quantify such variations, we have developed a new approach called the parch scale that considers the chemical nature and protein structure in computing a residue’s hydropathy. Unlike other hydropathy scales, the parch scale does not assign a fixed value to a residue solely based on its chemical identity. Instead, the parch scale evaluates the collective response of the water molecules in the protein’s first hydration shell to increasing temperatures. The retention or evaporation of hydration water molecules reflects the residue’s chemical and topographical heterogeneity in a protein. Residues that lose water are assigned a lower parch value than the residues that retain water molecules. We studied hydropathies of a representative set of enzymes, immune proteins, integral membrane proteins, and fungal and virus capsid proteins. The parch scale analysis corroborates with the hydropathy data available in the literature for the proteins. We show that a residue’s hydropathy or parch value can be high, low, or zero if it is located on the surface, partially embedded in a pocket, or nestled in the protein’s core, respectively. The workflow of the parch scale analysis has been automated and can readily be used to affordably compute the hydropathy of any protein at the parch scale website.^123^ The parch scale approach will be extended to other classes of molecules in our future work. The parch scale results illustrate how nature uses the 20 amino acid chemical alphabet and three-dimensional geometry to generate millions of proteins with precision for select functions.

## Supporting Information Available

Details on the input parameters and simulation set, parch values of the 20 amino acids, radial distribution functions of water and the proteins, parch values for different simulation parameters, average wall-clock time for the parch calculations for the proteins, and the parch values for all the residues in all proteins (PDF)
