# Supplementary material for: An Affordable Topography-Based Protocol for Assigning a Residue’s Character on a Hydropathy (PARCH) Scale
Source: J Chem Theory Comput. 2023 Apr 5;20(4):1656–72. doi: 10.1021/acs.jctc.3c00106 (PMC10902853; doi:10.1021/acs.jctc.3c00106)
Supplement: Supplementary file 1 — ct3c00106_si_001.pdf [file ct3c00106_si_001.pdf]

Supporting Information

An Affordable Topography-based Protocol for Assigning Residue Character on Hydropathy (PARCH) scale

Jingjing Ji,[1] Britnie Carpentier,[1] Arindam Chakraborty,[2*] and Shikha Nangia[1*]

[1]*Department of Biomedical and Chemical Engineering, Syracuse University, Syracuse, NY 13244, USA*

[2]*Department of Chemistry, Syracuse University, Syracuse, NY 13244, USA*

*Address for correspondence:
Dr. Shikha Nangia
343 Link Hall
Department of Biomedical and Chemical Engineering
Syracuse University, Syracuse, NY 13244, USA
Phone (315) 443 0571 | Email: snangia@syr.edu
ORCID 0000-0003-1170-8461

Dr. Arindam Chakraborty
Department of Chemistry
4-008 Center for Science and Technology (CST)
Syracuse University, Syracuse, NY 13244, USA
Phone (315) 443 5803 | Email: archakra@syr.edu
ORCID 0000-0003-2710-0637

Contents

Table S1. Input parameter definitions and setup 3

Table S2. PARCH values of the 20 single amino acids. 4

Figure S1. Radial distribution function of water and proteins 5

Figure S2. Parch values of (a) BNS and (b) TS residues for different d_{ion} and d_b values. 6

Figure S3. Average wall-clock time for parch scale calculations for (a) BNS and (b) TS using different d_{ion} and d_b values. 7

Figure S4. Parch values of (a) BNS and (b) TS residues for different d_{water} values 9

Figure S5. Parch values of (a) BNS and (b) TS residues using different annealing rates 10

Figure S6. Average wall-clock time for parch scale calculations for (a) BNS and (b) TS using different annealing rates 11

Figure S7. Parch values of the (a) BNS and (b) TS residues for different force constants 13

Figure S8. Average wall-clock time for the PARCH scale calculations 14

Table S3. PARCH values of LYM residues 15

Table S4. PARCH values of TS residues 17

Table S5. PARCH values of MDH residues 20

Table S6. PARCH values of BNS residues 23

Table S7. PARCH values of MDM2 residues 24

Table S8. PARCH values of MBP(M) residues 25

Table S9. PARCH values of MBP(D) residues 26

Table S10. PARCH values of HP2 residues 28

Table S11. PARCH values of HBV(M) residues 29

Table S12. PARCH values of HBV(D) residues 31

Table S13. PARCH values of MLT residues 34

Table S14. PARCH values of CLD5 residues 35

Table S15. PARCH values of AQP1 residues 37

Table S16. PARCH values of hGOAT residues 40

Table S1. **Input parameter definitions and setup**

Parameter	Value	Tested values	Definition
d_{max}	Depends on the size of the protein	Not applicable	Maximum distance between the center of geometry (COG) of the protein and COG of each amino acid residue in the protein
d_{ion}	3.0 nm	3.0 nm<br>3.5 nm<br>4.0 nm<br>4.5 nm<br>5.0 nm	Radial distance between the surface of the protein (d_{max}) and location of the counterions
$d_{interion}$	4.0 nm	Not applicable	Minimum distance between any two counterions
d_b	3.0 nm	3.0 nm<br>3.5 nm<br>4.0 nm<br>4.5 nm<br>5.0 nm	Distance between the counterion and the simulation box boundary
d_{shell}	4.15 Å	3.15 Å<br>4.15 Å<br>5.15 Å	Thickness of water layer when a protein in solvated for parch calculations
l	$2\times(d_{max}+d_{ion}+d_b)$	Not applicable	Edge length of the simulation box
d_{water}	3.15 Å	3.15 Å<br>3.25 Å	Cut-off used to compute the number of waters around an amino acid residue

Table S2. PARCH values of the 20 single amino acids.

The N- and C-termini of the single amino were charged as well as the side chains (when applicable).

Rank	Name	PARCH value
1	Ile	3.25
2	Val	3.40
3	Cys	4.11
4	Phe	4.45
5	Pro	4.55
6	Ala	4.55
7	Thr	4.61
8	Leu	4.71
9	Met	4.85
10	Trp	5.09
11	Asn	5.28
12	Gln	5.50
13	Gly	5.62
14	Glu	6.11
15	Tyr	6.18
16	Ser	6.34
17	Arg	6.43
18	His	6.46
19	Asp	8.24
20	Lys	10.00

Figure S1. Radial distribution function of water and proteins

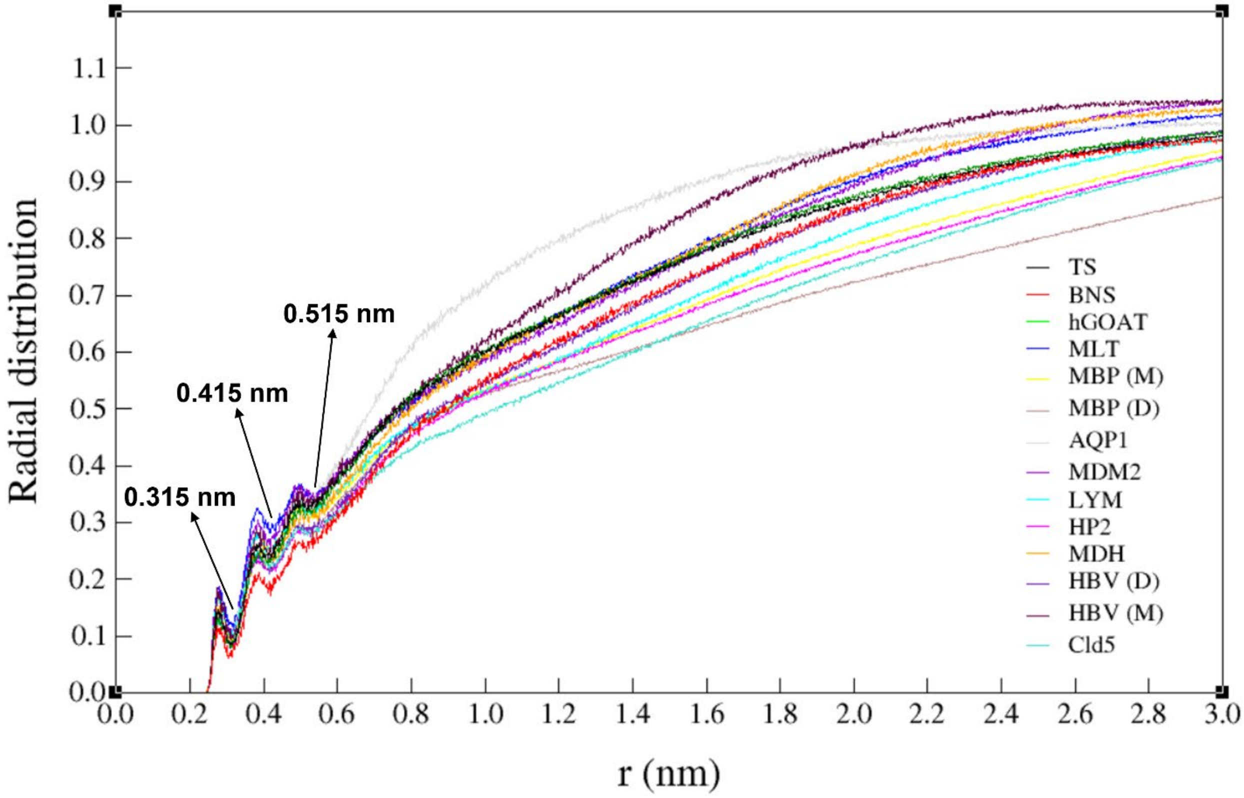

We evaluated the hydration of the equilibrated proteins. Figure S1 shows the radial distribution functions of the oxygen atom in water (OH2) relative to the protein backbone for the proteins. In all cases, water was structured around the proteins in the first, second, and third hydration shells at an average distance of 0.315 nm, 0.415 nm, and 0.515 nm, respectively. The d_{shell} = 0.415 nm was selected.

Figure S2. Parch values of (a) BNS and (b) TS residues for different d_{ion} and d_b values.

(a)

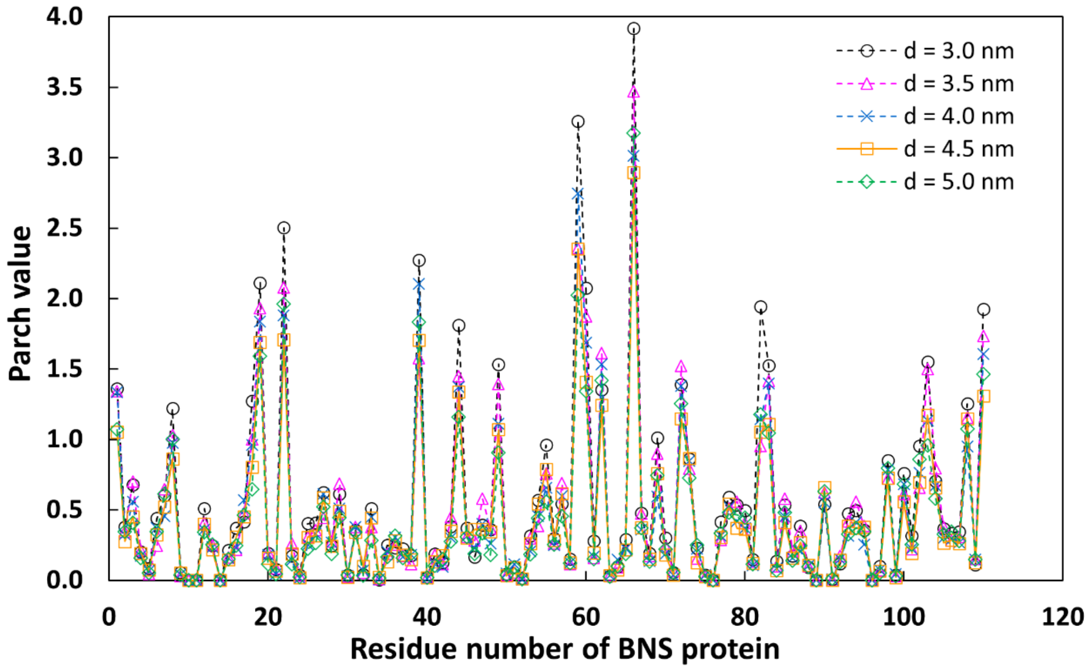

(b)

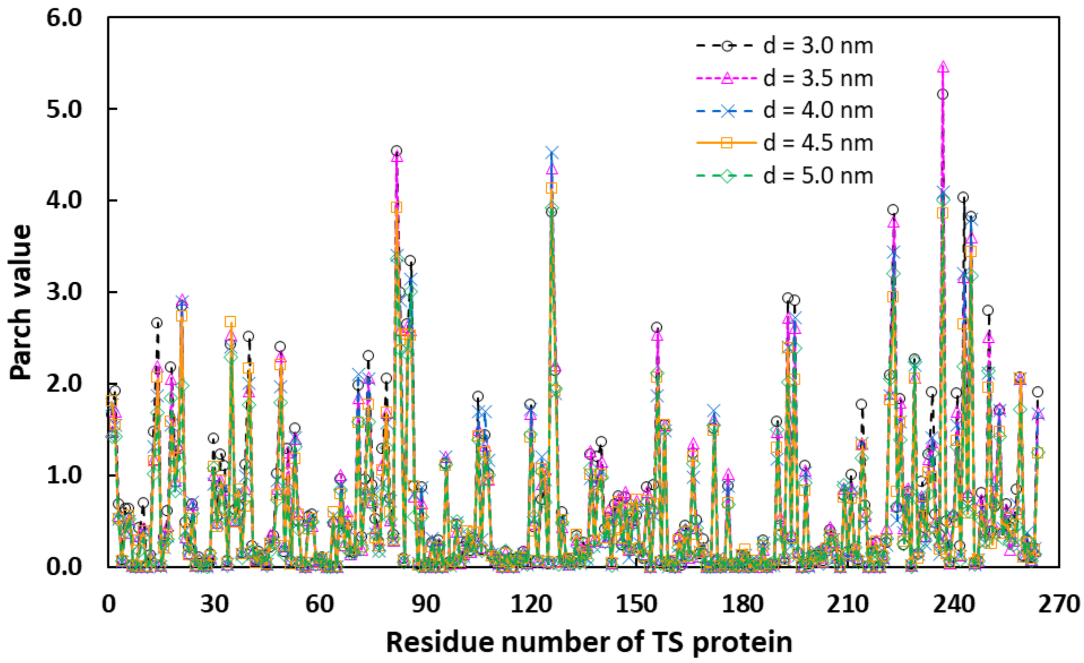

Figure S3. Average wall-clock time for parch scale calculations for (a) BNS and (b) TS using different d_{ion} and d_b values.

(a)

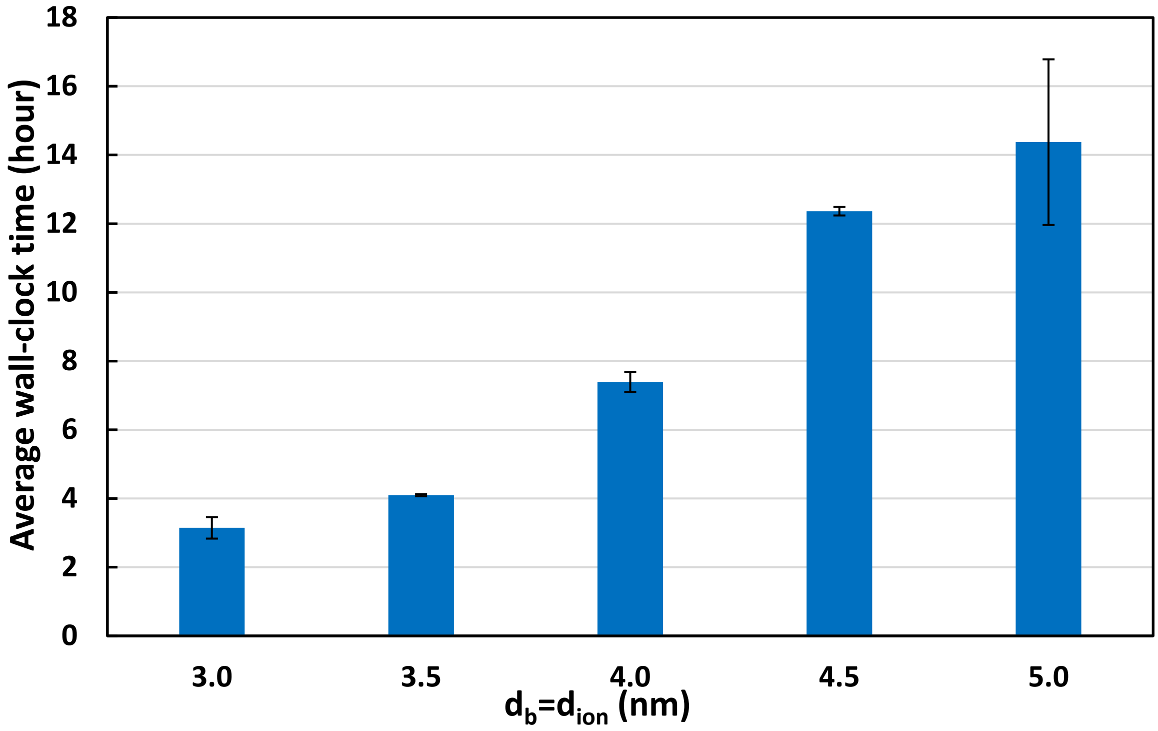

(b)

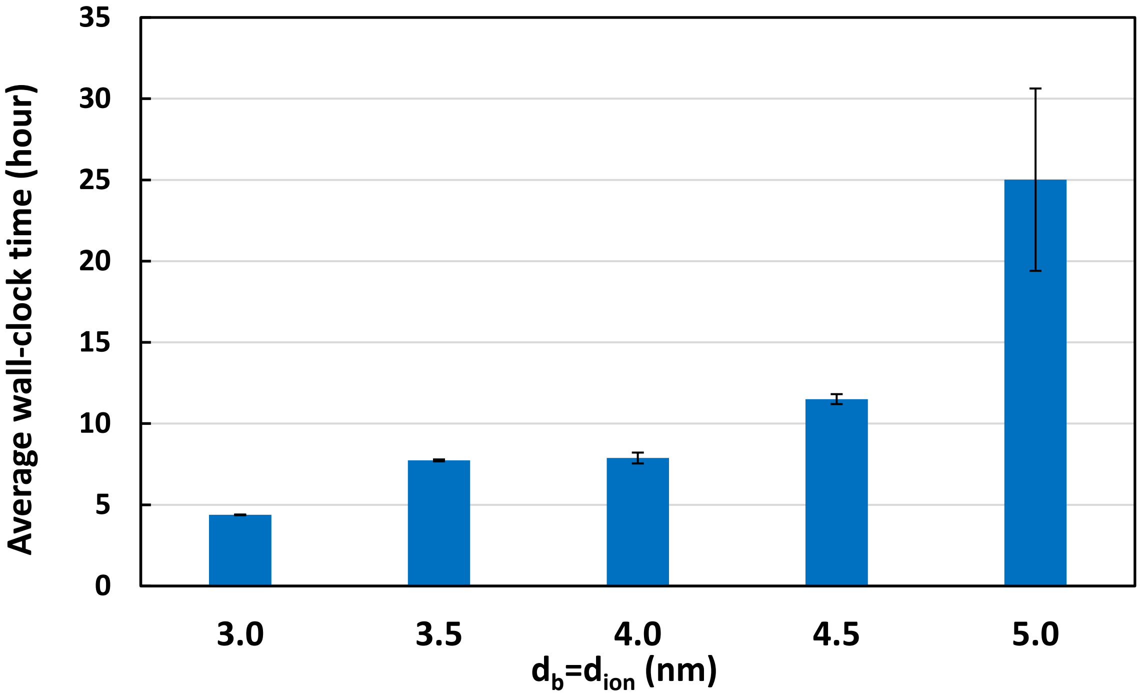

We evaluated the effect of d_{ion} and d_b on the parch scale calculations using BNS and TS proteins as test cases. An increase in the d_{ion} and d_b values from 3.0 to 5.0 nm affected the parch values, but the changes were small (Figure S2). The cutoff did not change the parch value trends; the residues with high parch values remained higher than others when d_{ion} and d_b values were changed.

In terms of the computational cost, increasing d_{ion} and d_b values had a substantial effect (Figure S3). For example, the wall-clock time for BNS at $d_{ion} = d_b = 5.0$ nm was 4x larger than $d_b = d_{ion} = 3.0$, and for TS, the exact change led to a 6x higher cost. We expect that for larger proteins than BNS and TS, the computational cost will be much larger with $d_b = d_{ion} = 5.0$ nm for a small change in the residues' parch values. To ensure the affordability of the PARCH scale calculations, we selected $d_b = d_{ion} = 3.0$ nm.

Figure S4. Parch values of (a) BNS and (b) TS residues for different d_{water} values

(a)

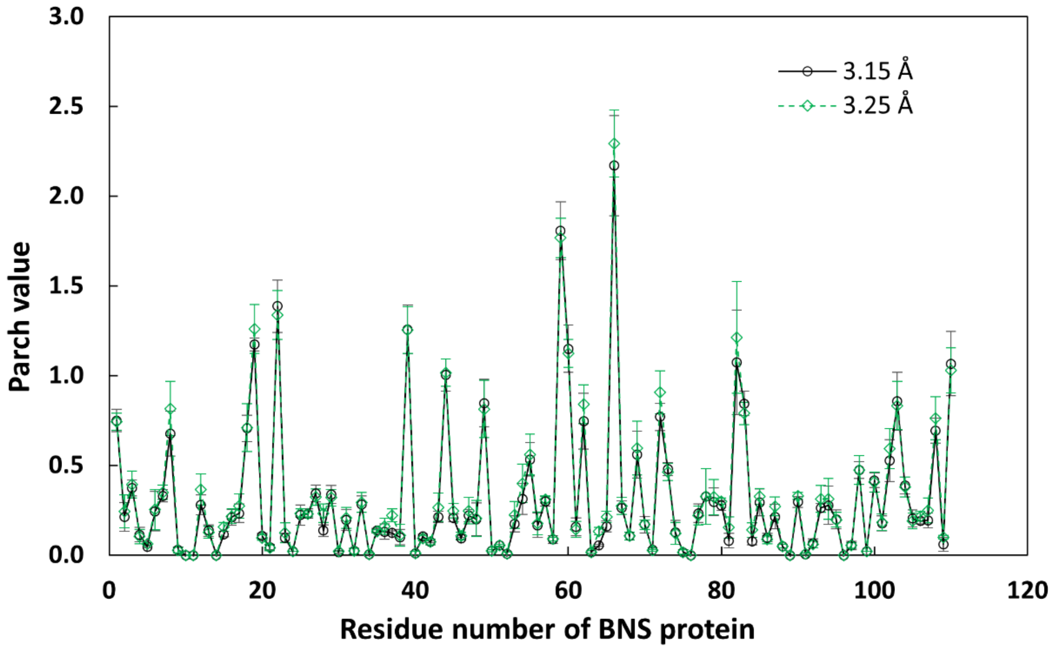

(b)

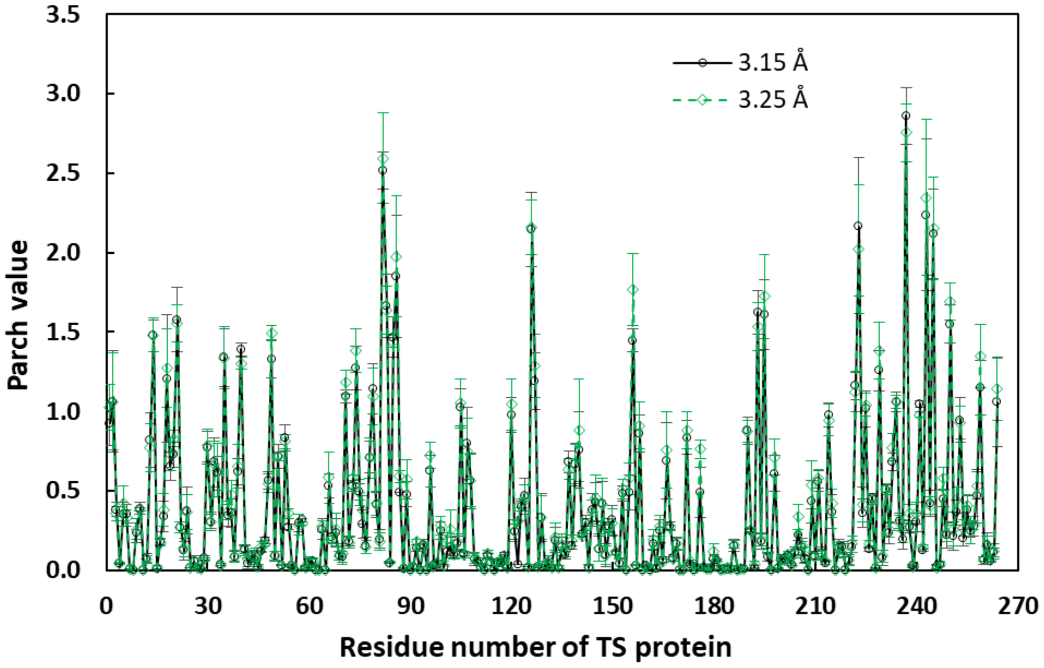

Figure S5. Parch values of (a) BNS and (b) TS residues using different annealing rates

(a)

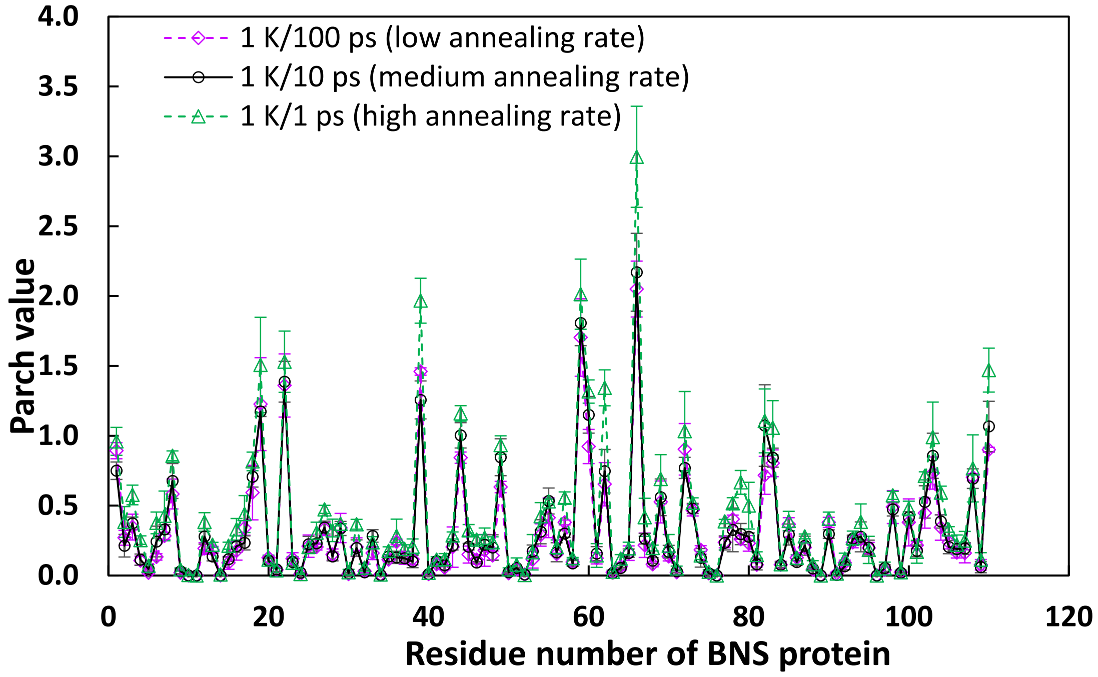

(b)

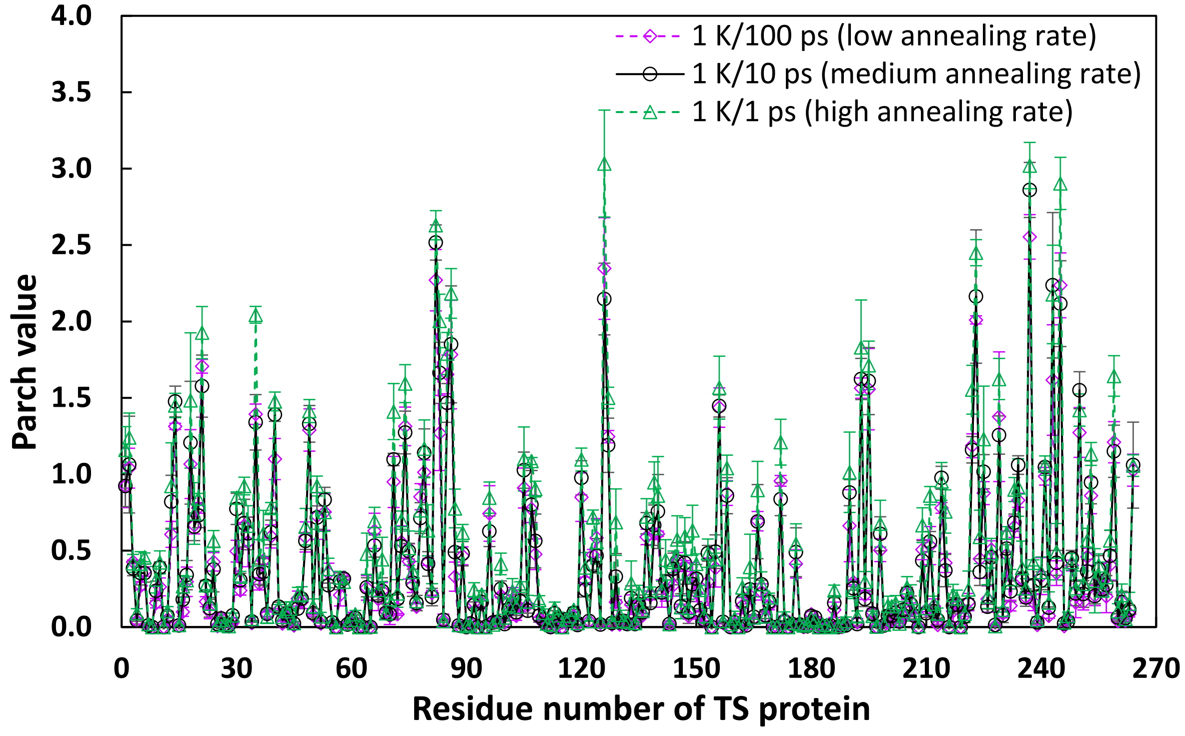

Figure S6. Average wall-clock time for parch scale calculations for (a) BNS and (b) TS using different annealing rates

(a)

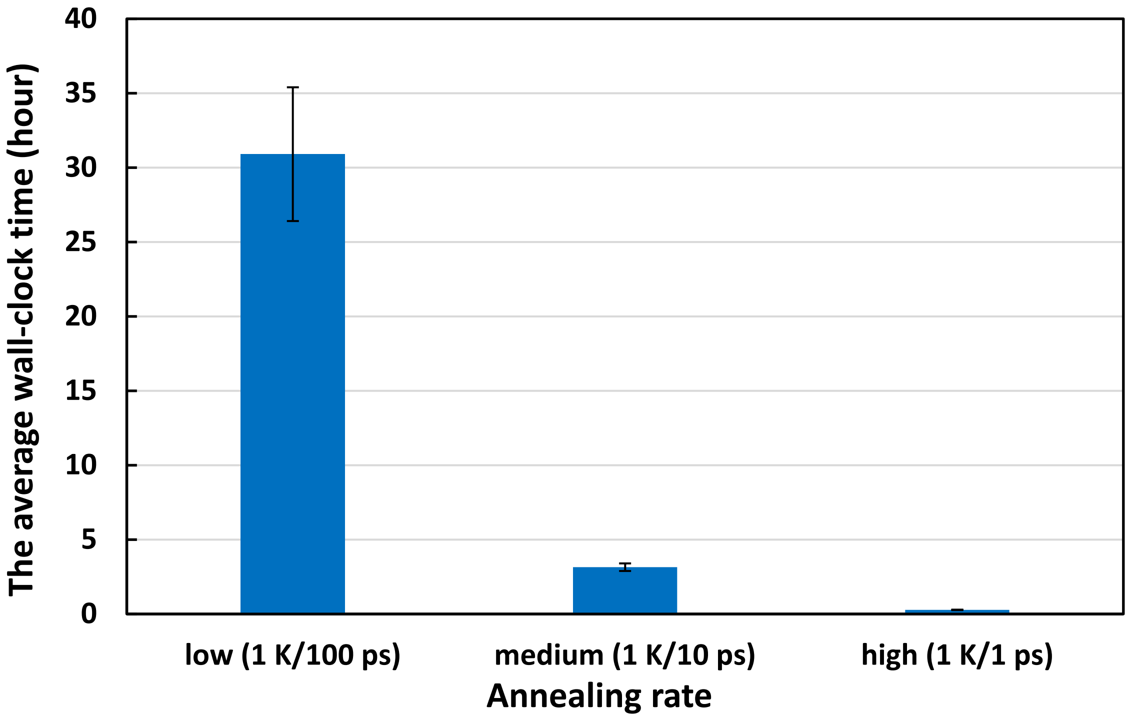

(b)

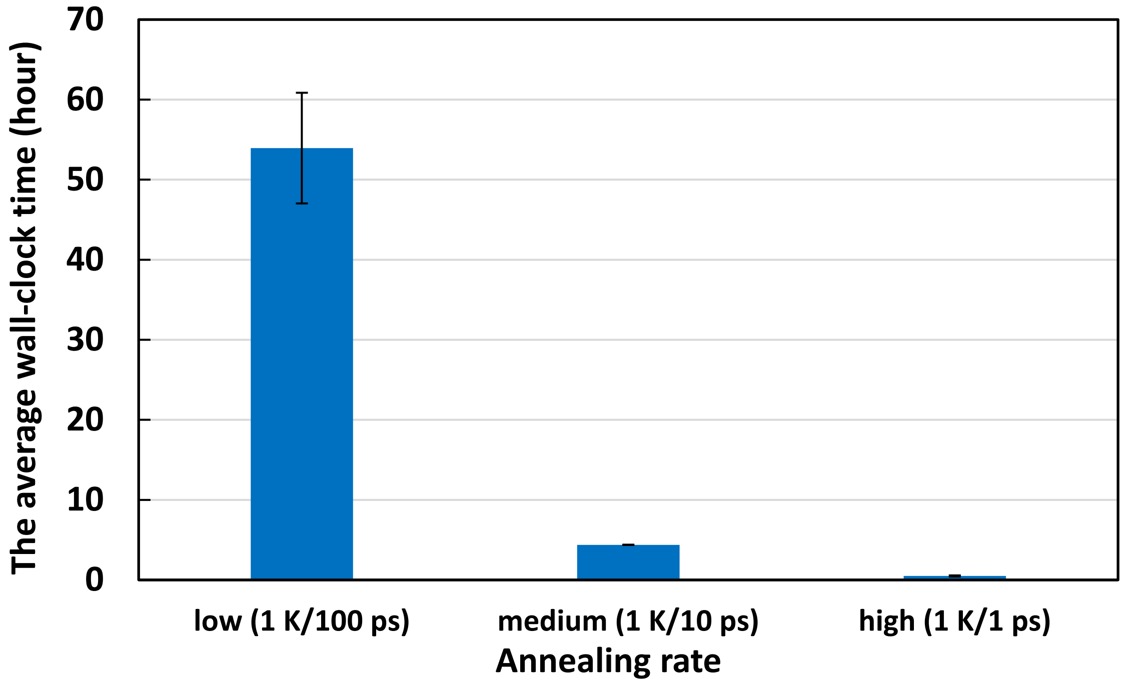

We studied the effect of annealing rates on parch value calculations for BNS and TS as test cases. For both proteins, three annealing rates—1 K/1 ps (high), 1 K/10 ps (medium), and 1 K/100 ps (low)—were investigated. We found that high annealing rates led to higher parch values than medium and low annealing rates (Figure S4). A view into the annealing simulation trajectories showed that water could not establish a network around the protein at a high annealing rate, leading to an overestimation of the parch values. In contrast, water can establish a network at medium and low annealing rates. Also, the parch value differences between medium and low annealing rates are minor.

The computational costs for the three annealing rates are remarkably different (Figure S5). Although the high annealing rate is the least expensive, it is unfit due to overestimating the parch values. The cost of the low annealing rate is 6 to 9 times higher than the medium annealing rate. Therefore, based on accuracy and efficiency, we selected the medium annealing rate (1 K/10 ps) for our parch calculations.

Figure S7. PARCH values of the (a) BNS and (b) TS residues for different force constants

(a)

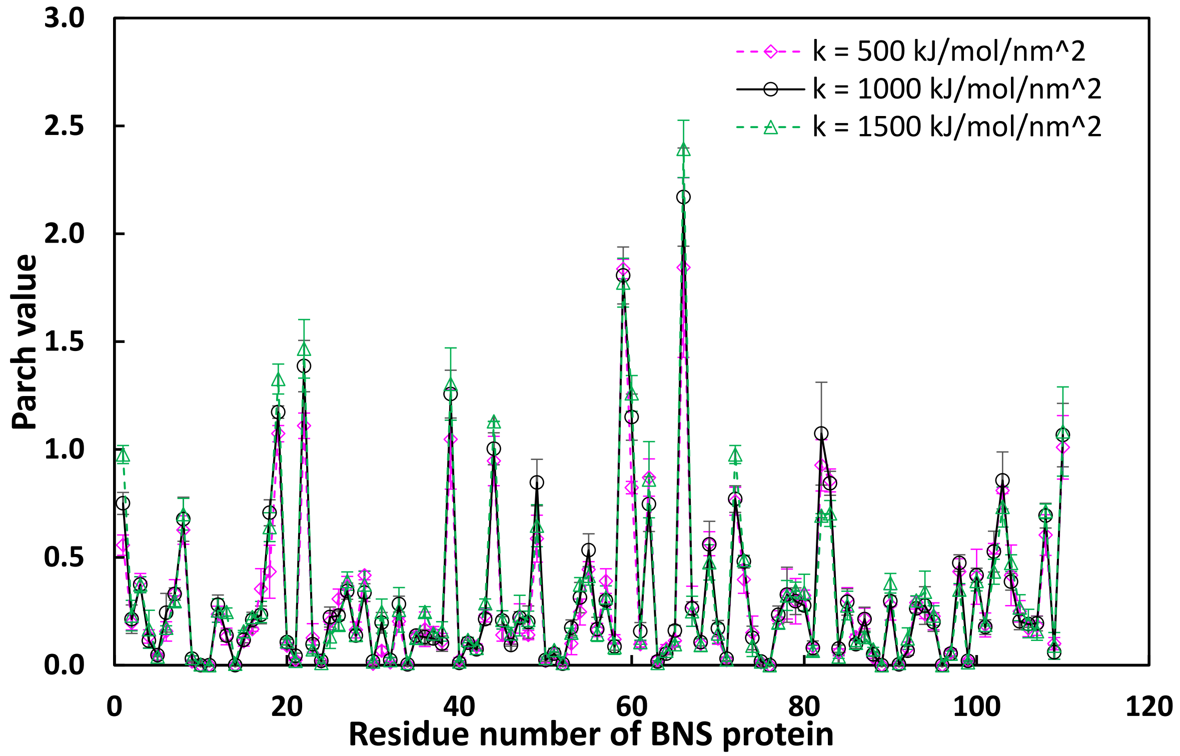

(b)

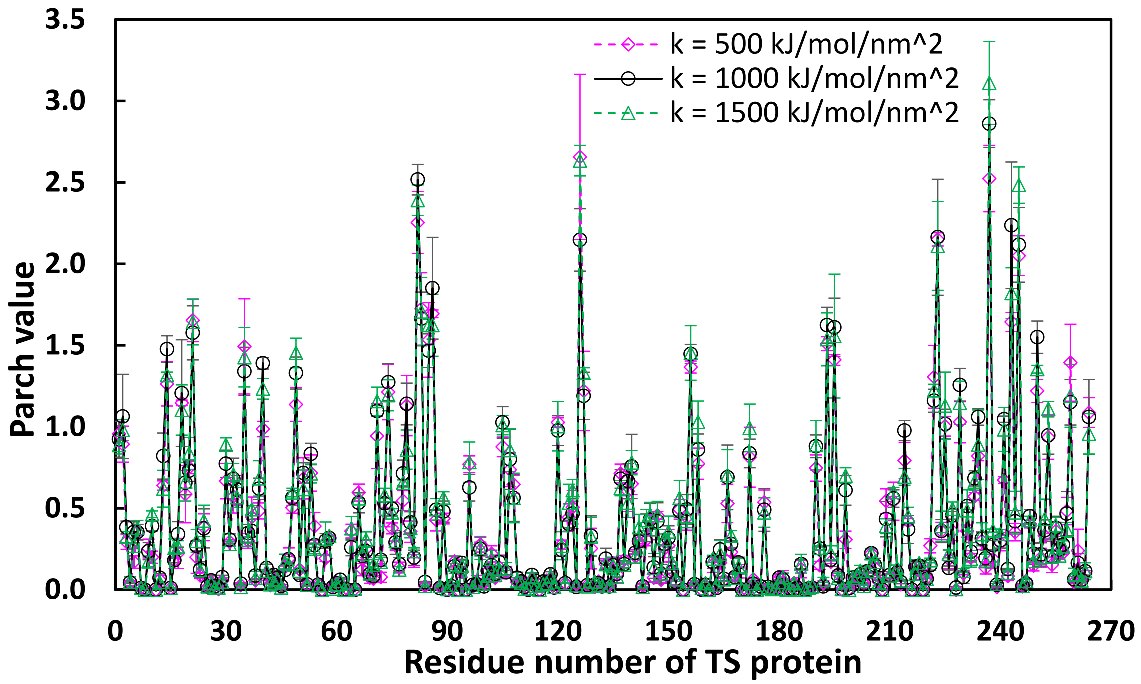

Figure S8. Average wall-clock time for the PARCH scale calculations

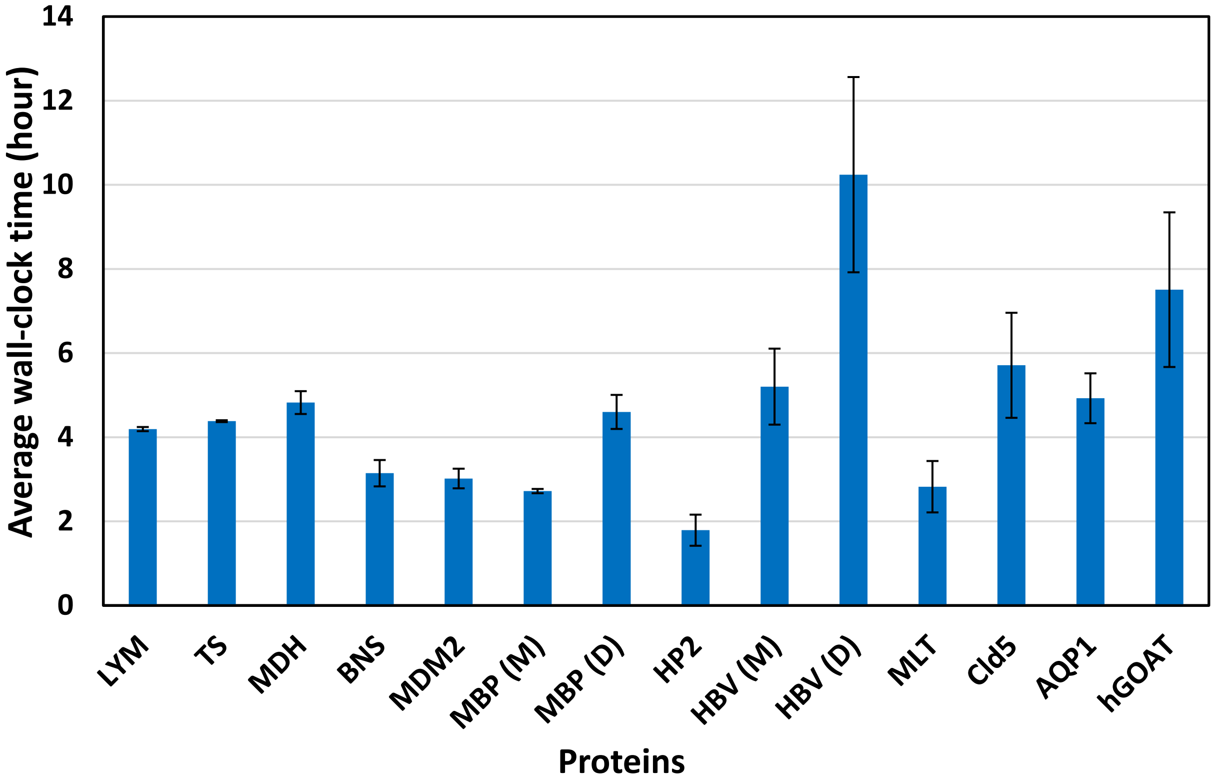

Table S3. PARCH values of LYM residues

#	Name	Parch value	
		avg	std
1	M	1.13	0.21
2	N	0.28	0.11
3	I	0.04	0.00
4	F	0.35	0.14
5	E	0.70	0.10
6	M	0.01	0.01
7	L	0.00	0.00
8	R	0.84	0.02
9	I	0.36	0.10
10	D	0.11	0.02
11	E	0.72	0.14
12	G	0.06	0.05
13	L	0.17	0.02
14	R	1.05	0.30
15	L	0.21	0.04
16	K	1.12	0.04
17	I	0.05	0.01
18	Y	0.43	0.10
19	K	1.03	0.11
20	A	0.23	0.06
21	T	0.72	0.11
22	E	1.26	0.13
23	G	0.16	0.05
24	Y	0.58	0.08
25	Y	0.11	0.03
26	T	0.02	0.01
27	I	0.00	0.00
28	G	0.01	0.00
29	I	0.00	0.00
30	G	0.13	0.02
31	H	0.19	0.00
32	L	0.40	0.04
33	L	0.03	0.02
34	T	0.19	0.02
35	K	1.08	0.09
36	S	0.08	0.02
37	P	0.17	0.05
38	S	0.12	0.06
39	L	0.11	0.05
40	N	0.13	0.03
41	A	0.06	0.05
42	A	0.01	0.01
43	K	0.89	0.20
44	S	0.04	0.01
45	E	0.31	0.06
46	L	0.00	0.00
47	D	0.18	0.05
48	K	0.78	0.23
49	A	0.30	0.02
50	I	0.12	0.01
51	G	0.05	0.03
52	R	0.34	0.02
53	N	0.23	0.01
54	T	0.08	0.02
55	N	0.62	0.06
56	G	0.02	0.02
57	V	0.08	0.03
58	I	0.00	0.00
59	T	0.16	0.05
60	K	0.69	0.13
61	D	0.39	0.05
62	E	0.05	0.01
63	A	0.00	0.00
64	E	0.48	0.10
65	K	0.60	0.10
66	L	0.02	0.01
67	F	0.17	0.06
68	N	0.28	0.07
69	Q	0.66	0.19
70	D	0.26	0.01
71	V	0.01	0.01
72	D	0.94	0.08
73	A	0.04	0.00
74	A	0.01	0.01
75	V	0.09	0.05
76	R	1.88	0.26
77	G	0.03	0.02
78	I	0.05	0.01
79	L	0.13	0.08
80	R	1.05	0.13
81	N	0.06	0.02
82	A	0.15	0.04
83	K	0.86	0.08
84	L	0.01	0.01
85	K	0.34	0.02
86	P	0.06	0.02
87	V	0.01	0.01
88	Y	0.10	0.02
89	D	0.24	0.03
90	S	0.14	0.01
91	L	0.04	0.00
92	D	0.47	0.02
93	A	0.16	0.06
94	V	0.14	0.04
95	R	0.21	0.04
96	R	0.32	0.03
97	A	0.02	0.01
98	A	0.00	0.00
99	L	0.02	0.02
100	I	0.03	0.01
101	N	0.00	0.00
102	M	0.04	0.04
103	V	0.12	0.03
104	F	0.17	0.02
105	Q	0.31	0.04
106	M	0.08	0.04
107	G	0.09	0.06
108	E	0.40	0.06
109	T	0.21	0.03
110	G	0.02	0.01
111	V	0.01	0.01
112	A	0.01	0.01
113	G	0.05	0.02
114	F	0.10	0.03
115	T	0.20	0.04
116	N	0.32	0.10
117	S	0.01	0.00
118	L	0.06	0.02
119	R	1.06	0.24
120	M	0.03	0.02

PARCH values of LYM residues (continued)

#	Name	Parch value	
		avg	std
121	L	0.18	0.08
122	Q	0.34	0.04
123	Q	0.31	0.07
124	K	0.81	0.22
125	R	1.14	0.20
126	W	0.54	0.19
127	D	0.82	0.10
128	E	0.40	0.10
129	A	0.03	0.02
130	A	0.04	0.03
131	V	0.25	0.15
132	N	0.13	0.04
133	L	0.07	0.01
134	A	0.10	0.01
135	K	0.97	0.05
136	S	0.05	0.03
137	R	0.94	0.14
138	W	0.03	0.01
139	Y	0.22	0.09
140	N	0.24	0.10
141	Q	0.61	0.12
142	T	0.19	0.03
143	P	0.18	0.03
144	N	0.54	0.10
145	R	0.23	0.05
146	A	0.00	0.00
147	K	1.13	0.10
148	R	0.32	0.10
149	V	0.00	0.00
150	I	0.00	0.00

#	Name	Parch value	
		avg	std
151	T	0.01	0.01
152	T	0.01	0.00
153	F	0.09	0.03
154	R	1.01	0.10
155	T	0.15	0.06
156	G	0.02	0.02
157	T	0.20	0.05
158	W	0.37	0.07
159	D	0.66	0.07
160	A	0.07	0.02
161	Y	0.01	0.00
162	K	1.32	0.27
163	N	0.40	0.09
164	L	1.48	0.06

Table S4. PARCH values of TS residues

#	Name	Parch value	
		avg	std
1	M	0.92	0.14
2	K	1.06	0.32
3	Q	0.38	0.05
4	Y	0.04	0.01
5	L	0.35	0.08
6	E	0.35	0.06
7	L	0.01	0.00
8	M	0.00	0.00
9	Q	0.24	0.06
10	K	0.39	0.04
11	V	0.00	0.00
12	L	0.07	0.03
13	D	0.82	0.17
14	E	1.48	0.10
15	G	0.01	0.00
16	T	0.18	0.02
17	Q	0.34	0.10
18	K	1.21	0.40
19	N	0.66	0.09
20	D	0.73	0.08
21	R	1.58	0.20
22	T	0.27	0.04
23	G	0.12	0.06
24	T	0.38	0.15
25	G	0.02	0.00
26	T	0.06	0.04
27	L	0.02	0.02
28	S	0.01	0.00
29	I	0.08	0.02
30	F	0.77	0.10

#	Name	Parch value	
		avg	std
31	G	0.31	0.05
32	H	0.68	0.16
33	Q	0.61	0.11
34	M	0.04	0.01
35	R	1.34	0.18
36	F	0.35	0.07
37	N	0.36	0.09
38	L	0.09	0.03
39	Q	0.62	0.08
40	D	1.39	0.04
41	G	0.13	0.03
42	F	0.04	0.04
43	P	0.09	0.03
44	L	0.07	0.05
45	V	0.02	0.01
46	T	0.12	0.00
47	T	0.19	0.02
48	K	0.56	0.06
49	R	1.33	0.12
50	C	0.09	0.03
51	H	0.72	0.08
52	L	0.03	0.01
53	R	0.83	0.08
54	S	0.27	0.02
55	I	0.03	0.01
56	I	0.00	0.00
57	H	0.30	0.06
58	E	0.32	0.01
59	L	0.02	0.01
60	L	0.04	0.03

#	Name	Parch value	
		avg	std
61	W	0.06	0.02
62	F	0.00	0.00
63	L	0.00	0.00
64	Q	0.26	0.06
65	G	0.00	0.00
66	D	0.53	0.08
67	T	0.21	0.03
68	N	0.24	0.09
69	I	0.09	0.03
70	A	0.08	0.04
71	Y	1.10	0.04
72	L	0.18	0.03
73	H	0.53	0.04
74	E	1.27	0.14
75	N	0.50	0.05
76	N	0.29	0.09
77	V	0.16	0.05
78	T	0.71	0.10
79	I	1.14	0.16
80	W	0.41	0.08
81	D	0.20	0.06
82	E	2.52	0.12
83	W	1.66	0.20
84	A	0.05	0.01
85	D	1.47	0.13
86	E	1.85	0.38
87	N	0.49	0.00
88	G	0.01	0.00
89	D	0.48	0.08
90	L	0.00	0.00

#	Name	Parch value	
		avg	std
91	G	0.02	0.01
92	P	0.14	0.02
93	V	0.00	0.00
94	Y	0.16	0.03
95	G	0.00	0.00
96	K	0.63	0.10
97	Q	0.03	0.01
98	W	0.03	0.03
99	R	0.25	0.05
100	A	0.02	0.02
101	W	0.12	0.04
102	P	0.15	0.06
103	T	0.10	0.02
104	P	0.17	0.05
105	D	1.03	0.12
106	G	0.11	0.02
107	R	0.80	0.23
108	H	0.56	0.18
109	I	0.07	0.04
110	D	0.05	0.02
111	Q	0.02	0.01
112	I	0.00	0.00
113	T	0.09	0.02
114	T	0.05	0.02
115	V	0.00	0.00
116	L	0.05	0.02
117	N	0.04	0.01
118	Q	0.09	0.02
119	L	0.01	0.01
120	K	0.98	0.11

PARCH values of TS residues (continued)

#	Name	Parch value	
		avg	std
121	N	0.24	0.05
122	D	0.04	0.00
123	P	0.40	0.03
124	D	0.47	0.11
125	S	0.02	0.01
126	R	2.15	0.23
127	R	1.19	0.18
128	I	0.03	0.01
129	I	0.33	0.15
130	V	0.03	0.01
131	S	0.04	0.03
132	A	0.02	0.01
133	W	0.19	0.08
134	N	0.02	0.01
135	V	0.15	0.06
136	G	0.10	0.03
137	E	0.68	0.07
138	L	0.16	0.03
139	D	0.67	0.13
140	K	0.76	0.24
141	M	0.23	0.01
142	A	0.30	0.05
143	L	0.02	0.02
144	A	0.38	0.10
145	P	0.43	0.12
146	C	0.14	0.08
147	H	0.42	0.14
148	A	0.10	0.07
149	F	0.28	0.04
150	F	0.32	0.09

#	Name	Parch value	
		avg	std
151	Q	0.11	0.01
152	F	0.05	0.04
153	Y	0.48	0.05
154	V	0.00	0.00
155	A	0.49	0.11
156	D	1.45	0.07
157	G	0.03	0.01
158	K	0.86	0.12
159	L	0.00	0.00
160	S	0.03	0.01
161	C	0.00	0.00
162	Q	0.17	0.05
163	L	0.01	0.00
164	Y	0.25	0.07
165	Q	0.07	0.02
166	R	0.69	0.24
167	S	0.28	0.03
168	C	0.08	0.02
169	D	0.17	0.04
170	V	0.00	0.00
171	F	0.00	0.00
172	L	0.84	0.11
173	G	0.04	0.01
174	L	0.00	0.00
175	P	0.02	0.02
176	F	0.49	0.16
177	N	0.02	0.01
178	I	0.01	0.01
179	A	0.01	0.01
180	S	0.07	0.03

#	Name	Parch value	
		avg	std
181	Y	0.06	0.01
182	A	0.00	0.00
183	L	0.01	0.01
184	L	0.00	0.01
185	V	0.01	0.02
186	H	0.16	0.04
187	M	0.00	0.00
188	M	0.01	0.01
189	A	0.01	0.00
190	Q	0.88	0.09
191	Q	0.25	0.01
192	C	0.02	0.01
193	D	1.62	0.14
194	L	0.18	0.05
195	E	1.61	0.22
196	V	0.09	0.03
197	G	0.00	0.01
198	D	0.61	0.11
199	F	0.01	0.00
200	V	0.07	0.03
201	W	0.07	0.02
202	T	0.09	0.02
203	G	0.04	0.01
204	G	0.11	0.03
205	D	0.22	0.04
206	T	0.15	0.05
207	H	0.07	0.02
208	L	0.00	0.00
209	Y	0.43	0.13
210	S	0.09	0.03

#	Name	Parch value	
		avg	std
211	N	0.56	0.07
212	H	0.10	0.03
213	M	0.05	0.01
214	D	0.98	0.08
215	Q	0.37	0.11
216	T	0.00	0.00
217	H	0.14	0.05
218	L	0.15	0.04
219	Q	0.00	0.00
220	L	0.07	0.04
221	S	0.15	0.04
222	R	1.16	0.09
223	E	2.16	0.44
224	P	0.36	0.09
225	R	1.02	0.11
226	P	0.13	0.02
227	L	0.46	0.03
228	P	0.01	0.01
229	K	1.26	0.13
230	L	0.08	0.05
231	I	0.51	0.03
232	I	0.23	0.07
233	K	0.68	0.06
234	R	1.06	0.06
235	A	0.31	0.05
236	P	0.19	0.06
237	E	2.86	0.18
238	S	0.27	0.04
239	I	0.03	0.01
240	F	0.30	0.06

PARCH values of TS residues (continued)

#	Name	Parch value	
		avg	std
241	D	1.05	0.02
242	Y	0.13	0.01
243	R	2.24	0.48
244	F	0.42	0.08
245	E	2.12	0.28
246	D	0.02	0.02
247	F	0.04	0.01
248	E	0.45	0.05
249	I	0.22	0.08
250	E	1.55	0.12
251	G	0.22	0.09
252	Y	0.36	0.08
253	D	0.95	0.14
254	P	0.20	0.05
255	H	0.38	0.09
256	P	0.25	0.04
257	G	0.28	0.06
258	I	0.47	0.17
259	K	1.15	0.17
260	A	0.06	0.02
261	P	0.16	0.07
262	V	0.06	0.01
263	A	0.11	0.03
264	I	1.06	0.28

Table S5. PARCH values of MDH residues

#	Name	Parch value		#	Name	Parch value		#	Name	Parch value		#	Name	Parch value	
		avg	std			avg	std			avg	std			avg	std
1	M	0.13	0.02	31	S	0.02	0.01	61	E	1.87	0.18	91	F	0.10	0.06
2	K	0.14	0.01	32	L	0.00	0.00	62	D	0.92	0.10	92	N	0.43	0.07
3	V	0.00	0.00	33	Y	0.02	0.01	63	A	0.03	0.02	93	V	0.07	0.02
4	A	0.02	0.01	34	D	0.03	0.01	64	T	0.23	0.04	94	N	0.00	0.00
5	V	0.00	0.00	35	I	0.16	0.04	65	P	0.34	0.05	95	A	0.03	0.01
6	L	0.02	0.01	36	A	0.02	0.01	66	A	0.01	0.01	96	G	0.05	0.02
7	G	0.00	0.00	37	P	0.03	0.02	67	L	0.00	0.00	97	I	0.05	0.03
8	A	0.00	0.00	38	V	0.06	0.03	68	E	0.77	0.13	98	V	0.13	0.03
9	A	0.05	0.01	39	T	0.00	0.00	69	G	0.35	0.08	99	K	0.84	0.07
10	G	0.22	0.04	40	P	0.08	0.06	70	A	0.01	0.01	100	N	0.13	0.04
11	G	0.18	0.03	41	G	0.05	0.03	71	D	0.81	0.02	101	L	0.02	0.02
12	I	0.17	0.02	42	V	0.03	0.02	72	V	0.00	0.00	102	V	0.01	0.01
13	G	0.00	0.00	43	A	0.01	0.02	73	V	0.00	0.00	103	Q	0.09	0.02
14	Q	0.16	0.03	44	V	0.11	0.03	74	L	0.00	0.00	104	Q	0.22	0.08
15	A	0.02	0.02	45	D	0.53	0.10	75	I	0.00	0.00	105	V	0.00	0.00
16	L	0.00	0.00	46	L	0.00	0.00	76	S	0.03	0.01	106	A	0.01	0.02
17	A	0.00	0.00	47	S	0.17	0.10	77	A	0.00	0.00	107	K	1.30	0.20
18	L	0.02	0.01	48	H	0.12	0.01	78	G	0.03	0.01	108	T	0.08	0.03
19	L	0.02	0.01	49	I	0.03	0.02	79	V	0.20	0.06	109	C	0.09	0.03
20	L	0.00	0.00	50	P	0.05	0.03	80	A	0.33	0.03	110	P	0.15	0.05
21	K	0.03	0.01	51	T	0.13	0.03	81	R	1.81	0.05	111	K	0.91	0.03
22	T	0.04	0.02	52	A	0.04	0.01	82	K	2.38	0.24	112	A	0.10	0.02
23	Q	0.26	0.14	53	V	0.01	0.02	83	P	0.98	0.18	113	C	0.00	0.01
24	L	0.02	0.02	54	K	0.54	0.07	84	G	0.14	0.01	114	I	0.01	0.01
25	P	0.15	0.06	55	I	0.02	0.00	85	M	0.26	0.05	115	G	0.00	0.00
26	S	0.21	0.09	56	K	0.92	0.15	86	D	0.40	0.08	116	I	0.00	0.00
27	G	0.06	0.02	57	G	0.07	0.02	87	R	1.41	0.13	117	I	0.01	0.01
28	S	0.00	0.00	58	F	0.40	0.06	88	S	0.23	0.10	118	T	0.01	0.01
29	E	0.59	0.09	59	S	0.08	0.06	89	D	0.69	0.12	119	N	0.07	0.02
30	L	0.00	0.01	60	G	0.03	0.02	90	L	0.03	0.01	120	P	0.09	0.04

PARCH values of MDH residues (continued)

#	Name	Parch value		#	Name	Parch value		#	Name	Parch value		#	Name	Parch value	
		avg	std			avg	std			avg	std			avg	std
121	V	0.15	0.06	151	I	0.01	0.00	181	T	0.04	0.01	211	T	0.41	0.02
122	N	0.03	0.01	152	I	0.04	0.02	182	I	0.04	0.01	212	E	1.15	0.15
123	T	0.00	0.01	153	R	0.64	0.07	183	L	0.02	0.01	213	V	0.73	0.24
124	T	0.07	0.04	154	S	0.01	0.01	184	P	0.01	0.01	214	V	0.30	0.06
125	V	0.00	0.00	155	N	0.00	0.00	185	L	0.03	0.02	215	E	1.23	0.06
126	A	0.00	0.00	156	T	0.09	0.06	186	L	0.16	0.02	216	A	0.13	0.06
127	I	0.20	0.01	157	F	0.10	0.05	187	S	0.46	0.04	217	K	0.98	0.15
128	A	0.05	0.02	158	V	0.00	0.00	188	Q	0.30	0.05	218	A	0.07	0.03
129	A	0.00	0.01	159	A	0.00	0.00	189	V	0.02	0.00	219	G	0.06	0.03
130	E	0.11	0.02	160	E	1.18	0.05	190	P	0.22	0.01	220	G	0.04	0.03
131	V	0.04	0.01	161	L	0.30	0.01	191	G	0.22	0.02	221	G	0.18	0.00
132	L	0.00	0.00	162	K	0.80	0.05	192	V	0.18	0.07	222	S	0.77	0.02
133	K	0.69	0.02	163	G	0.06	0.04	193	S	0.64	0.13	223	A	0.15	0.04
134	K	0.64	0.15	164	K	0.73	0.03	194	F	0.22	0.04	224	T	0.29	0.04
135	A	0.07	0.04	165	Q	0.37	0.07	195	T	0.03	0.01	225	L	0.31	0.02
136	G	0.03	0.02	166	P	0.16	0.06	196	E	1.82	0.24	226	S	0.12	0.01
137	V	0.07	0.03	167	G	0.26	0.05	197	Q	0.41	0.14	227	M	0.09	0.03
138	Y	0.30	0.07	168	E	0.37	0.04	198	E	1.42	0.02	228	G	0.00	0.00
139	D	0.24	0.03	169	V	0.06	0.02	199	V	0.04	0.01	229	Q	0.20	0.02
140	K	0.93	0.12	170	E	1.07	0.07	200	A	0.11	0.04	230	A	0.00	0.01
141	N	0.19	0.02	171	V	0.02	0.01	201	D	0.82	0.07	231	A	0.00	0.00
142	K	0.38	0.06	172	P	0.01	0.01	202	L	0.02	0.01	232	A	0.00	0.00
143	L	0.00	0.00	173	V	0.00	0.00	203	T	0.01	0.01	233	R	0.92	0.09
144	F	0.00	0.00	174	I	0.00	0.00	204	K	1.22	0.20	234	F	0.00	0.00
145	G	0.00	0.00	175	G	0.00	0.00	205	R	1.98	0.07	235	G	0.00	0.00
146	V	0.20	0.06	176	G	0.00	0.00	206	I	0.04	0.03	236	L	0.04	0.05
147	T	0.05	0.02	177	H	0.34	0.03	207	Q	0.18	0.04	237	S	0.04	0.00
148	T	0.01	0.01	178	S	0.03	0.01	208	N	0.21	0.02	238	L	0.00	0.00
149	L	0.11	0.01	179	G	0.08	0.02	209	A	0.23	0.03	239	V	0.00	0.00
150	D	0.04	0.00	180	V	0.18	0.01	210	G	0.07	0.03	240	R	0.80	0.15

PARCH values of MDH residues (continued)

#	Name	Parch value	
		avg	std
241	A	0.00	0.00
242	L	0.13	0.03
243	Q	0.56	0.06
244	G	0.03	0.01
245	E	0.63	0.04
246	Q	0.75	0.08
247	G	0.20	0.05
248	V	0.14	0.04
249	V	0.22	0.08
250	E	0.01	0.01
251	C	0.01	0.01
252	A	0.00	0.00
253	Y	0.02	0.02
254	V	0.00	0.00
255	E	0.43	0.07
256	G	0.24	0.04
257	D	0.82	0.09
258	G	0.17	0.02
259	Q	0.68	0.18
260	Y	0.51	0.09
261	A	0.05	0.02
262	R	2.00	0.15
263	F	0.02	0.01
264	F	0.03	0.03
265	S	0.00	0.00
266	Q	0.06	0.02
267	P	0.06	0.04
268	L	0.00	0.00
269	L	0.12	0.04
270	L	0.02	0.01

#	Name	Parch value	
		avg	std
271	G	0.10	0.03
272	K	1.03	0.27
273	N	0.64	0.20
274	G	0.01	0.01
275	V	0.03	0.02
276	E	0.93	0.05
277	E	0.76	0.15
278	R	0.25	0.01
279	K	0.70	0.03
280	S	0.43	0.07
281	I	0.15	0.05
282	G	0.15	0.05
283	T	0.37	0.04
284	L	0.17	0.06
285	S	0.26	0.07
286	A	0.06	0.03
287	F	0.14	0.09
288	E	0.32	0.02
289	Q	0.25	0.11
290	N	0.29	0.05
291	A	0.00	0.01
292	L	0.00	0.01
293	E	1.76	0.21
294	G	0.10	0.03
295	M	0.01	0.01
296	L	0.04	0.03
297	D	0.66	0.07
298	T	0.24	0.03
299	L	0.00	0.01
300	K	0.59	0.07

#	Name	Parch value	
		avg	std
301	K	0.99	0.04
302	D	0.27	0.03
303	I	0.01	0.01
304	A	0.15	0.03
305	L	0.35	0.13
306	G	0.00	0.00
307	E	0.68	0.09
308	E	0.89	0.18
309	F	0.20	0.02
310	V	0.12	0.04
311	N	0.26	0.13
312	K	2.87	0.40

Table S6. PARCH values of BNS residues

#	Name	Parch value		#	Name	Parch value		#	Name	Parch value		#	Name	Parch value	
		avg	std			avg	std			avg	std			avg	std
1	A	0.75	0.06	31	Q	0.20	0.06	61	G	0.16	0.05	91	S	0.00	0.01
2	Q	0.21	0.08	32	A	0.02	0.02	62	K	0.75	0.16	92	S	0.07	0.03
3	V	0.37	0.05	33	L	0.28	0.05	63	L	0.02	0.01	93	D	0.26	0.03
4	I	0.11	0.04	34	G	0.00	0.01	64	P	0.05	0.01	94	W	0.28	0.11
5	N	0.05	0.01	35	W	0.14	0.01	65	G	0.16	0.03	95	L	0.20	0.05
6	T	0.24	0.11	36	V	0.13	0.04	66	K	2.17	0.28	96	I	0.00	0.00
7	F	0.33	0.03	37	A	0.12	0.01	67	S	0.26	0.04	97	Y	0.05	0.02
8	D	0.68	0.13	38	S	0.10	0.04	68	G	0.11	0.02	98	K	0.47	0.05
9	G	0.03	0.02	39	K	1.26	0.14	69	R	0.56	0.13	99	T	0.02	0.00
10	V	0.00	0.00	40	G	0.01	0.01	70	T	0.17	0.05	100	T	0.42	0.04
11	A	0.00	0.00	41	N	0.10	0.02	71	W	0.03	0.01	101	D	0.18	0.04
12	D	0.28	0.06	42	L	0.07	0.01	72	R	0.77	0.08	102	H	0.53	0.12
13	Y	0.14	0.03	43	A	0.21	0.03	73	E	0.48	0.04	103	Y	0.86	0.16
14	L	0.00	0.00	44	D	1.00	0.09	74	A	0.13	0.07	104	Q	0.39	0.05
15	Q	0.12	0.02	45	V	0.21	0.02	75	D	0.02	0.01	105	T	0.20	0.05
16	T	0.21	0.04	46	A	0.09	0.01	76	I	0.00	0.00	106	F	0.19	0.02
17	Y	0.23	0.05	47	P	0.22	0.03	77	N	0.23	0.05	107	T	0.19	0.04
18	H	0.71	0.07	48	G	0.20	0.09	78	Y	0.33	0.16	108	K	0.69	0.07
19	K	1.17	0.04	49	K	0.85	0.13	79	T	0.30	0.08	109	I	0.06	0.04
20	L	0.11	0.02	50	S	0.02	0.01	80	S	0.28	0.03	110	R	1.07	0.18
21	P	0.04	0.02	51	I	0.05	0.01	81	G	0.08	0.04				
22	D	1.39	0.15	52	G	0.01	0.01	82	F	1.07	0.29				
23	N	0.10	0.03	53	G	0.17	0.05	83	R	0.84	0.07				
24	Y	0.02	0.00	54	D	0.31	0.09	84	N	0.08	0.02				
25	I	0.22	0.06	55	I	0.53	0.09	85	S	0.29	0.08				
26	T	0.23	0.03	56	F	0.17	0.07	86	D	0.10	0.02				
27	K	0.34	0.05	57	S	0.30	0.03	87	R	0.21	0.07				
28	S	0.14	0.03	58	N	0.09	0.02	88	I	0.05	0.01				
29	E	0.34	0.05	59	R	1.81	0.16	89	L	0.00	0.00				
30	A	0.02	0.01	60	E	1.15	0.13	90	Y	0.30	0.03				

Table S7. PARCH values of MDM2 residues

#	Name	Parch value	
		avg	std
1	S	0.82	0.04
2	Q	0.60	0.10
3	I	0.04	0.02
4	P	0.20	0.05
5	A	0.14	0.02
6	S	0.11	0.05
7	E	0.23	0.11
8	Q	0.13	0.05
9	E	0.29	0.03
10	T	0.06	0.02
11	L	0.20	0.07
12	V	0.00	0.01
13	R	0.39	0.08
14	P	0.03	0.02
15	K	0.85	0.13
16	P	0.08	0.05
17	L	0.06	0.03
18	L	0.04	0.00
19	L	0.02	0.02
20	K	0.56	0.03
21	L	0.03	0.02
22	L	0.01	0.01
23	K	0.43	0.03
24	S	0.07	0.02
25	V	0.03	0.02
26	G	0.04	0.02
27	A	0.07	0.03
28	Q	0.54	0.06
29	K	0.75	0.08
30	D	0.24	0.03
31	T	0.12	0.05
32	Y	0.01	0.01
33	T	0.05	0.02
34	M	0.00	0.00
35	K	0.70	0.15
36	E	0.07	0.01
37	V	0.02	0.01
38	L	0.05	0.04
39	F	0.11	0.06
40	Y	0.08	0.01
41	L	0.05	0.04
42	G	0.02	0.01
43	Q	0.07	0.04
44	Y	0.08	0.02
45	I	0.00	0.00
46	M	0.13	0.02
47	T	0.13	0.01
48	K	1.03	0.09
49	R	1.09	0.17
50	L	0.02	0.00
51	Y	0.59	0.13
52	D	0.97	0.10
53	E	1.76	0.16
54	K	1.04	0.33
55	Q	0.82	0.15
56	Q	0.75	0.10
57	H	0.34	0.07
58	I	0.42	0.14
59	V	0.00	0.00
60	Y	0.34	0.08
61	C	0.00	0.01
62	S	0.19	0.05
63	N	0.96	0.04
64	D	1.12	0.08
65	L	1.00	0.29
66	L	0.00	0.01
67	G	0.02	0.02
68	D	1.10	0.13
69	L	0.11	0.01
70	F	0.08	0.03
71	G	0.15	0.03
72	V	0.05	0.01
73	P	0.09	0.04
74	S	0.07	0.01
75	F	0.04	0.03
76	S	0.24	0.03
77	V	0.08	0.03
78	K	1.42	0.06
79	E	0.88	0.16
80	H	0.14	0.02
81	R	0.91	0.07
82	K	0.94	0.19
83	I	0.03	0.01
84	Y	0.05	0.02
85	T	0.07	0.02
86	M	0.14	0.09
87	I	0.00	0.00
88	Y	0.31	0.03
89	R	1.87	0.23
90	N	0.21	0.07
91	L	0.05	0.01
92	V	0.04	0.01
93	V	0.11	0.04
94	V	0.11	0.01
95	N	0.49	0.11
96	Q	0.13	0.05
97	Q	0.38	0.04
98	E	1.50	0.16
99	S	0.43	0.13
100	S	0.64	0.07
101	D	0.77	0.10
102	S	0.10	0.06
103	G	0.27	0.04
104	T	0.28	0.08
105	S	0.26	0.03
106	V	0.32	0.03
107	S	0.28	0.05
108	E	1.12	0.09
109	N	1.49	0.04

Table S8. PARCH values of MBP(M) residues

#	Name	Parch value	
		avg	std
1	S	0.93	0.10
2	G	0.22	0.10
3	K	1.29	0.12
4	K	0.40	0.08
5	F	0.19	0.03
6	F	0.04	0.02
7	V	0.02	0.01
8	T	0.04	0.02
9	N	0.07	0.03
10	H	0.46	0.03
11	E	0.80	0.05
12	R	0.77	0.08
13	M	0.27	0.13
14	P	0.09	0.03
15	F	0.05	0.04
16	S	0.16	0.09
17	K	0.95	0.16
18	V	0.00	0.00
19	K	0.94	0.23
20	A	0.06	0.05
21	L	0.16	0.09
22	C	0.00	0.01
23	S	0.06	0.02
24	E	1.00	0.15
25	L	0.19	0.05
26	R	0.65	0.18
27	G	0.03	0.02
28	T	0.11	0.00
29	V	0.00	0.00
30	A	0.00	0.00
31	I	0.03	0.02
32	P	0.00	0.00
33	R	1.09	0.11
34	N	0.09	0.02
35	A	0.03	0.02
36	E	0.34	0.06
37	E	0.24	0.05
38	N	0.00	0.01
39	K	0.71	0.06
40	A	0.11	0.04
41	I	0.01	0.01
42	Q	0.17	0.02
43	E	1.14	0.17
44	V	0.04	0.01
45	A	0.13	0.02
46	K	0.78	0.14
47	T	0.53	0.08
48	S	0.11	0.04
49	A	0.01	0.01
50	F	0.01	0.01
51	L	0.00	0.00
52	G	0.00	0.00
53	I	0.00	0.00
54	T	0.16	0.05
55	D	0.24	0.07
56	E	2.03	0.15
57	V	0.41	0.05
58	T	0.51	0.10
59	E	1.53	0.15
60	G	0.31	0.04
61	Q	0.63	0.12
62	F	0.53	0.16
63	M	0.08	0.03
64	Y	0.00	0.00
65	V	0.11	0.03
66	T	0.31	0.03
67	G	0.10	0.02
68	G	0.06	0.02
69	R	1.23	0.38
70	L	0.12	0.03
71	T	0.16	0.04
72	Y	0.07	0.01
73	S	0.07	0.02
74	N	0.08	0.00
75	W	0.51	0.10
76	K	1.41	0.07
77	K	1.17	0.04
78	D	0.81	0.15
79	E	1.66	0.19
80	P	0.57	0.08
81	N	0.80	0.19
82	D	0.99	0.12
83	H	0.85	0.15
84	G	0.09	0.01
85	S	0.84	0.07
86	G	0.25	0.03
87	E	1.15	0.10
88	D	0.93	0.22
89	C	0.04	0.01
90	V	0.04	0.01
91	T	0.01	0.00
92	I	0.05	0.01
93	V	0.09	0.01
94	D	0.57	0.15
95	N	0.31	0.08
96	G	0.00	0.00
97	L	0.10	0.01
98	W	0.00	0.00
99	N	0.22	0.09
100	D	0.32	0.01
101	I	0.04	0.00
102	S	0.21	0.07
103	C	0.32	0.04
104	Q	0.34	0.03
105	A	0.01	0.01
106	S	0.05	0.02
107	H	0.04	0.00
108	T	0.07	0.02
109	A	0.00	0.00
110	V	0.00	0.00
111	C	0.00	0.00
112	E	0.14	0.02
113	F	0.03	0.01
114	P	0.23	0.03
115	A	1.27	0.22

Table S9. PARCH values of MBP(D) residues

#	Name	Parch value	
		avg	std
1	S	1.08	0.18
2	G	0.30	0.13
3	K	1.71	0.35
4	K	1.45	0.13
5	F	0.07	0.02
6	F	0.00	0.01
7	V	0.00	0.01
8	T	0.00	0.00
9	N	0.01	0.00
10	H	0.22	0.04
11	E	0.22	0.03
12	R	1.10	0.10
13	M	0.06	0.02
14	P	0.09	0.04
15	F	0.15	0.07
16	S	0.57	0.09
17	K	0.74	0.14
18	V	0.01	0.01
19	K	1.02	0.07
20	A	0.12	0.03
21	L	0.01	0.01
22	C	0.01	0.00
23	S	0.21	0.06
24	E	1.04	0.15
25	L	0.02	0.02
26	R	1.08	0.27
27	G	0.04	0.02
28	T	0.22	0.04
29	V	0.03	0.01
30	A	0.00	0.01
31	I	0.09	0.01
32	P	0.01	0.00
33	R	1.20	0.12
34	N	0.22	0.08
35	A	0.12	0.05
36	E	1.32	0.17
37	E	0.26	0.04
38	N	0.00	0.00
39	K	0.93	0.10
40	A	0.05	0.02
41	I	0.00	0.01
42	Q	0.06	0.03
43	E	1.31	0.22
44	V	0.04	0.02
45	A	0.00	0.00
46	K	1.42	0.27
47	T	0.70	0.18
48	S	0.20	0.09
49	A	0.00	0.00
50	F	0.02	0.01
51	L	0.00	0.01
52	G	0.00	0.00
53	I	0.00	0.00
54	T	0.17	0.05
55	D	0.77	0.07
56	E	2.25	0.20
57	V	0.13	0.02
58	T	0.38	0.06
59	E	2.15	0.16
60	G	0.62	0.18
61	Q	0.96	0.09
62	F	0.39	0.02
63	M	0.04	0.04
64	Y	0.01	0.01
65	V	0.06	0.05
66	T	0.16	0.06
67	G	0.05	0.02
68	G	0.02	0.01
69	R	0.98	0.02
70	L	0.12	0.03
71	T	0.11	0.01
72	Y	0.11	0.01
73	S	0.12	0.03
74	N	0.12	0.04
75	W	0.58	0.10
76	K	1.79	0.53
77	K	2.05	0.23
78	D	1.19	0.08
79	E	2.19	0.34
80	P	1.30	0.12
81	N	1.03	0.15
82	D	1.69	0.14
83	H	0.28	0.06
84	G	0.14	0.07
85	S	0.27	0.03
86	G	0.44	0.06
87	E	1.59	0.08
88	D	1.61	0.25
89	C	0.04	0.01
90	V	0.01	0.01
91	T	0.00	0.00
92	I	0.00	0.00
93	V	0.04	0.01
94	D	1.02	0.17
95	N	0.25	0.09
96	G	0.00	0.00
97	L	0.04	0.01
98	W	0.01	0.00
99	N	0.51	0.16
100	D	0.57	0.03
101	I	0.10	0.02
102	S	0.25	0.08
103	C	0.57	0.21
104	Q	0.44	0.13
105	A	0.09	0.04
106	S	0.19	0.03
107	H	0.30	0.01
108	T	0.00	0.01
109	A	0.00	0.00
110	V	0.02	0.02
111	C	0.00	0.00
112	E	0.25	0.04
113	F	0.08	0.02
114	P	0.29	0.06
115	A	0.58	0.05
116	S	0.89	0.10
117	G	0.11	0.02
118	K	0.57	0.08
119	K	0.19	0.02
120	F	0.08	0.01

PARCH values of MBP(D) residues (continued)

#	Name	Parch value	
		avg	std
121	F	0.01	0.00
122	V	0.14	0.01
123	T	0.06	0.03
124	N	0.30	0.01
125	H	0.74	0.16
126	E	2.03	0.06
127	R	1.55	0.14
128	M	0.10	0.03
129	P	0.06	0.01
130	F	0.02	0.02
131	S	0.24	0.06
132	K	0.73	0.10
133	V	0.01	0.01
134	K	0.79	0.03
135	A	0.13	0.15
136	L	0.01	0.01
137	C	0.00	0.00
138	S	0.05	0.03
139	E	0.48	0.06
140	L	0.00	0.01
141	R	0.91	0.10
142	G	0.01	0.01
143	T	0.19	0.07
144	V	0.08	0.01
145	A	0.01	0.00
146	I	0.07	0.04
147	P	0.00	0.00
148	R	0.22	0.01
149	N	0.16	0.02
150	A	0.07	0.03
151	E	0.67	0.10
152	E	0.20	0.02
153	N	0.00	0.00
154	K	1.24	0.12
155	A	0.04	0.03
156	I	0.00	0.00
157	Q	0.09	0.06
158	E	1.55	0.07
159	V	0.10	0.02
160	A	0.03	0.01
161	K	1.04	0.08
162	T	0.51	0.18
163	S	0.08	0.03
164	A	0.00	0.00
165	F	0.00	0.00
166	L	0.03	0.01
167	G	0.00	0.00
168	I	0.00	0.00
169	T	0.05	0.01
170	D	0.19	0.01
171	E	1.79	0.03
172	V	0.35	0.07
173	T	0.17	0.11
174	E	1.92	0.14
175	G	0.32	0.03
176	Q	0.55	0.13
177	F	0.25	0.05
178	M	0.01	0.01
179	Y	0.00	0.00
180	V	0.08	0.02
181	T	0.11	0.04
182	G	0.02	0.02
183	G	0.03	0.01
184	R	0.83	0.05
185	L	0.04	0.02
186	T	0.06	0.02
187	Y	0.17	0.02
188	S	0.21	0.06
189	N	0.21	0.05
190	W	0.21	0.03
191	K	0.68	0.05
192	K	1.82	0.24
193	D	1.31	0.32
194	E	1.03	0.16
195	P	0.89	0.09
196	N	0.47	0.14
197	D	1.29	0.24
198	H	0.30	0.13
199	G	0.14	0.05
200	S	0.58	0.01
201	G	0.41	0.13
202	E	1.07	0.07
203	D	0.33	0.04
204	C	0.00	0.00
205	V	0.00	0.00
206	T	0.00	0.00
207	I	0.03	0.01
208	V	0.05	0.03
209	D	0.86	0.05
210	N	0.24	0.02
211	G	0.00	0.00
212	L	0.07	0.02
213	W	0.03	0.02
214	N	0.05	0.01
215	D	0.19	0.03
216	I	0.14	0.06
217	S	0.31	0.06
218	C	0.11	0.00
219	Q	0.35	0.02
220	A	0.04	0.02
221	S	0.13	0.07
222	H	0.17	0.04
223	T	0.05	0.01
224	A	0.00	0.00
225	V	0.05	0.03
226	C	0.01	0.00
227	E	0.24	0.03
228	F	0.02	0.02
229	P	0.26	0.19
230	A	0.35	0.04

Table S10. PARCH values of HP2 residues

#	Name	Parch value	
		avg	std
1	A	1.46	0.08
2	V	0.13	0.05
3	C	0.11	0.02
4	P	0.05	0.01
5	T	0.28	0.04
6	G	0.04	0.01
7	L	0.16	0.03
8	F	0.15	0.04
9	S	0.39	0.03
10	N	0.11	0.02
11	P	0.05	0.02
12	L	0.09	0.02
13	C	0.00	0.00
14	C	0.05	0.01
15	A	0.16	0.05
16	T	0.12	0.02
17	N	0.18	0.05
18	V	0.03	0.02
19	L	0.19	0.08
20	D	1.44	0.17
21	L	0.07	0.05
22	I	0.00	0.00
23	G	0.03	0.01
24	V	0.01	0.00
25	D	0.33	0.05
26	C	0.12	0.01
27	K	0.58	0.03
28	T	0.38	0.04
29	P	0.08	0.01
30	T	0.35	0.06

#	Name	Parch value	
		avg	std
31	I	0.32	0.02
32	A	0.28	0.07
33	V	0.19	0.03
34	D	1.61	0.17
35	T	0.38	0.09
36	G	0.04	0.01
37	A	0.02	0.00
38	I	0.16	0.07
39	F	0.01	0.01
40	Q	0.13	0.02
41	A	0.00	0.00
42	H	0.09	0.03
43	C	0.00	0.00
44	A	0.04	0.03
45	S	0.17	0.05
46	K	1.32	0.28
47	G	0.10	0.04
48	S	0.04	0.01
49	K	0.79	0.11
50	P	0.01	0.00
51	L	0.10	0.02
52	C	0.00	0.01
53	C	0.17	0.00
54	V	0.08	0.01
55	A	0.03	0.02
56	P	0.11	0.04
57	V	0.49	0.05
58	A	0.23	0.06
59	D	0.94	0.07
60	Q	0.35	0.05

#	Name	Parch value	
		avg	std
61	A	0.05	0.02
62	L	0.33	0.08
63	L	0.13	0.03
64	C	0.03	0.01
65	Q	0.20	0.01
66	K	1.17	0.14
67	A	0.01	0.01
68	I	0.31	0.08
69	G	0.06	0.02
70	T	0.12	0.05
71	F	0.66	0.06

Table S11. PARCH values of HBV(M) residues

#	Name	Parch value	
		avg	std
1	M	0.88	0.17
2	D	1.69	0.30
3	I	0.48	0.23
4	D	0.97	0.13
5	P	0.23	0.07
6	Y	0.30	0.04
7	K	1.28	0.14
8	E	1.67	0.24
9	F	0.09	0.01
10	G	0.03	0.01
11	A	0.03	0.02
12	T	0.22	0.05
13	V	0.35	0.05
14	E	1.32	0.06
15	L	0.06	0.03
16	L	0.15	0.08
17	S	0.28	0.11
18	F	0.21	0.06
19	L	0.06	0.03
20	P	0.38	0.10
21	S	0.18	0.03
22	D	1.29	0.16
23	F	0.19	0.03
24	F	0.13	0.06
25	P	0.14	0.04
26	S	0.19	0.01
27	V	0.04	0.03
28	R	1.87	0.28
29	D	0.22	0.04
30	L	0.01	0.01

#	Name	Parch value	
		avg	std
31	L	0.03	0.02
32	D	0.17	0.03
33	T	0.02	0.01
34	A	0.00	0.00
35	S	0.21	0.08
36	A	0.03	0.01
37	L	0.08	0.02
38	Y	0.04	0.02
39	R	0.95	0.09
40	E	1.10	0.00
41	A	0.10	0.03
42	L	0.16	0.03
43	E	1.00	0.07
44	S	0.10	0.01
45	P	0.17	0.07
46	E	1.12	0.12
47	H	0.67	0.17
48	C	0.17	0.03
49	S	0.10	0.04
50	P	0.10	0.03
51	H	0.08	0.01
52	H	0.06	0.02
53	T	0.21	0.06
54	A	0.02	0.01
55	L	0.00	0.01
56	R	0.76	0.08
57	Q	0.23	0.02
58	A	0.00	0.00
59	I	0.06	0.01
60	L	0.10	0.02

#	Name	Parch value	
		avg	std
61	C	0.07	0.05
62	W	0.11	0.02
63	G	0.02	0.02
64	E	0.58	0.02
65	L	0.00	0.00
66	M	0.04	0.02
67	T	0.07	0.03
68	L	0.01	0.01
69	A	0.00	0.01
70	T	0.11	0.02
71	W	0.06	0.02
72	V	0.01	0.00
73	G	0.01	0.01
74	N	0.24	0.07
75	N	0.09	0.04
76	L	0.14	0.08
77	E	1.90	0.13
78	D	0.26	0.06
79	P	0.28	0.09
80	A	0.14	0.03
81	S	0.02	0.02
82	R	1.33	0.16
83	D	0.60	0.11
84	L	0.05	0.01
85	V	0.00	0.00
86	V	0.05	0.01
87	N	0.07	0.02
88	Y	0.05	0.03
89	V	0.00	0.00
90	N	0.13	0.01

#	Name	Parch value	
		avg	std
91	T	0.09	0.04
92	N	0.06	0.02
93	M	0.03	0.02
94	G	0.00	0.00
95	L	0.10	0.08
96	K	0.73	0.08
97	I	0.00	0.00
98	R	0.28	0.03
99	Q	0.08	0.04
100	L	0.04	0.03
101	L	0.00	0.01
102	W	0.07	0.01
103	F	0.03	0.01
104	H	0.02	0.00
105	I	0.02	0.01
106	S	0.00	0.00
107	C	0.00	0.00
108	L	0.11	0.02
109	T	0.06	0.02
110	F	0.11	0.02
111	G	0.07	0.00
112	R	0.58	0.03
113	E	1.81	0.26
114	T	0.16	0.06
115	V	0.00	0.00
116	L	0.07	0.01
117	E	0.54	0.03
118	Y	0.10	0.01
119	L	0.04	0.03
120	V	0.06	0.01

PARCH values of HBV(M) residues (continued)

#	Name	Parch value	
		avg	std
121	S	0.04	0.01
122	F	0.02	0.01
123	G	0.00	0.00
124	V	0.05	0.01
125	W	0.11	0.06
126	I	0.14	0.04
127	R	1.49	0.24
128	T	0.19	0.03
129	P	0.12	0.01
130	P	0.15	0.06
131	A	0.04	0.01
132	Y	0.15	0.02
133	R	0.50	0.05
134	P	0.10	0.01
135	P	0.39	0.06
136	N	0.44	0.05
137	A	0.11	0.02
138	P	0.05	0.02
139	I	0.38	0.05
140	L	0.20	0.12
141	S	0.25	0.07
142	T	0.34	0.06
143	L	0.49	0.24
144	P	0.34	0.02
145	E	2.96	0.16
146	T	0.16	0.05
147	T	0.32	0.01
148	V	0.34	0.14
149	V	1.58	0.11

Table S12. PARCH values of HBV(D) residues

#	Name	Parch value	
		avg	std
1	M	1.20	0.33
2	D	1.43	0.19
3	I	0.07	0.01
4	D	0.69	0.01
5	P	0.42	0.06
6	Y	0.05	0.02
7	K	1.09	0.05
8	E	0.17	0.01
9	F	0.03	0.01
10	G	0.06	0.02
11	A	0.01	0.01
12	T	0.24	0.05
13	V	0.21	0.02
14	E	0.94	0.15
15	L	0.01	0.01
16	L	0.00	0.00
17	S	0.35	0.06
18	F	0.30	0.06
19	L	0.00	0.01
20	P	0.26	0.08
21	S	0.30	0.07
22	D	1.22	0.29
23	F	0.27	0.06
24	F	0.10	0.06
25	P	0.11	0.05
26	S	0.28	0.06
27	V	0.05	0.03
28	R	1.30	0.10
29	D	0.29	0.05
30	L	0.00	0.00
31	L	0.09	0.05
32	D	0.39	0.11
33	T	0.00	0.00
34	A	0.00	0.00
35	S	0.19	0.07
36	A	0.09	0.05
37	L	0.03	0.01
38	Y	0.07	0.02
39	R	1.26	0.09
40	E	1.16	0.24
41	A	0.11	0.05
42	L	0.00	0.01
43	E	0.70	0.07
44	S	0.28	0.18
45	P	0.18	0.13
46	E	1.29	0.21
47	H	0.25	0.07
48	C	0.39	0.05
49	S	0.18	0.03
50	P	0.03	0.01
51	H	0.02	0.02
52	H	0.16	0.06
53	T	0.01	0.01
54	A	0.00	0.00
55	L	0.00	0.00
56	R	0.10	0.04
57	Q	0.01	0.00
58	A	0.00	0.00
59	I	0.03	0.02
60	L	0.13	0.04
61	C	0.00	0.01
62	W	0.04	0.01
63	G	0.05	0.01
64	E	0.25	0.10
65	L	0.01	0.00
66	M	0.06	0.03
67	T	0.13	0.01
68	L	0.04	0.00
69	A	0.01	0.00
70	T	0.06	0.02
71	W	0.03	0.02
72	V	0.05	0.02
73	G	0.03	0.01
74	N	0.47	0.08
75	N	0.11	0.03
76	L	0.23	0.07
77	E	1.88	0.31
78	D	0.40	0.06
79	P	0.24	0.14
80	A	0.11	0.05
81	S	0.04	0.01
82	R	1.15	0.04
83	D	0.87	0.16
84	L	0.07	0.05
85	V	0.00	0.01
86	V	0.06	0.06
87	N	0.13	0.05
88	Y	0.03	0.03
89	V	0.01	0.00
90	N	0.05	0.01
91	T	0.06	0.01
92	N	0.08	0.01
93	M	0.04	0.01
94	G	0.00	0.00
95	L	0.11	0.05
96	K	0.58	0.11
97	I	0.00	0.00
98	R	0.17	0.02
99	Q	0.00	0.00
100	L	0.01	0.01
101	L	0.00	0.00
102	W	0.05	0.01
103	F	0.00	0.00
104	H	0.00	0.00
105	I	0.00	0.00
106	S	0.00	0.01
107	C	0.00	0.01
108	L	0.15	0.04
109	T	0.05	0.01
110	F	0.14	0.02
111	G	0.09	0.04
112	R	0.84	0.04
113	E	1.75	0.27
114	T	0.41	0.12
115	V	0.00	0.00
116	L	0.04	0.02
117	E	0.84	0.10
118	Y	0.02	0.01
119	L	0.00	0.00
120	V	0.01	0.01

PARCH values of HBV(D) residues (continued)

#	Name	Parch value	
		avg	std
121	S	0.03	0.01
122	F	0.00	0.01
123	G	0.00	0.00
124	V	0.02	0.01
125	W	0.13	0.03
126	I	0.13	0.06
127	R	0.88	0.06
128	T	0.02	0.01
129	P	0.03	0.01
130	P	0.12	0.03
131	A	0.04	0.02
132	Y	0.25	0.21
133	R	0.39	0.04
134	P	0.24	0.08
135	P	0.27	0.01
136	N	0.16	0.05
137	A	0.11	0.06
138	P	0.07	0.01
139	I	0.35	0.05
140	L	0.08	0.02
141	S	0.08	0.02
142	T	0.36	0.09
143	L	0.74	0.19
144	P	0.28	0.09
145	E	2.28	0.11
146	T	0.21	0.09
147	T	0.17	0.05
148	V	0.39	0.05
149	V	1.28	0.12
150	M	1.00	0.02

#	Name	Parch value	
		avg	std
151	D	1.55	0.09
152	I	0.16	0.04
153	D	1.19	0.24
154	P	0.46	0.15
155	Y	0.13	0.02
156	K	0.99	0.13
157	E	0.16	0.04
158	F	0.03	0.01
159	G	0.08	0.04
160	A	0.10	0.01
161	T	0.40	0.09
162	V	0.33	0.08
163	E	0.53	0.16
164	L	0.11	0.01
165	L	0.09	0.02
166	S	0.31	0.11
167	F	0.08	0.03
168	L	0.04	0.00
169	P	0.23	0.09
170	S	0.35	0.12
171	D	1.03	0.09
172	F	0.17	0.03
173	F	0.04	0.01
174	P	0.14	0.02
175	S	0.25	0.01
176	V	0.04	0.02
177	R	1.85	0.17
178	D	0.54	0.11
179	L	0.01	0.01
180	L	0.22	0.06

#	Name	Parch value	
		avg	std
181	D	0.89	0.03
182	T	0.02	0.01
183	A	0.01	0.00
184	S	0.13	0.02
185	A	0.25	0.01
186	L	0.13	0.06
187	Y	0.14	0.05
188	R	0.77	0.12
189	E	1.62	0.15
190	A	0.04	0.01
191	L	0.01	0.01
192	E	0.74	0.18
193	S	0.30	0.05
194	P	0.42	0.08
195	E	2.27	0.28
196	H	0.20	0.05
197	C	0.52	0.03
198	S	0.10	0.02
199	P	0.05	0.04
200	H	0.43	0.06
201	H	0.35	0.06
202	T	0.04	0.02
203	A	0.00	0.00
204	L	0.02	0.01
205	R	0.19	0.03
206	Q	0.07	0.01
207	A	0.00	0.00
208	I	0.02	0.01
209	L	0.15	0.01
210	C	0.01	0.01

#	Name	Parch value	
		avg	std
211	W	0.14	0.03
212	G	0.02	0.01
213	E	0.14	0.02
214	L	0.00	0.00
215	M	0.09	0.02
216	T	0.11	0.01
217	L	0.07	0.03
218	A	0.03	0.01
219	T	0.03	0.01
220	W	0.08	0.03
221	V	0.05	0.01
222	G	0.00	0.00
223	N	0.29	0.04
224	N	0.29	0.02
225	L	0.13	0.02
226	E	1.49	0.11
227	D	0.10	0.05
228	P	0.45	0.18
229	A	0.22	0.01
230	S	0.10	0.01
231	R	0.49	0.12
232	D	0.80	0.08
233	L	0.07	0.03
234	V	0.00	0.01
235	V	0.12	0.03
236	N	0.17	0.05
237	Y	0.06	0.02
238	V	0.00	0.00
239	N	0.08	0.01
240	T	0.19	0.07

PARCH values of HBV(D) residues (continued)

#	Name	Parch value	
		avg	std
241	N	0.07	0.03
242	M	0.01	0.01
243	G	0.00	0.00
244	L	0.01	0.01
245	K	0.45	0.12
246	I	0.02	0.01
247	R	0.17	0.02
248	Q	0.10	0.02
249	L	0.03	0.01
250	L	0.01	0.00
251	W	0.07	0.03
252	F	0.02	0.01
253	H	0.01	0.01
254	I	0.04	0.01
255	S	0.01	0.00
256	C	0.00	0.01
257	L	0.07	0.04
258	T	0.10	0.03
259	F	0.09	0.02
260	G	0.06	0.03
261	R	0.57	0.06
262	E	0.97	0.09
263	T	0.16	0.03
264	V	0.00	0.00
265	L	0.07	0.02
266	E	1.07	0.21
267	Y	0.07	0.04
268	L	0.01	0.01
269	V	0.04	0.04
270	S	0.12	0.03

#	Name	Parch value	
		avg	std
271	F	0.02	0.01
272	G	0.00	0.00
273	V	0.05	0.04
274	W	0.14	0.03
275	I	0.27	0.05
276	R	0.76	0.22
277	T	0.01	0.01
278	P	0.06	0.02
279	P	0.10	0.04
280	A	0.04	0.01
281	Y	0.14	0.07
282	R	0.25	0.02
283	P	0.11	0.02
284	P	0.11	0.04
285	N	0.22	0.03
286	A	0.03	0.02
287	P	0.07	0.02
288	I	0.28	0.02
289	L	0.08	0.02
290	S	0.07	0.01
291	T	0.14	0.13
292	L	0.17	0.03
293	P	0.33	0.09
294	E	1.96	0.06
295	T	0.25	0.06
296	T	0.39	0.06
297	V	0.15	0.03
298	V	0.84	0.07

Table S13. PARCH values of MLT residues

#	Name	Parch value	
		avg	std
1	G	0.89	0.14
2	I	0.18	0.03
3	G	0.09	0.02
4	A	0.04	0.02
5	V	0.01	0.00
6	L	0.11	0.05
7	K	0.54	0.11
8	V	0.01	0.00
9	L	0.00	0.00
10	T	0.07	0.04
11	T	0.04	0.02
12	G	0.01	0.00
13	L	0.00	0.00
14	P	0.01	0.01
15	A	0.01	0.01
16	L	0.01	0.00
17	I	0.01	0.01
18	S	0.02	0.01
19	W	0.08	0.01
20	I	0.00	0.01
21	K	0.77	0.11
22	R	0.90	0.07
23	K	1.15	0.08
24	R	1.37	0.21
25	Q	0.67	0.13
26	Q	0.92	0.03
27	G	1.20	0.19
28	I	0.40	0.14
29	G	0.09	0.05
30	A	0.04	0.01
31	V	0.03	0.02
32	L	0.08	0.03
33	K	0.61	0.06
34	V	0.03	0.02
35	L	0.01	0.00
36	T	0.03	0.01
37	T	0.05	0.05
38	G	0.00	0.00
39	L	0.01	0.00
40	P	0.01	0.00
41	A	0.01	0.00
42	L	0.01	0.00
43	I	0.00	0.01
44	S	0.04	0.03
45	W	0.05	0.01
46	I	0.00	0.00
47	K	0.93	0.22
48	R	1.11	0.24
49	K	0.54	0.03
50	R	1.25	0.24
51	Q	0.60	0.15
52	Q	2.25	0.11

Table S14. PARCH values of CLD5 residues

#	Name	Parch value	
		avg	std
1	M	0.92	0.03
2	G	0.21	0.09
3	S	0.01	0.01
4	A	0.11	0.01
5	A	0.02	0.01
6	L	0.00	0.01
7	E	0.08	0.02
8	I	0.03	0.02
9	L	0.01	0.00
10	G	0.00	0.00
11	L	0.00	0.00
12	V	0.01	0.00
13	L	0.01	0.00
14	C	0.00	0.00
15	L	0.01	0.01
16	V	0.01	0.00
17	G	0.00	0.00
18	W	0.07	0.02
19	G	0.00	0.00
20	G	0.00	0.00
21	L	0.08	0.02
22	I	0.02	0.01
23	L	0.05	0.02
24	A	0.02	0.02
25	C	0.09	0.01
26	G	0.01	0.01
27	L	0.15	0.03
28	P	0.02	0.01
29	M	0.05	0.03
30	W	0.25	0.03
31	Q	0.36	0.08
32	V	0.23	0.05
33	T	0.14	0.04
34	A	0.06	0.01
35	F	0.75	0.02
36	L	0.26	0.05
37	D	1.26	0.21
38	H	0.78	0.09
39	N	0.41	0.09
40	I	0.23	0.05
41	V	0.41	0.10
42	T	0.08	0.03
43	A	0.10	0.00
44	Q	0.36	0.02
45	T	0.09	0.01
46	T	0.21	0.02
47	W	0.23	0.05
48	K	0.92	0.15
49	G	0.00	0.00
50	L	0.04	0.02
51	W	0.17	0.04
52	M	0.12	0.04
53	S	0.07	0.01
54	C	0.00	0.01
55	V	0.09	0.03
56	V	0.21	0.02
57	Q	0.41	0.11
58	S	0.16	0.06
59	T	0.20	0.05
60	G	0.31	0.03
61	H	0.57	0.17
62	M	0.15	0.02
63	Q	0.18	0.02
64	C	0.13	0.03
65	K	0.58	0.04
66	V	0.16	0.03
67	Y	0.20	0.05
68	D	0.55	0.11
69	S	0.41	0.11
70	V	0.19	0.01
71	L	0.63	0.03
72	A	0.04	0.03
73	L	0.21	0.03
74	S	0.40	0.05
75	T	0.18	0.09
76	E	1.69	0.33
77	V	0.01	0.01
78	Q	0.36	0.09
79	A	0.17	0.02
80	A	0.05	0.01
81	R	0.30	0.03
82	A	0.01	0.01
83	L	0.01	0.00
84	T	0.04	0.01
85	V	0.01	0.00
86	S	0.01	0.00
87	A	0.00	0.00
88	V	0.01	0.01
89	L	0.05	0.00
90	L	0.01	0.00
91	A	0.00	0.00
92	F	0.05	0.01
93	V	0.01	0.00
94	A	0.00	0.00
95	L	0.04	0.01
96	F	0.02	0.00
97	V	0.00	0.00
98	T	0.00	0.00
99	L	0.05	0.03
100	A	0.06	0.01
101	G	0.00	0.00
102	A	0.03	0.01
103	Q	0.39	0.18
104	C	0.05	0.02
105	T	0.13	0.02
106	T	0.30	0.10
107	C	0.23	0.05
108	V	0.14	0.06
109	A	0.03	0.01
110	P	0.08	0.04
111	G	0.12	0.01
112	P	0.19	0.07
113	A	0.00	0.00
114	K	0.32	0.05
115	A	0.02	0.01
116	R	0.64	0.13
117	V	0.01	0.01
118	A	0.01	0.01
119	L	0.08	0.04
120	T	0.04	0.02

PARCH values of CLD5 residues (continued)

#	Name	Parch value	
		avg	std
121	G	0.00	0.00
122	G	0.00	0.00
123	V	0.01	0.00
124	L	0.00	0.00
125	Y	0.03	0.01
126	L	0.00	0.00
127	F	0.04	0.01
128	C	0.00	0.00
129	G	0.00	0.00
130	L	0.02	0.00
131	L	0.01	0.00
132	A	0.02	0.02
133	L	0.02	0.00
134	V	0.02	0.01
135	P	0.07	0.01
136	L	0.17	0.04
137	C	0.01	0.01
138	W	0.36	0.07
139	F	0.09	0.03
140	A	0.02	0.01
141	N	0.15	0.07
142	I	0.06	0.02
143	V	0.09	0.02
144	V	0.08	0.07
145	R	1.27	0.16
146	E	1.67	0.05
147	F	0.04	0.01
148	Y	0.50	0.15
149	D	0.92	0.12
150	P	0.28	0.05

#	Name	Parch value	
		avg	std
151	S	0.09	0.02
152	V	0.04	0.01
153	P	0.01	0.02
154	V	0.10	0.02
155	S	0.12	0.02
156	Q	0.11	0.03
157	K	0.49	0.09
158	Y	0.18	0.05
159	E	1.45	0.11
160	L	0.30	0.04
161	G	0.04	0.03
162	A	0.04	0.03
163	A	0.00	0.00
164	L	0.07	0.03
165	Y	0.30	0.07
166	I	0.03	0.03
167	G	0.00	0.00
168	W	0.09	0.01
169	A	0.02	0.01
170	A	0.00	0.00
171	T	0.00	0.00
172	A	0.01	0.00
173	L	0.01	0.00
174	L	0.00	0.00
175	M	0.01	0.01
176	V	0.01	0.00
177	G	0.00	0.00
178	G	0.00	0.00
179	C	0.00	0.01
180	L	0.00	0.00

#	Name	Parch value	
		avg	std
181	L	0.00	0.00
182	C	0.00	0.01
183	C	0.01	0.01
184	G	0.00	0.00
185	A	0.02	0.01
186	W	0.27	0.07
187	V	0.06	0.02
188	C	0.09	0.01
189	T	0.32	0.08
190	G	0.12	0.03
191	R	1.36	0.05
192	P	0.16	0.03
193	D	0.82	0.04
194	L	0.16	0.09
195	S	0.52	0.07

Table S15. PARCH values of AQP1 residues

#	Name	Parch value	
		avg	std
1	M	1.22	0.18
2	A	0.11	0.03
3	S	0.23	0.16
4	E	0.51	0.03
5	F	0.24	0.08
6	K	1.22	0.21
7	K	1.25	0.04
8	K	0.96	0.16
9	L	0.10	0.08
10	F	0.12	0.01
11	W	0.70	0.32
12	R	0.92	0.18
13	A	0.01	0.01
14	V	0.03	0.03
15	V	0.02	0.03
16	A	0.00	0.00
17	E	0.00	0.00
18	F	0.01	0.00
19	L	0.01	0.00
20	A	0.00	0.00
21	M	0.01	0.01
22	I	0.01	0.01
23	L	0.01	0.00
24	F	0.00	0.01
25	I	0.11	0.02
26	F	0.02	0.01
27	I	0.06	0.04
28	S	0.01	0.01
29	I	0.07	0.08
30	G	0.00	0.00
31	S	0.06	0.02
32	A	0.07	0.00
33	L	0.04	0.01
34	G	0.02	0.02
35	F	0.42	0.10
36	H	0.20	0.02
37	Y	0.07	0.04
38	P	0.51	0.15
39	I	0.58	0.08
40	K	1.77	0.20
41	S	0.44	0.11
42	N	0.56	0.14
43	Q	0.41	0.06
44	T	0.46	0.16
45	T	0.45	0.15
46	G	0.11	0.01
47	A	0.14	0.08
48	V	0.41	0.09
49	Q	0.08	0.03
50	D	0.30	0.02
51	N	0.32	0.08
52	V	0.08	0.04
53	K	0.71	0.02
54	V	0.02	0.02
55	S	0.00	0.00
56	L	0.03	0.02
57	A	0.03	0.01
58	F	0.08	0.04
59	G	0.00	0.00
60	L	0.08	0.02
61	S	0.00	0.00
62	I	0.02	0.01
63	A	0.00	0.00
64	T	0.01	0.00
65	L	0.00	0.01
66	A	0.00	0.01
67	Q	0.35	0.06
68	S	0.04	0.01
69	V	0.00	0.00
70	G	0.01	0.01
71	H	0.72	0.18
72	I	0.20	0.11
73	S	0.02	0.02
74	G	0.07	0.03
75	A	0.04	0.00
76	H	0.12	0.03
77	L	0.01	0.01
78	N	0.01	0.00
79	P	0.00	0.00
80	A	0.02	0.01
81	V	0.00	0.01
82	T	0.00	0.00
83	L	0.00	0.00
84	G	0.00	0.00
85	L	0.02	0.01
86	L	0.01	0.01
87	L	0.05	0.03
88	S	0.03	0.01
89	C	0.08	0.02
90	Q	0.42	0.02
91	I	0.13	0.05
92	S	0.43	0.06
93	V	0.42	0.14
94	L	0.47	0.16
95	R	0.44	0.17
96	A	0.00	0.00
97	I	0.06	0.04
98	M	0.15	0.08
99	Y	0.01	0.01
100	I	0.00	0.00
101	I	0.11	0.07
102	A	0.00	0.01
103	Q	0.00	0.00
104	C	0.00	0.00
105	V	0.01	0.01
106	G	0.01	0.01
107	A	0.02	0.01
108	I	0.00	0.00
109	V	0.00	0.00
110	A	0.01	0.01
111	T	0.03	0.01
112	A	0.00	0.01
113	I	0.02	0.00
114	L	0.00	0.01
115	S	0.07	0.03
116	G	0.03	0.01
117	I	0.07	0.02
118	T	0.00	0.00
119	S	0.22	0.07
120	S	0.16	0.07

PARCH values of APQ1 residues (continued)

#	Name	Parch value	
		avg	std
121	L	0.03	0.01
122	P	0.56	0.05
123	D	0.57	0.08
124	N	0.14	0.03
125	S	0.11	0.02
126	L	0.07	0.03
127	G	0.05	0.02
128	L	0.38	0.08
129	N	0.09	0.02
130	A	0.19	0.06
131	L	0.36	0.05
132	A	0.22	0.06
133	P	0.24	0.08
134	G	0.11	0.02
135	V	0.05	0.01
136	N	0.22	0.07
137	S	0.22	0.04
138	G	0.10	0.04
139	Q	0.10	0.03
140	G	0.00	0.00
141	L	0.02	0.01
142	G	0.00	0.00
143	I	0.06	0.03
144	E	0.03	0.01
145	I	0.00	0.00
146	I	0.03	0.02
147	G	0.00	0.00
148	T	0.00	0.00
149	L	0.00	0.00
150	Q	0.08	0.03

#	Name	Parch value	
		avg	std
151	L	0.00	0.00
152	V	0.00	0.00
153	L	0.02	0.02
154	C	0.00	0.00
155	V	0.00	0.01
156	L	0.01	0.01
157	A	0.01	0.01
158	T	0.02	0.01
159	T	0.38	0.05
160	D	0.51	0.13
161	R	1.37	0.25
162	R	1.65	0.34
163	R	1.21	0.07
164	R	1.85	0.70
165	D	0.24	0.04
166	L	0.07	0.02
167	G	0.07	0.03
168	G	0.16	0.05
169	S	0.26	0.10
170	G	0.05	0.03
171	P	0.13	0.06
172	L	0.07	0.03
173	A	0.00	0.00
174	I	0.03	0.03
175	G	0.00	0.00
176	F	0.05	0.02
177	S	0.01	0.01
178	V	0.00	0.00
179	A	0.00	0.00
180	L	0.09	0.07

#	Name	Parch value	
		avg	std
181	G	0.00	0.00
182	H	0.09	0.02
183	L	0.05	0.01
184	L	0.15	0.07
185	A	0.02	0.01
186	I	0.17	0.05
187	D	1.19	0.19
188	Y	0.24	0.00
189	T	0.00	0.00
190	G	0.08	0.04
191	C	0.00	0.00
192	G	0.04	0.01
193	I	0.02	0.01
194	N	0.04	0.00
195	P	0.10	0.03
196	A	0.00	0.00
197	R	0.32	0.03
198	S	0.00	0.00
199	F	0.02	0.01
200	G	0.00	0.00
201	S	0.00	0.00
202	S	0.00	0.00
203	V	0.01	0.01
204	I	0.06	0.02
205	T	0.09	0.01
206	H	0.45	0.15
207	N	0.10	0.03
208	F	0.24	0.09
209	Q	0.53	0.09
210	D	0.81	0.10

#	Name	Parch value	
		avg	std
211	H	0.08	0.02
212	W	0.15	0.08
213	I	0.02	0.02
214	F	0.04	0.01
215	W	0.13	0.04
216	V	0.02	0.01
217	G	0.00	0.00
218	P	0.11	0.01
219	F	0.20	0.01
220	I	0.00	0.00
221	G	0.00	0.00
222	A	0.00	0.00
223	A	0.00	0.00
224	L	0.01	0.00
225	A	0.00	0.00
226	V	0.02	0.02
227	L	0.01	0.00
228	I	0.02	0.00
229	Y	0.10	0.02
230	D	0.12	0.01
231	F	0.13	0.03
232	I	0.05	0.03
233	L	0.19	0.09
234	A	0.16	0.01
235	P	0.30	0.12
236	R	0.90	0.12
237	S	0.44	0.13
238	S	0.52	0.08
239	D	1.33	0.08
240	L	0.09	0.02

PARCH values of APQ1 residues (continued)

#	Name	Parch value	
		avg	std
241	T	0.17	0.06
242	D	0.86	0.06
243	R	0.42	0.04
244	V	0.01	0.01
245	K	1.02	0.17
246	V	0.16	0.03
247	W	0.17	0.03
248	T	0.21	0.03
249	S	1.11	0.10

Table S16. PARCH values of hGOAT residues

#	Name	Parch value	
		avg	std
1	M	1.61	0.05
2	E	2.02	0.18
3	W	0.52	0.17
4	L	0.19	0.05
5	W	0.47	0.06
6	L	0.07	0.03
7	F	0.17	0.05
8	F	0.21	0.07
9	L	0.10	0.02
10	H	0.32	0.04
11	P	0.03	0.03
12	I	0.35	0.10
13	S	0.09	0.02
14	F	0.09	0.07
15	Y	0.32	0.09
16	Q	0.03	0.02
17	G	0.00	0.01
18	A	0.01	0.01
19	A	0.03	0.03
20	F	0.05	0.02
21	P	0.05	0.01
22	F	0.01	0.00
23	A	0.02	0.03
24	L	0.01	0.00
25	L	0.01	0.00
26	F	0.00	0.00
27	N	0.01	0.01
28	Y	0.02	0.01
29	L	0.00	0.00
30	C	0.08	0.02
31	I	0.47	0.16
32	M	0.10	0.01
33	D	0.18	0.02
34	S	0.28	0.06
35	F	0.48	0.10
36	S	0.21	0.05
37	T	0.43	0.06
38	R	0.84	0.21
39	A	0.00	0.00
40	R	0.90	0.02
41	Y	0.21	0.09
42	L	0.05	0.04
43	F	0.01	0.02
44	L	0.07	0.05
45	L	0.00	0.01
46	T	0.03	0.01
47	G	0.00	0.00
48	G	0.00	0.00
49	G	0.00	0.00
50	A	0.01	0.00
51	L	0.00	0.01
52	A	0.00	0.00
53	V	0.04	0.01
54	A	0.03	0.01
55	A	0.18	0.04
56	M	0.15	0.07
57	G	0.02	0.01
58	S	0.33	0.13
59	Y	0.23	0.09
60	A	0.09	0.05
61	V	0.08	0.01
62	L	0.10	0.01
63	V	0.01	0.01
64	F	0.04	0.01
65	T	0.01	0.01
66	P	0.02	0.01
67	A	0.00	0.01
68	V	0.00	0.00
69	C	0.00	0.00
70	A	0.03	0.01
71	V	0.05	0.03
72	A	0.01	0.00
73	L	0.03	0.02
74	L	0.08	0.06
75	C	0.00	0.00
76	S	0.01	0.01
77	L	0.08	0.00
78	A	0.04	0.02
79	P	0.11	0.02
80	Q	0.96	0.06
81	Q	0.29	0.15
82	V	0.26	0.05
83	H	0.65	0.13
84	R	1.50	0.23
85	W	0.07	0.01
86	T	0.00	0.00
87	F	0.07	0.03
88	C	0.04	0.02
89	F	0.02	0.00
90	Q	0.04	0.01
91	M	0.03	0.03
92	S	0.01	0.01
93	W	0.03	0.01
94	Q	0.00	0.00
95	T	0.02	0.01
96	L	0.02	0.01
97	C	0.02	0.01
98	H	0.15	0.06
99	L	0.04	0.04
100	G	0.01	0.00
101	L	0.18	0.07
102	H	0.46	0.06
103	Y	0.08	0.01
104	T	0.00	0.01
105	E	0.51	0.07
106	Y	0.11	0.01
107	Y	0.14	0.04
108	L	0.43	0.17
109	H	0.65	0.12
110	E	1.61	0.18
111	P	0.06	0.03
112	P	0.28	0.13
113	S	0.27	0.03
114	V	0.52	0.17
115	R	0.78	0.13
116	F	0.07	0.02
117	C	0.02	0.00
118	I	0.08	0.04
119	T	0.09	0.06
120	L	0.04	0.03

PARCH values of hGOAT residues (continued)

#	Name	Parch value	
		avg	std
121	S	0.03	0.01
122	S	0.01	0.01
123	L	0.07	0.03
124	M	0.03	0.01
125	L	0.04	0.04
126	L	0.00	0.01
127	T	0.01	0.01
128	Q	0.33	0.03
129	R	0.23	0.02
130	V	0.04	0.01
131	T	0.03	0.01
132	S	0.02	0.01
133	L	0.22	0.04
134	S	0.00	0.01
135	L	0.13	0.02
136	D	0.25	0.03
137	I	0.15	0.05
138	C	0.15	0.04
139	E	1.37	0.07
140	G	0.24	0.03
141	K	1.06	0.07
142	V	0.06	0.02
143	K	1.37	0.08
144	A	0.09	0.04
145	A	0.17	0.03
146	S	0.15	0.05
147	G	0.01	0.01
148	G	0.19	0.07
149	F	1.58	0.23
150	R	1.21	0.04

#	Name	Parch value	
		avg	std
151	S	0.03	0.01
152	R	0.56	0.23
153	S	0.13	0.03
154	S	0.13	0.05
155	L	0.13	0.02
156	S	0.00	0.00
157	E	0.81	0.07
158	H	0.86	0.21
159	V	0.05	0.02
160	C	0.11	0.01
161	K	1.73	0.21
162	A	0.09	0.01
163	L	0.13	0.07
164	P	0.01	0.01
165	Y	0.08	0.02
166	F	0.02	0.01
167	S	0.01	0.01
168	Y	0.12	0.03
169	L	0.03	0.01
170	L	0.00	0.00
171	F	0.10	0.03
172	F	0.15	0.08
173	P	0.00	0.00
174	A	0.07	0.02
175	L	0.06	0.02
176	L	0.53	0.15
177	G	0.22	0.10
178	G	0.46	0.05
179	S	0.68	0.02
180	L	0.56	0.15

#	Name	Parch value	
		avg	std
181	C	0.13	0.04
182	S	0.19	0.05
183	F	0.08	0.00
184	Q	0.35	0.07
185	R	0.31	0.06
186	F	0.02	0.01
187	Q	0.05	0.02
188	A	0.13	0.01
189	R	0.22	0.08
190	V	0.21	0.06
191	Q	0.75	0.13
192	G	0.30	0.06
193	S	0.44	0.08
194	S	0.34	0.14
195	A	0.36	0.08
196	L	1.23	0.18
197	H	0.81	0.21
198	P	0.73	0.06
199	R	2.17	0.08
200	H	0.53	0.27
201	S	0.05	0.01
202	F	0.15	0.06
203	W	0.28	0.11
204	A	0.04	0.04
205	L	0.00	0.01
206	S	0.00	0.00
207	W	0.61	0.16
208	R	0.33	0.08
209	G	0.01	0.01
210	L	0.06	0.01

#	Name	Parch value	
		avg	std
211	Q	0.09	0.02
212	I	0.01	0.01
213	L	0.02	0.02
214	G	0.00	0.01
215	L	0.07	0.04
216	E	0.51	0.05
217	C	0.03	0.01
218	L	0.07	0.02
219	N	0.41	0.13
220	V	0.21	0.03
221	A	0.02	0.01
222	V	0.04	0.03
223	S	0.02	0.02
224	R	0.64	0.02
225	V	0.10	0.01
226	V	0.10	0.05
227	D	1.06	0.06
228	A	0.23	0.12
229	G	0.08	0.04
230	A	0.11	0.04
231	G	0.19	0.01
232	L	0.76	0.07
233	T	0.66	0.15
234	D	1.68	0.13
235	C	0.11	0.01
236	Q	0.39	0.08
237	Q	0.42	0.06
238	F	0.41	0.10
239	E	1.11	0.16
240	C	0.19	0.03

PARCH values of hGOAT residues (continued)

#	Name	Parch value	
		avg	std
241	I	0.20	0.07
242	Y	0.83	0.14
243	V	0.11	0.02
244	V	0.03	0.01
245	W	0.43	0.17
246	T	0.34	0.06
247	T	0.17	0.02
248	A	0.02	0.01
249	G	0.12	0.04
250	L	0.80	0.18
251	F	0.08	0.02
252	K	1.46	0.26
253	L	0.27	0.08
254	T	0.18	0.08
255	Y	0.10	0.01
256	Y	0.25	0.06
257	S	0.03	0.01
258	H	0.62	0.07
259	W	0.09	0.05
260	I	0.00	0.00
261	L	0.18	0.07
262	D	0.42	0.03
263	D	0.00	0.00
264	S	0.08	0.03
265	L	0.58	0.06
266	L	0.33	0.03
267	H	0.44	0.00
268	A	0.01	0.01
269	A	0.18	0.07
270	G	0.05	0.03

#	Name	Parch value	
		avg	std
271	F	0.33	0.17
272	G	0.25	0.01
273	P	0.35	0.08
274	E	0.58	0.06
275	L	0.04	0.00
276	G	0.10	0.02
277	Q	1.12	0.08
278	S	0.09	0.01
279	P	0.08	0.02
280	G	0.07	0.01
281	E	0.72	0.05
282	E	2.96	0.20
283	G	0.22	0.04
284	Y	1.91	0.37
285	V	0.14	0.02
286	P	0.15	0.03
287	D	0.30	0.05
288	A	0.23	0.09
289	D	1.15	0.21
290	I	0.28	0.08
291	W	0.75	0.11
292	T	0.17	0.05
293	L	0.29	0.11
294	E	1.62	0.16
295	R	1.61	0.26
296	T	0.97	0.26
297	H	1.53	0.20
298	R	1.79	0.28
299	I	0.82	0.26
300	S	0.29	0.12

#	Name	Parch value	
		avg	std
301	V	0.14	0.04
302	F	0.30	0.06
303	S	0.80	0.03
304	R	1.38	0.38
305	K	0.12	0.02
306	W	0.23	0.06
307	N	0.32	0.12
308	Q	0.04	0.00
309	S	0.02	0.00
310	T	0.60	0.05
311	A	0.60	0.11
312	R	0.72	0.15
313	W	0.79	0.27
314	L	0.03	0.02
315	R	1.16	0.12
316	R	1.51	0.10
317	L	0.17	0.02
318	V	0.10	0.02
319	F	0.24	0.02
320	Q	0.34	0.03
321	H	0.31	0.06
322	S	0.19	0.11
323	R	0.99	0.48
324	A	0.11	0.01
325	W	0.06	0.03
326	P	0.12	0.03
327	L	0.11	0.05
328	L	0.08	0.02
329	Q	0.19	0.03
330	T	0.05	0.01

#	Name	Parch value	
		avg	std
301	V	0.14	0.04
302	F	0.30	0.06
303	S	0.80	0.03
304	R	1.38	0.38
305	K	0.12	0.02
306	W	0.23	0.06
307	N	0.32	0.12
308	Q	0.04	0.00
309	S	0.02	0.00
310	T	0.60	0.05
311	A	0.60	0.11
312	R	0.72	0.15
313	W	0.79	0.27
314	L	0.03	0.02
315	R	1.16	0.12
316	R	1.51	0.10
317	L	0.17	0.02
318	V	0.10	0.02
319	F	0.24	0.02
320	Q	0.34	0.03
321	H	0.31	0.06
322	S	0.19	0.11
323	R	0.99	0.48
324	A	0.11	0.01
325	W	0.06	0.03
326	P	0.12	0.03
327	L	0.11	0.05
328	L	0.08	0.02
329	Q	0.19	0.03
330	T	0.05	0.01

PARCH values of hGOAT residues (continued)

#	Name	Parch value	
		avg	std
331	F	0.04	0.03
332	A	0.07	0.02
333	F	0.09	0.07
334	S	0.01	0.01
335	A	0.02	0.01
336	W	0.62	0.07
337	W	0.20	0.01
338	H	0.29	0.02
339	G	0.32	0.01
340	L	0.47	0.04
341	H	0.51	0.03
342	P	0.02	0.01
343	G	0.01	0.01
344	Q	0.19	0.05
345	V	0.05	0.01
346	F	0.05	0.01
347	G	0.04	0.01
348	F	0.06	0.04
349	V	0.03	0.01
350	C	0.01	0.01
351	W	0.20	0.09
352	A	0.00	0.01
353	V	0.01	0.00
354	M	0.04	0.01
355	V	0.01	0.01
356	E	0.61	0.10
357	A	0.00	0.01
358	D	0.92	0.03
359	Y	0.89	0.31
360	L	0.09	0.02

#	Name	Parch value	
		avg	std
361	I	0.06	0.03
362	H	0.63	0.04
363	S	0.05	0.03
364	F	0.24	0.11
365	A	0.03	0.01
366	N	0.49	0.15
367	E	2.63	0.28
368	F	0.68	0.09
369	I	1.09	0.06
370	R	1.44	0.27
371	S	0.20	0.08
372	W	0.53	0.17
373	P	0.12	0.06
374	M	0.05	0.02
375	R	1.79	0.24
376	L	0.17	0.04
377	F	0.09	0.00
378	Y	0.11	0.02
379	R	1.14	0.09
380	T	0.28	0.01
381	L	0.02	0.01
382	T	0.19	0.06
383	W	0.64	0.15
384	A	0.04	0.03
385	H	0.05	0.01
386	T	0.08	0.02
387	Q	0.04	0.00
388	L	0.18	0.03
389	I	0.04	0.01
390	I	0.03	0.02

#	Name	Parch value	
		avg	std
391	A	0.01	0.00
392	Y	0.16	0.06
393	I	0.20	0.01
394	M	0.14	0.05
395	L	0.02	0.01
396	A	0.04	0.00
397	V	0.49	0.05
398	E	1.73	0.11
399	V	0.08	0.03
400	R	1.78	0.30
401	S	0.12	0.05
402	L	0.06	0.03
403	S	0.04	0.01
404	S	0.03	0.02
405	L	0.01	0.00
406	W	0.16	0.05
407	L	0.03	0.02
408	L	0.06	0.01
409	C	0.01	0.01
410	N	0.08	0.04
411	S	0.08	0.04
412	Y	0.09	0.03
413	N	0.11	0.04
414	S	0.03	0.01
415	V	0.06	0.02
416	F	0.04	0.01
417	P	0.04	0.03
418	M	0.04	0.00
419	V	0.10	0.03
420	Y	0.12	0.01

#	Name	Parch value	
		avg	std
421	C	0.00	0.00
422	I	0.20	0.10
423	L	0.02	0.01
424	L	0.03	0.02
425	L	0.10	0.08
426	L	0.23	0.07
427	L	0.00	0.00
428	A	0.00	0.00
429	K	0.46	0.10
430	R	1.26	0.08
431	K	0.82	0.03
432	H	0.46	0.05
433	K	2.29	0.18
434	C	0.22	0.08
435	N	0.99	0.12
